# Historical Lattice Trees

**DOI:** 10.1007/s00220-023-04641-9

**Published:** 2023-01-31

**Authors:** Manuel Cabezas, Alexander Fribergh, Mark Holmes, Edwin Perkins

**Affiliations:** 1 Pontificia Universidad Católica de Chile, Santiago, Chile; 2 Université de Montréal, Montreal, Canada; 3 School of Mathematics and Statistics, The University of Melbourne, Melbourne, Australia; 4 Mathematics Department, The University of British Columbia, Vancouver, Canada

## Abstract

We prove that the rescaled historical processes associated to critical spread-out lattice trees in dimensions $d>8$ converge to historical Brownian motion. This is a functional limit theorem for measure-valued processes that encodes the genealogical structure of the underlying random trees. Our results are applied elsewhere to prove that random walks on lattice trees, appropriately rescaled, converge to Brownian motion on super-Brownian motion.

## Introduction and Main Results

In the past three decades, various critical high-dimensional spatial branching models have been conjectured or proved to converge to super-Brownian motion (SBM), which is a continuous Markov process taking values in the space of finite measures on $\mathbb {R}^d$. One of the settings in which significant progress has been made is that of critically weighted (and sufficiently spread-out) lattice trees (LT) above 8 dimensions [7, 10, 11, 17–19]. In particular, convergence on path space has recently been proved in this setting (see [11]). For LT’s convergence to SBM means weak convergence to SBM of the rescaled empirical measure process of the locations in the LT which are a given tree distance from the root. Hence the tree distance to the root plays the role of time for the stochastic processes. More recently, it has been proved in [20] that for LT’s, and in fact for several lattice models, the rescaled ranges (for LT’s the range is the compact set of vertices in the tree) converge weakly to the range of SBM. Convergence of genealogical observables is not forthcoming from the notions of weak convergence to SBM described thus far. Results of this kind can be obtained by proving convergence of the corresponding *“historical processes”* [6]. For LT’s this would mean that instead of just having the convergence to SBM of the rescaled empirical measure process of the particles in the LT, as a function of the distance from the root, one establishes convergence to historical Brownian motion (HBM) of the rescaled empirical measure process for the entire paths in the LT to the endpoints, as a function of the distance from the root. HBM, constructed in [6], is a process taking values in the space of finite measures on $\mathbb {R}^d$-valued paths, which at time *t* is the empirical measure of the past histories of the particles contributing to the SBM at time *t*. See Sect. 1.2.1 below for more about HBM, including the fact that is the weak limit of the rescaled historical processes associated with Branching Brownian Motion (Theorem 1.3). Our main result, Theorem 1.4 below, establishes this convergence of “historical processes” for LT’s.

In Sect. 2.1 we give a set of general conditions that are sufficient for convergence of discrete-time historical processes to HBM in the sense of finite-dimensional distributions (Theorem 2.1). Most of these conditions are already known to hold for a range of lattice models above the critical dimension including lattice trees ($d>8$) and oriented percolation ($d>4$), as well as for the voter model ($d\ge 2$) and the contact process ($d>4$), both of which are continuous-time models. The main condition that remains to be proved in each case is convergence of the joint characteristic functions of the increments of a finite dimensional subtree. These *detailed* *r-particle transforms* can be seen as enriched versions of the *r*-particle transforms studied e.g. in [13, 16, 17] (called Fourier transforms of $(r+1)$-point functions therein) that record genealogy. We prove that these conditions are satisfied for sufficiently spread-out lattice trees in high dimensions and so establish convergence to HBM in the sense of f.d.d.’s (Proposition 2.4). The required asymptotics of the detailed *r*-particle transforms are obtained via the *lace expansion* (see e.g. [25]) in Sect. 4. It is worth noting that these asymptotics can be understood from those of the usual *r*-particle transforms and the detailed 1-particle transform. In particular we do not require any new “diagrammatic estimates”. We believe that all of the conditions can also be verified for the other models^1^ mentioned above. For the voter model this is currently work in progress [1].

The second main ingredient in our proof is a novel tightness argument for historical processes which upgrades f.d.d. convergence to convergence on path space in a historical setting. This step is carried out in Sect. 3. We start with an abstract tightness result in a general historical setting (Theorem 3.6). For all of the lattice models mentioned above this reduces tightness of the approximating rescaled historical processes to that of the $\mathbb {R}$-valued processes obtained by integrating a test function (from an appropriate class) with respect to the rescaled historical processes. (Verification of the other conditions may be found in [20].) This key condition is then verified for LT’s with some effort in Proposition 3.11. The main ingredients of this argument are tightness of the total mass process from [11] and a uniform modulus of continuity for the approximating historical paths from [20]. The latter is in fact verified for all of the other lattice models mentioned above, and so we have potentially reduced the problem of tightness for historical processes to that of the total mass process for a range of other lattice models.

A simple consequence of our results is that the unique path in the tree from the origin to a uniformly chosen vertex (called the *backbone* from the origin to that vertex) of distance *n* converges weakly to BM on path space (see [18, Theorem 1.3]). Another application of our results concerns the scaling limit of random walk on lattice trees. In particular, the historical convergence proved herein is used in [21] to verify certain conditions of Ben-Arous et al. [2] which imply that random walk on lattice trees converges to a BM on a SBM cluster.

### Lattice trees and scaling limits

**Fig. 1:**
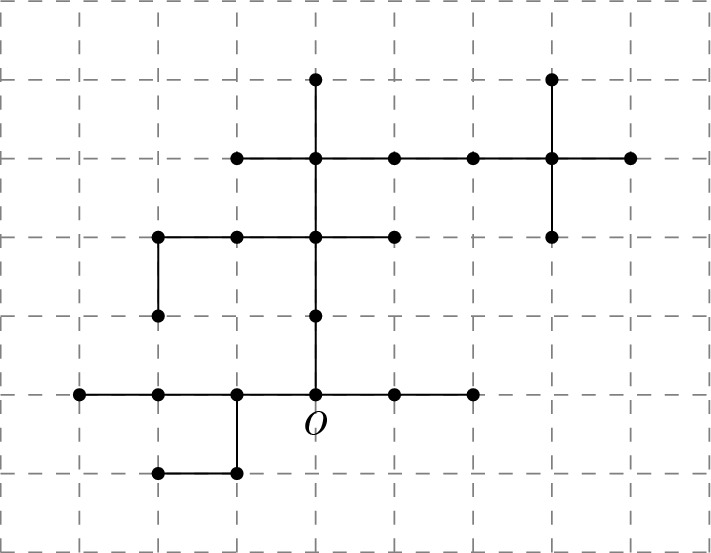
A (nearest neighbour) lattice tree in 2 dimensions

A lattice tree is a finite connected set of lattice bonds containing no cycles (see Fig. 1).

We will be considering lattice trees on $\mathbb {Z}^d$ with bonds connecting any two vertices that live in a common ball (in $\ell _\infty$) of sufficiently large radius $L\in \mathbb {N}$, and with $d>8$. To be more precise, let $d>8$ and let $D(\cdot )$ be the uniform distribution on a finite box $([-L,L]^d\setminus o)\cap \mathbb {Z}^d$, where $o=(0,\dots ,0)\in \mathbb {Z}^d$. The assumption of uniformity of *D* is not essential. We expect that the results herein hold for *D* as in [17, Section 1].

For a lattice tree $T\ni o$ define $W_{z,D}(T)=z^{|T|}\prod _{e\in T}D(e)$, where the product is over the edges in *T* and |*T*| is the number of edges in *T*.

#### Remark 1.1

If *T* is an edge-disjoint union of subtrees then $W_{z,D}(T)$ can be factored into a product over the weights of the subtrees.$\bigstar$

It turns out (see e.g. [10, 17]) that there exists a unique critical value $z_D$ such that $\rho =\sum _{T\ni o}W_{z_D,D}(T)<\infty$ and $\mathbb {E}[|\mathcal {T}|]=\infty$, where $\mathbb {P}(\mathcal {T}=T)=\rho ^{-1}W_{z_D,D}(T)$ for $T\ni o$. Hereafter we write $W(\cdot )$ for the critical weighting $W_{z_D,D}(\cdot )$ and suppose that we are selecting a random tree $\mathcal {T}\ni o$ according to this critical weighting.

Let *T* be a lattice tree containing $o\in \mathbb {Z}^d$, and for $m \in \mathbb {N}$, let $T_{m}$ denote the set of vertices in *T* of tree distance *m* from *o*. In particular, $T_0=\{o\}$, and for any $x\in T_{m}$ there is a unique path from *o* to *x* in the tree of length *m*. Roughly speaking, in this paper we consider the weak limit (as $m \rightarrow \infty$) of rescaled paths of this kind in high dimensions. For $t\in \mathbb {R}_+\setminus \mathbb {Z}_+$ define $T_t=T_{\lfloor t \rfloor }$. For $t\ge 0$ and $x\in \mathbb {Z}^d$ we will write $(t,x)\in T$ to mean that $x\in T_t$. The notation (*t*, *x*) is consistent with that in [20], while in the oriented percolation and contact process literature often (*x*, *t*) is used instead.

#### Functional limit theorems.

For our general discussion we require the notion of weak convergence of finite measures on Polish (i.e. complete, separable metric) spaces. We refer the reader to [8, Chapter 3] for further details on what we discuss below.

For a Polish space $\mathfrak {P}$, let $\mathcal {M}_F(\mathfrak {P})$ (resp. $\mathcal {M}_1(\mathfrak {P})$) denote the space of finite (resp. probability) measures on the Borel sets of $\mathfrak {P}$. For a sequence $\nu _n \in \mathcal {M}_F(\mathfrak {P})$ we say that $\nu _n$ converges weakly to $\nu \in \mathcal {M}_F(\mathfrak {P})$ and write $\nu _n \overset{w}{\longrightarrow }\nu$ if for every $f:\mathfrak {P}\rightarrow \mathbb {R}$ bounded and continuous,

$$\begin{aligned} \int f(x) \nu _n(dx)\rightarrow \int f(x) \nu (dx), \qquad \text { as }n \rightarrow \infty . \end{aligned}$$

Equipped with the Vasershtein metric, which generates the topology of weak convergence, $\mathcal {M}_F(\mathfrak {P})$ is also Polish (see e.g., [24, Ch. II]). We will use the notation $E_{\nu }[f(X)]$ for $\int f(x) \nu (dx)$, with the understanding that $X\in \mathfrak {P}$. This will be particularly convenient when *X* is a $\mathfrak {P}$-valued random variable defined on an underlying probability space and $\nu (\cdot )=c\cdot \mathbb {P}(X\in \cdot )$ for some $c>0$.

Let $S_n$ denote the location of a nearest-neighbour simple symmetric random walk on $\mathbb {Z}^d$ after *n* steps (starting from the origin $o\in \mathbb {Z}^d$). Then $\mathbb {E}[S_n^2]=n$ (here and elsewhere, for $x,y\in \mathbb {R}^d$ we abuse notation and write *xy* to mean $x\cdot y$, and hence $x^2$ to mean $|x|^2$) and the central limit theorem (CLT) states that $n^{-1/2}S_n$ converges in distribution to a random vector *Z* that is (multivariate-) normally distributed with mean $0\in \mathbb {R}^d$ and covariance matrix diag(1/*d*). Define probability measures $\nu _n$, $\nu$ on (the Borel sets of) $\mathbb {R}^d$ by

$$\begin{aligned} \nu _n(\cdot )=\mathbb {P}\big (n^{-1/2}S_n\in \cdot \big ), \quad \text { and }\quad \nu (\cdot )=\mathbb {P}(Z \in \cdot ).\end{aligned}$$

Phrased in the language of weak convergence of (finite) measures, the CLT says that $\nu _n\overset{w}{\longrightarrow }\nu$. The statement $\nu _n\overset{w}{\longrightarrow }\nu$ in $\mathcal {M}_F(\mathbb {R}^d)$ is well known to be equivalent to pointwise convergence of the characteristic functions (Fourier transforms), so for $\nu _n,\nu$ as above

$$\begin{aligned} \int {\textrm{e}}^{{\textrm{i}}kx}\nu _n(dx)\rightarrow \int {\textrm{e}}^{{\textrm{i}}kx}\nu (dx)={\textrm{e}}^{-\frac{k^2}{2d}}, \text { for }k\in \mathbb {R}^d.\end{aligned}$$

For a Polish space $\mathfrak {P}$ let $\mathcal {D}_t(\mathfrak {P})$ (resp. $\mathcal {D}(\mathfrak {P})$) denote the space of càdlàg paths (paths that are continuous from the right with limits existing from the left) mapping [0, *t*] (resp. $[0,\infty )$) to $\mathfrak {P}$. Let $\mathcal {C}_t(\mathfrak {P})$ (resp. $\mathcal {C}(\mathfrak {P})$) denote the corresponding subspace of continuous paths. It is well known that there are complete metrics on these spaces (generating the Skorokhod $J_1$ topology) for which $\mathcal {D}_t(\mathfrak {P})$ and $\mathcal {D}(\mathfrak {P})$ are also Polish (see [8, Chapter 3.5]). The *functional* central limit theorem (FCLT) concerns the entire path $(W^{\scriptscriptstyle (n)}_t)_{t\ge 0}$ defined by

$$\begin{aligned} W^{\scriptscriptstyle (n)}_t=n^{-1/2}S_{\lfloor nt \rfloor }. \end{aligned}$$

Defined in this way, for each *n*, $$W^{\scriptscriptstyle (n)}$$ jumps at times $$t=i/n$$ for $$i \in \mathbb {N}$$ and is constant on intervals $$[i/n,i+1/n)$$ for $$i\in \mathbb {Z}_+$$. In particular the process $$W^{\scriptscriptstyle (n)}$$ is a random element of the space $$\mathcal {D}(\mathbb {R}^d)$$ of càdlàg paths from $$\mathbb {R}_+=[0,\infty )$$ to $$\mathbb {R}^d$$. The FCLT states that the sequence of rescaled random walks $$(W^{\scriptscriptstyle (n)}_t)_{t\ge 0}$$ converges to a *d*-dimensional Brownian motion $$(B_t)_{t\ge 0}$$ (with $$B_1\sim \mathcal {N}(0,\text {diag}(1/d))$$). Phrased in the language of weak convergence of (probability) measures this FCLT says that $$\nu _{n}\overset{w}{\longrightarrow }\nu $$, where $$\nu _{n},\nu \in \mathcal {M}_1(\mathcal {D}(\mathbb {R}^d))$$ are defined by

$$\begin{aligned} \nu _{n}(\cdot )=\mathbb {P}\big ((W^{\scriptscriptstyle (n)}_{t})_{t\ge 0}\in \cdot \big ),\qquad \nu (\cdot )=\mathbb {P}\big ((B_{t})_{t\ge 0}\in \cdot \big ). \end{aligned}$$

Note that $\nu$ puts all its mass on continuous paths.

#### Paths and measure-valued processes for lattice trees.

For $(m,x)\in \mathcal {T}$ let $w_{}(m,x)= (w_{0}(m,x)=o,w_{1}(m,x),\dots , w_{m}(m,x)=x)$ denote the unique path in $\mathcal {T}$ from *o* to *x* in the tree. It is convenient to extend this to a function on $\mathbb {Z}_+$ and then to a function in $\mathcal {D}$ by writing

1.1 $$\begin{aligned} w_{n}(m,x):=w_{m}(m,x)=x, \text { for }n\ge m, \qquad w_{s}(m,x)=w_{\lfloor s \rfloor }(m,x), \quad \text { for }s \in [0,\infty ). \end{aligned}$$

Thus every $(m,x)\in \mathcal {T}$ has associated to it an infinite càdlàg path $w_{}(m,x)$ that is constant after time *m*. Denote the collection of ancestral paths for $\mathcal {T}$ by $\mathcal {W}=(w_{}(m,x))_{(m,x)\in \mathcal {T}}$. For $t\ge 0$ and $x\in \mathbb {Z}^d/\sqrt{n}$ such that $\sqrt{n}x \in \mathcal {T}_{nt}$ we define $w^{\scriptscriptstyle (n)}_{}(t,x)\in \mathcal {D}$ by

1.2 $$\begin{aligned} w^{\scriptscriptstyle (n)}_{s}(t,x)=\frac{w_{ns}(\lfloor nt \rfloor ,\sqrt{n}x)}{\sqrt{n}}, \quad \text { for }s\in [0,\infty ). \end{aligned}$$

By [10, 17] there exist constants $C_A,C_V>0$^2^ such that

1.3 $$\begin{aligned} \lim _{n \rightarrow \infty }\mathbb {E}[|\mathcal {T}_n|]= C_A, \text { and }\lim _{n\rightarrow \infty }n \mathbb {P}(|\mathcal {T}_n|>0)=2/(C_AC_V). \end{aligned}$$

Let $C_0=C_A^2C_V$, and let

**Fig. 2:**
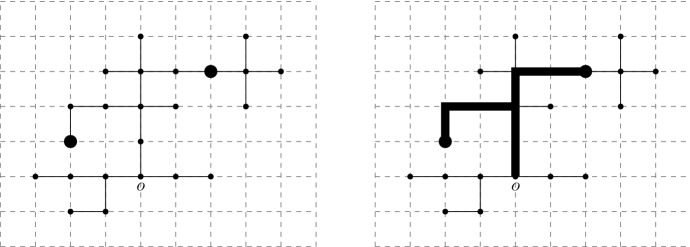
The MVP $X^{\scriptscriptstyle (1)}_5$ assigns masses to points in the tree at distance 5 from the root, while $H^{\scriptscriptstyle (1)}_5$ assigns the same masses to paths in the tree leading to these points

1.4 $$\begin{aligned} X^{\scriptscriptstyle (n)}_{t}=\,&\frac{1}{C_0n}\sum _{\sqrt{n}x \in \mathcal {T}_{nt}}\delta _x\in \mathcal {M}_F(\mathbb {R}^d), \qquad \text { and }\nonumber \\ H^{\scriptscriptstyle (n)}_t=\,&\frac{1}{C_0n}\sum _{\sqrt{n}x\in \mathcal {T}_{nt}}\delta _{{w^{\scriptscriptstyle (n)}_{}(t,x)}}\in \mathcal {M}_F(\mathcal {D}(\mathbb {R}^d)) \end{aligned}$$

denote the (rescaled) measure-valued “process” and historical “process” (see e.g. [6]) associated with the random lattice tree $\mathcal {T}$ respectively. Note that $X^{\scriptscriptstyle (n)}_{t}$ assigns mass to certain particles in the tree (but does not encode the genealogy) whereas $H^{\scriptscriptstyle (n)}_{t}$ assigns mass to genealogical paths leading to those particles. See e.g. Fig. 2.

For $\phi :\mathfrak {P} \rightarrow \mathbb {C}$ and $Y_t\in \mathcal {M}_F(\mathfrak {P})$ write $Y_t(\phi )=\int \phi dY_t$. Then for $\phi :\mathbb {R}^d \rightarrow \mathbb {C}$ we have

1.5 $$\begin{aligned} \int \phi (w_t)dH_t^{\scriptscriptstyle (n)}(dw)=X_t^{\scriptscriptstyle (n)}(\phi ), \end{aligned}$$

and in particular

$$\begin{aligned} H^{\scriptscriptstyle (n)}_{t}(1)\equiv X^{\scriptscriptstyle (n)}_{t}(1).\end{aligned}$$

Define the survival/extinction time as

$$\begin{aligned} S^{\scriptscriptstyle (n)}:=\inf \{t>0:X^{\scriptscriptstyle (n)}_t(1)=0\}=\inf \{t>0:H^{\scriptscriptstyle (n)}_t(1)=0\}.\end{aligned}$$

Let $C_1=C_AC_V$ so that from (1.3),

1.6 $$\begin{aligned} n\mathbb {P}(H^{\scriptscriptstyle (n)}_{t}(1)>0)=n\mathbb {P}(S^{\scriptscriptstyle (n)}>t)\rightarrow \frac{2}{C_1t}, \qquad \text { as }n\rightarrow \infty . \end{aligned}$$

Then we define $\nu ^{\scriptscriptstyle {\textrm{LT}}}_n\in \mathcal {M}_F(\mathcal {D}(\mathcal {M}_F(\mathbb {R}^d)))$ by

$$\begin{aligned} \nu ^{\scriptscriptstyle {\textrm{LT}}}_n(\bullet )=nC_1\mathbb {P}(X^{\scriptscriptstyle (n)}\in \bullet ), \end{aligned}$$

and $$\mu ^{\scriptscriptstyle {\textrm{LT}}}_{n}\in \mathcal {M}_F(\mathcal {D}(\mathcal {M}_F(\mathcal {D}(\mathbb {R}^d))))$$ by

1.7 $$\begin{aligned} \mu ^{\scriptscriptstyle {\textrm{LT}}}_{n}(\bullet )=nC_1\mathbb {P}(H^{\scriptscriptstyle (n)}\in \bullet ). \end{aligned}$$

Due to the survival probability asymptotics (1.6), multiplying by *n* and working on the event that the process survives until time *n* is asymptotically the same (up to a constant) as conditioning on survival until time *n* (or rescaled time 1).

According to [24, Section II.7], for any $\gamma ,\sigma ^2>0$ (representing the branching rate and diffusion parameter respectively) there exists a $\sigma$-finite measure $\mathbb {N}=\mathbb {N}^{\gamma ,\sigma ^2}$ on $\mathcal {C}(\mathcal {M}_F(\mathbb {R}^d))$, with $\mathbb {N}(X_t(1)> 0)=2/(\gamma t)$ such that $\mathbb {N}$ is the canonical measure associated to the $((B_t)_{t\ge 0},\gamma ,0)$-superprocess. Here $(B_t)_{t\in [0,\infty )}$ is a *d*-dimensional BM with $B_1\sim \mathcal {N}(0,\sigma ^2I_{d\times d})$, which is a (time-homogeneous) Markov process. The superprocess in question (called super-Brownian motion) is a measure-valued process that can be thought of as the empirical measures of an infinitesimal critical branching process whose spatial dispersion is governed by the $\mathbb {R}^d$-valued process $(B_t)_{t\ge 0}$. If $S=\inf \{t>0:X_t(1)=0\}$, then $\mathbb {N}$ is supported on $\{X\in \mathcal {C}(\mathcal {M}_F(\mathbb {R}^d)): X_0=0,S>0,X_t=0\ \forall t\ge S\}$, and so the above implies that

1.8 $$\begin{aligned} \mathbb {N}(S>t)=2/(\gamma t). \end{aligned}$$

By replacing the Markov process $(B_t)_{t\ge 0}$ with the path-valued (time-inhomogeneous) Markov process $(B_{[0,t]})_{t\ge 0}\equiv \big ((B_s)_{s\in [0,t]}\big )_{t\ge 0}$, and using the general theory of superprocesses, there also exists a $\sigma$-finite measure $\mathbb {N}_{\scriptscriptstyle H}=\mathbb {N}_{\scriptscriptstyle H}^{\gamma ,\sigma ^2}$ on $\mathcal {C}(\mathcal {M}_F(\mathcal {C}(\mathbb {R}^d)))$ with $\mathbb {N}_{\scriptscriptstyle H}(H_t(1)>0)=\mathbb {N}(S> t)$ such that $\mathbb {N}_{\scriptscriptstyle H}$ is the canonical measure associated to the $((B_{[0,t]})_{t\ge 0},\gamma ,0)$-superprocess. The latter (as well as the process *H* underlying $\mathbb {N}_{\scriptscriptstyle H}$) is called historical Brownian motion (HBM). The general construction of canonical measures for superprocesses may be found in [24, Section II.7], while Section II.8 therein shows how to consider the historical processes in this general framework. One can also construct $\mathbb {N}_{\scriptscriptstyle H}$ from the canonical measure of Le Gall’s Brownian snake since the historical process is a functional of the snake. See [22, pages 34, 64] for details.

It is proved in [11, 17] that for lattice trees in dimensions $d>8$ (with *L* sufficiently large) $\nu ^{\scriptscriptstyle {\textrm{LT}}}_n\overset{w}{\longrightarrow }\mathbb {N}$, where the parameters of $\mathbb {N}$ are $\gamma =1$ and $\sigma _0^2=\sigma _0^2(L,d)$, which is to be discussed later. Since the limit is a $\sigma$-finite measure, $\nu _n\overset{w}{\longrightarrow }\mathbb {N}$ is defined in terms of weak convergence of a family of finite measures (indexed by *t*) on $\mathcal {D}(\mathcal {M}_F(\mathbb {R}^d))$ as

1.9 $$\begin{aligned} \nu _n(\bullet ,S^{\scriptscriptstyle (n)}> t) \overset{w}{\longrightarrow }\mathbb {N}(\bullet , S>t),\qquad \text { for each }t> 0,\end{aligned}$$

or equivalently in terms of weak convergence of their conditional (on $$S>t$$) counterparts, which are probability measures. (The equivalence holds by (1.6), (1.7) and (1.8).) Similar results have been proved for other self-interacting branching systems such as the voter model [3, 4] ($$d\ge 2$$), oriented percolation (OP) [16] ($$d>4$$), and the contact process (CP) [13] ($$d>4)$$), although for OP and CP only convergence of the finite-dimensional distributions has been established and tightness remains an open problem. The corresponding result for the historical processes ($$\mu _{n}\overset{w}{\longrightarrow }\mathbb {N}_{\scriptscriptstyle H}$$) was an open problem in all of the above contexts. Here we resolve this problem for lattice trees ($$d>8$$, and *L* sufficiently large^3^), and, as was suggested above, our general approach may well also help in the other contexts above. A discussion of possible extensions and challenges for other models, including these, may be found in Sect. 1.3.

### Main results

In this section we state our main result (Theorem 1.4 below). For this, we first introduce some notation and present the relevant notions of weak convergence. We then introduce critical branching Brownian motion (BBM) as a simpler process from which one can understand the limiting historical Brownian motion through a corresponding historical limit theorem for rescaled BBM’s, see Theorem 1.3. The latter follows easily from results in the literature as we will describe. Following this, we state our main result. Theorem 1.3 is also used in the proof of our main result by identifying the joint characteristic functions of the general moment measures for the limiting HBM in Proposition 2.6.

For a Polish space $\mathfrak {P}$, and $\varvec{x}=(x_t)_{t\ge 0}\in \mathcal {D}(\mathcal {M}_F(\mathfrak {P}))$, let $S(\varvec{x})=\inf \{t>0:x_t(\mathfrak {P})=0\}$. Let $\mathcal {M}^{\scriptscriptstyle {\textrm{EX}}}(\mathfrak {P})$ (resp. $\mathcal {M}^{\scriptscriptstyle {\textrm{EX}}}_1(\mathfrak {P})$) denote the set of $\sigma$-finite (resp. probability) measures $\mu$ on $\mathcal {D}(\mathcal {M}_F(\mathfrak {P}))$ such that

1. $\mu \big (\{\varvec{x}:S(\varvec{x})>s\}\big )\in (0,\infty )$ for each $s>0$ and $\mu (\{\varvec{x}:S(\varvec{x})=\infty \})=0$, and
2. $$\mu \big (\{\varvec{x}:x_t(\mathfrak {P})>0 \text { for some }t>S(\varvec{x})\}\big )=0$$.

One should think of $\mathcal {M}^{\scriptscriptstyle {\textrm{EX}}}_1(\mathfrak {P})$ as the space of excursion measures for càdlàg measure-valued paths where the measures are on $\mathfrak {P}$. For $\mu \in \mathcal {M}^{\scriptscriptstyle {\textrm{EX}}}(\mathfrak {P})$, and $s>0$ define the (probability) measure $\mu ^s$ on $\mathcal {D}(\mathcal {M}_F(\mathfrak {P}))$ to be $\mu$ conditional on $S>s$, i.e.

$$\begin{aligned} \mu ^s(\bullet )=\dfrac{\mu (\bullet , \{\varvec{x}:S(\varvec{x})>s\})}{\mu (\{\varvec{x}:S(\varvec{x})>s\})}. \end{aligned}$$

For $r\in \mathbb {N}$ and ${t}=(t_1,\dots , t_r)\in [0,\infty )^r$ and a finite measure $\kappa$ on $\mathcal {D}(\mathcal {M}_F(\mathfrak {P}))$, let $\kappa _{{t}}$ denote the (finite) measure on $(\mathcal {M}_F(\mathfrak {P}))^r$ defined by

$$\begin{aligned} \kappa _{{t}}(\bullet )=\kappa (\{\varvec{x}:(x_{t_i})_{i=1}^r\in \bullet \}). \end{aligned}$$

#### Definition 1.2

(*Weak convergence*). Fix a sequence $(\mu _n)_{n \in \mathbb {N}\cup \{\infty \}}$ in $\mathcal {M}^{\scriptscriptstyle {\textrm{EX}}}(\mathfrak {P})$.

- We write $\mu _n\overset{w}{\longrightarrow }\mu _{\infty }$ as $n \rightarrow \infty$ if for every $s>0$, $\mu _n(S>s)\rightarrow \mu _{\infty }(S>s)$ and
  $$\begin{aligned} \mu _{n}^{s}\overset{w}{\longrightarrow }\mu ^s_{\infty }, \quad \text { in } \mathcal {M}_1\big (\mathcal {D}(\mathcal {M}_F(\mathfrak {P}))\big ). \end{aligned}$$
- We write $\mu _n\overset{f.d.d.}{\longrightarrow }\mu _{\infty }$ if for every $s>0$, $r\in \mathbb {N}$ and ${t}\in \mathbb {R}_+^r$, we have $\mu _n(S>s)\rightarrow \mu _{\infty }(S>s)$, and
  $$\begin{aligned} \mu _{n,{t}}^{s}\overset{w}{\longrightarrow }\mu _{\infty ,{t}}^{s}, \quad \text { in } \mathcal {M}_1\big ((\mathcal {M}_F(\mathfrak {P}))^r\big ).\end{aligned}$$

$$\blacktriangleleft $$

#### Branching Brownian motion.

A good way to understand historical Brownian motion is as a limit of critical branching Brownian motions. Recall that branching Brownian motion may be viewed as a system of Brownian motions run along the edges of a critical Galton–Watson tree. The notation introduced below is presented in [24] at a more leisurely pace. We start by defining a Brownian motion on a full binary tree. Let

1.10 $$\begin{aligned} I=\{\alpha =(\alpha _0,\dots ,\alpha _n):\alpha _0=0, \alpha _i\in \{0,1\}\text { for }i>0, n\in \mathbb {Z}_+\}, \end{aligned}$$

and for $\alpha$ as above set $|\alpha |=n$, $\alpha |i=(\alpha _0,\dots ,\alpha _i)$, $i\le n$, and say $\beta$ is an ancestor of $\alpha$ iff $\beta =\alpha |i$ for some $i<|\alpha |$. If $\alpha ,\beta \in I$, the greatest common antecedent (gca) of $\alpha$ and $\beta$ is $\alpha \wedge \beta =\alpha |i$, where *i* is the maximal integer such that $\alpha |i=\beta |i$. If $|\alpha |>0$, the parent of $\alpha$ is $\pi \alpha :=\alpha |(|\alpha |-1)$.

**Fig. 3:**
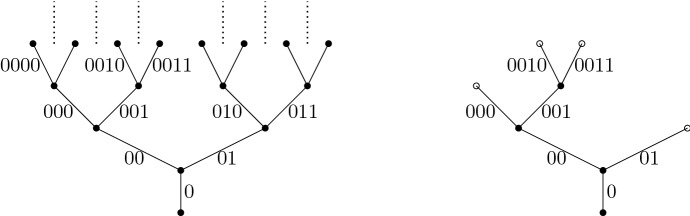
On the left is the index set *I* drawn (with labels as edges) up to and including generation 3. On the right is an example of a Galton–Watson tree (with edge labels $\alpha$), where $e^\alpha =0$ for all $\alpha \in \{000,0010,0011,01\}$, while $e^\alpha =2$ for $\alpha \in \{0,00,001\}$. Note that we have dropped the parentheses and commas in the notation for elements of *I* to declutter the pictures

Let $\{W^\alpha :\alpha \in I\}$ be iid *d*-dimensional Brownian motions with variance parameter $\sigma ^2$. For a fixed $n\in \mathbb {N}$ (dependence on *n* is suppressed) and for $\alpha \in I$, let

$$\begin{aligned} \hat{B}^\alpha _t=\sum _{i=0}^{|\alpha |}\int _0^t \mathbb {1}_{\{s\in [i/n,(i+1)/n)\}}dW^{\alpha |i}_s,\end{aligned}$$

and note that $(\hat{B}_t^\alpha )_{t\ge 0}$ is a *d*-dimensional Brownian motion, starting at 0, that runs until time $(|\alpha |+1)/n$ (after which it stays constant). We can view $\{\hat{B}_t^\alpha :t<(|\alpha |+1)/n, \alpha \in I\}$ as a Brownian motion run on a rescaled binary tree with edge lengths 1/*n*. We next prune the binary tree to make it a critical Galton–Watson (G–W) tree. Let $\{e^\alpha :\alpha \in I\}$ be a collection of iid random variables with (critical) binary offspring law $\frac{1}{2}\delta _0+\frac{1}{2}\delta _2$ that is independent of $\{W^\alpha :\alpha \in I\}$. For a fixed $n\in \mathbb {N}$ (dependence on *n* is suppressed) and for $\alpha \in I$, let

$$\begin{aligned} \tau ^\alpha =\min \Big \{\frac{i+1}{n}:e^{\alpha |i}=0\Big \}\quad (\min \emptyset =\frac{|\alpha |+1}{n}),\end{aligned}$$

and also define

$$\begin{aligned} B^\alpha _t={\left\{ \begin{array}{ll}\hat{B}^\alpha _t, &{} \text { if }t<\tau ^\alpha , \\ \Delta , &{}\text { if }t\ge \tau ^\alpha .\end{array}\right. }\end{aligned}$$

**Fig. 4:**
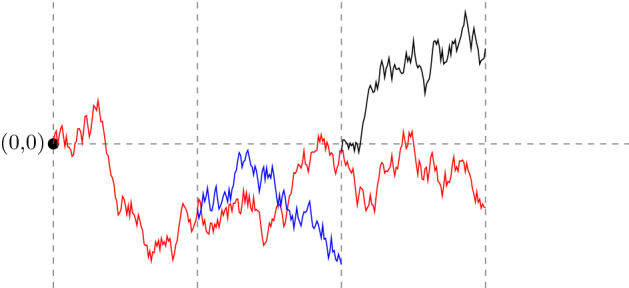
A (binary) branching Brownian motion in 1-dimension, with time on the *x* axis, drawn up to the third branch time, 3/*n*. In the corresponding G–W tree, the root 0 has two children, exactly one of which has 2 children

Here $\Delta$ is added to $\mathbb {R}^d$ as a cemetery point. In this way $GW=\{\alpha :\tau ^\alpha =\frac{|\alpha |+1}{n}\}$ labels the points (drawn as edges in Fig. 3) on a G–W tree with a critical binary offspring law that does not depend on *n*. We have scaled the edge lengths of the tree to be $n^{-1}$ and write $\alpha \sim t$ iff $\alpha \in GW$ and $\frac{|\alpha |}{n}\le t<\frac{|\alpha |+1}{n}$. Therefore $\alpha \sim t$ means that $\alpha$ labels an edge in the Galton–Watson tree which is alive at time $t\ge 0$. In particular, $0 \sim t$ for every $t<1/n$, see Fig. 3. Finally $\{B^\alpha _t:\alpha \sim t\}$ for $t\ge 0$ is a system of Brownian motions, starting with a single particle at the origin, and run along these edges while undergoing critical binary branching at times $\{j/n:j\in \mathbb {N}\}$, with the motions being independent along the disjoint scaled edges in the G–W tree. Figure 4 gives a depiction of the system of Brownian motions in 1-dimension.

We define the scaled empirical measures $X^{\scriptscriptstyle (n)}_\cdot \in \mathcal {D}(\mathcal {M}_F(\mathbb {R}^d))$ and $H^{\scriptscriptstyle (n)}_\cdot \in \mathcal {D}(\mathcal {M}_F(\mathcal {C}(\mathbb {R}^d)))$ associated with these locations and historical paths, respectively, by

$$\begin{aligned} X^{\scriptscriptstyle (n)}_t=\frac{1}{n}\sum _{\alpha \sim t}\delta _{B^\alpha _t},\quad H^{\scriptscriptstyle (n)}_t=\frac{1}{n}\sum _{\alpha \sim t}\delta _{B^\alpha _{\cdot \wedge t}},\ \ t\ge 0. \end{aligned}$$

It is easy to extend the above definitions to the setting of a general mean 1 finite variance $\gamma$ offspring law in place of the critical binary branching law above where we have $\gamma =1$ (see [24, Section II.3]). In this setting let $\nu _n^{\scriptscriptstyle {\textrm{BBM}}}=n\mathbb {P}(X^{\scriptscriptstyle (n)}\in \cdot )$ and $\mu ^{\scriptscriptstyle {\textrm{BBM}}}_{n}=n\mathbb {P}(H^{\scriptscriptstyle (n)}\in \cdot )$. We believe that the following limit result was first proved in [24], although part (b) was not stated explicitly there. The original construction of $\mathbb {N}=\mathbb {N}^{\gamma ,\sigma ^2}$ was done by Le Gall using his Brownian snake (see [22, Ch. IV] and the references therein) from which the result below was clear enough.

##### Theorem 1.3

As $$n \rightarrow \infty $$,

- $$\nu _n^{\scriptscriptstyle {\textrm{BBM}}}\overset{w}{\longrightarrow }\mathbb {N}^{\gamma ,\sigma ^2}$$;
- $$\mu _{n}^{\scriptscriptstyle {\textrm{BBM}}}\overset{w}{\longrightarrow }\mathbb {N}_{\scriptscriptstyle {H}}^{\gamma ,\sigma ^2}$$.

##### Proof

(a) is a special case of [24, Theorem II.7.3]. We also use Kolmogorov’s classical result on survival asymptotics for critical branching processes (eg. [24, Theorem II.1.1]).

(b) also follows from the same results, where [24, Section II.8] explains how to put the historical setting into the general framework of [24, Theorem II.7.3]. $\square$

An easy consequence of the above and the obvious analogue of (1.5) for branching Brownian motion is that *H* projects down to super-Brownian motion,

$$\begin{aligned} X_t(\cdot )=H_t(\{y\in \mathcal {C}(\mathbb {R}^d):y_t\in \cdot \})\quad \forall t\ge 0\ \ \mathbb {N}_{\scriptscriptstyle H}-\text {a.e.}. \end{aligned}$$

#### Lattice trees in high dimensions.

Our main result is that the functional limit theorem for historical processes in (b) above, continues to hold for lattice trees in high dimensions (the analogue of (a) was already noted in (1.9)). Recall the definition of $\mu _{n}$ from (1.7).

##### Theorem 1.4

For each $d>8$ there exists $L_0\ge 1$ such that: for every $L\ge L_0$, there exists $\sigma ^2_0=\sigma _0^2(L,d) >0$ such that $\mu ^{\scriptscriptstyle {\textrm{LT}}}_{n}\overset{w}{\longrightarrow }\mathbb {N}_{\scriptscriptstyle H}^{1,\sigma ^2_0}$.

Here, and throughout this work, the constant $\sigma _0^2$ is equal to $v\sigma ^2/d$ in [17, Theorem 3.7].

### Discussion

We finish this section with a brief discussion of extensions and applications of our results, and commentary on possible extensions to other models.

Our results are extended in [21] and used in [2] to prove weak convergence of rescaled random walk on lattice trees to a Brownian motion on a Super-Brownian motion cluster, the latter as defined in [5]. [2] reduces this latter result to the verification of two conditions. Roughly speaking, the first of these conditions is that if one chooses *K* points at random in the lattice tree, then the spatial tree generated by these *K* points, and suitably rescaled, converges (as the scaling parameter becomes large) to the random tree in $\mathbb {R}^d$ generated by choosing *K* paths independently at random according to $\int _0^\infty H_t(\cdot )\,dt$ (normalized by its total mass). One interprets this convergence in an appropriate metric space. The weak convergence in Theorem 1.4 is extended in [21] to joint convergence with *K* independently chosen paths as above, and moreover one can include the branch times and path lengths, to eventually obtain the required spatial tree convergence. The second condition states that in a certain precise sense the vertices of the rescaled tree generated by the *K* points become dense in the full rescaled lattice trees, uniformly in the scaling parameter, as *K* becomes large. This is also verified in [21] by using one of the inputs of our tightness argument, namely the modulus of continuity from [20] as stated in Condition 3.4 below.

One may ask about historical convergence in other contexts. This is most natural in cases where there are existing notions of time and ancestry in the model. Such notions exist in the voter model, where the parent of (*t*, *x*) is the corresponding point $(t',x')$ from which (*t*, *x*) most recently updated its vote, and also in the contact process where the parent of an infected particle is the infected particle which most recently infected it. In his PhD thesis, Tim Banova is using the methodology of Sect. 2 to prove historical convergence of the voter model in dimensions $d>2$ (for both nearest-neighbour and spread-out (finite range) models). We believe the methodology of Sect. 2 is also relevant for historical convergence of sufficiently spread-out contact processes for $d>4$. Results for convergence of empirical measures associated with high-dimensional contact processes (but not in the historical context) have relied on a time-discretisation argument and analysis of oriented percolation (OP) (see [13]).

In the context of OP, there is a natural notion of time, but ancestral paths are not unique because there can be multiple connections between vertices. One possible “remedy” is for each site (*n*, *x*) of generation *n* in the cluster of the origin to choose a parent uniformly at random from among sites of generation $n-1$ in the cluster that are connected to (*n*, *x*). We expect that the resulting historical process of sufficiently spread-out OP does converge to historical Brownian motion in dimensions $d>4$, but note that this process does not encode every connection in the cluster of the origin.

Another approach that one could take (which would also be relevant for percolation and lattice animals) is to define ancestral paths only in terms of pivotal bonds for connections. Pivotal bonds for a connection from (0, *o*) to (*n*, *x*) in oriented percolation, and from *o* to *x* in percolation and lattice animals (if such a connection exists) have a natural temporal ordering, as all paths from point to point must pass through these pivotal bonds in the same order. One could then define historical paths by e.g. linearly interpolating between these pivotal bonds. After appropriate scaling we expect that these historical processes would converge to historical Brownian motion in dimensions larger than the respective critical dimension. Section 2 below would be relevant in each of these contexts.

As has already been noted, except for the voter model [3, 4], tightness for any of these models has been a challenging problem even in the context of convergence of empirical measures to SBM, where it has only been established for high-dimensional lattice trees [11] with considerable effort. The proof of tightness for our historical lattice trees uses some bounds on the total mass of the rescaled LT’s from [11], and Conditions 2.3 and 2.4 which have also been shown in [20] for OP and the contact process. The additional special property of LT’s we use is a sub-Markov property, Lemma 3.15. It would be interesting to see if the proof of tightness can be carried out without this property. The reason is that then control of the total mass process should suffice to prove tightness, even in the historical context, for both the contact process and OP. For percolation and lattice animals, tightness through this historical approach, without even a uniform modulus continuity (Condition 2.3), still seems to be out of reach.

Finally, note that in this paper we have assumed that the step kernel $D(\cdot )$ is uniform on a large box. As noted earlier, the uniformity assumption is not essential. We suspect that *D* with unbounded support but $>2$ finite moments and with $d>d_c=8$ suffices for convergence to historical Brownian motion. In particular this ought to be true in the nearest-neighbour setting, but at present it would seem to be a considerable challenge (see e.g. [9]) to quantify some dimension $d_0$ above which this holds.

## Finite-Dimensional Distributions

### A general theorem

In what follows we write $\mathbb {N}_{\scriptscriptstyle H}$ for $\mathbb {N}_{\scriptscriptstyle H}^{\gamma ,\sigma ^2}$ where the branching variance $\gamma >0$ and the diffusion parameter $\sigma ^2>0$ are fixed throughout.

A collection of $\mathcal {G}$ of bounded continuous functions from $\mathfrak {P}$ to $\mathbb {C}$ is a *determining class* for $\mathcal {M}_F(\mathfrak {P})$ if whenever $\mu ,\mu '\in \mathcal {M}_F(\mathfrak {P})$ satisfy $\int g \mathrm d\mu = \int g \mathrm d\mu '$ for all $g \in \mathcal {G}$, then $\mu =\mu '$. The following is the path-valued analogue of [19, Theorem 2.6]:

#### Theorem 2.1

(F.d.d. convergence to historical BM). Let $\mu _n\in \mathcal {M}^{\scriptscriptstyle {\textrm{EX}}}(\mathfrak {P})$, where $\mathfrak {P}=\mathcal {D}(\mathbb {R}^d)$, and let $\mathcal {G}$ be a determining class for $\mathcal {M}_F(\mathcal {D}(\mathbb {R}^d))$ that contains 1 and is closed under complex conjugation. Assume

(i) for every $n\in \mathbb {N}$, $\mu _n\big (\sup _{t\ge 0}H_t(\{h:h_0\ne o\})\ne 0\big )=0$ (paths start at *o*)
(ii) for every $t>0$, $\mu _n(S>t) \rightarrow \mathbb {N}_{\scriptscriptstyle H}(S>t)$ (convergence of survival measures)
(iii) for every $t>0$, $E_{\mu _n}[H_t(\bullet )]\overset{w}{\longrightarrow }E_{\mathbb {N}_{\scriptscriptstyle H}}[H_t(\bullet )]$ (weak convergence of finite mean measures on $\mathcal {D}$), and for every $\varepsilon >0$, $\mu _n(H_0(1)>\varepsilon )\rightarrow 0$.
(iv) for every $\ell \in \mathbb {Z}_+$ and every ${t}\in (0,\infty )^{\ell }$, and every $\phi _1,\dots ,\phi _{\ell } \in \mathcal {G}$,
  2.1 $$\begin{aligned} \lim _{n\rightarrow \infty }E_{\mu _n}\left[ \prod _{j=1}^\ell H_{t_j}(\phi _j)\right] = E_{\mathbb {N}_{\scriptscriptstyle H}}\left[ \prod _{j=1}^\ell H_{t_j}(\phi _j)\right] <\infty . \end{aligned}$$

Then $$\mu _{n}\overset{f.d.d.}{\longrightarrow }\mathbb {N}_{\scriptscriptstyle H}$$.

Note that $\mathbb {N}_{\scriptscriptstyle H}\big (\sup _{t\ge 0}H_t(\{h:h_0\ne o\})\ne 0\big )=0$. The following elementary consequence of [24, (II.8.6)(a)] states that both the mean measure at time *t* under $\mathbb {N}_{\scriptscriptstyle H}$, and the mean measure to a uniformly chosen point at time *t* conditional on survival until time *t*, are Wiener measure (i.e. the law of Brownian motion) for paths on [0, *t*]:

#### Lemma 2.2

The historical canonical measure $\mathbb {N}_{\scriptscriptstyle H}=\mathbb {N}^{1,\sigma ^2}_{\scriptscriptstyle H}$ satisfies

$$\begin{aligned} E_{\mathbb {N}_{\scriptscriptstyle H}}[H_t(\bullet )]=P(B_{[0,t]}\in \bullet )=E_{\mathbb {N}_{H}^{t}}\left[ \frac{H_t(\bullet )}{H_t(1)}\right] , \quad \forall t>0, \end{aligned}$$

where under *P*, $B_{[0,t]}=(B_s)_{s\in [0,t]}$ is a *d*-dimensional BM on $[0,\infty )$ (with $B_1\sim \mathcal {N}(0,\text {{diag}}(\sigma ^2))$) stopped at time $t>0$.

The proof of Theorem 2.1 is a simple adaption of the proof of [19, Theorem 2.6].

#### Sketch proof of Theorem 2.1

The only substantial change to the proof of [19, Theorem 2.6] is in the proof of tightness [19, Lemma 3.3].

If $t,\eta >0$, by (iii) there exists a compact set $K=K_{t,\eta }\subset \mathcal {D}$ such that

$$\begin{aligned} \sup _n E_{\mu _n}\left[ H_t(K^c)\right] <\eta ^2, \end{aligned}$$

and so by Markov’s inequality

2.2 $$\begin{aligned} \sup _n \mu _n(H_t(K^c)>\eta )<\eta . \end{aligned}$$

Fix $s>0$. Since $\mu _n(H_s(1)>0)\rightarrow 2/s$ we may find $n_s\in \mathbb {N}$ and $c_s>0$ so that $\inf _{n\ge n_s} \mu _n(H_s(1)>0)>c_s$. If $\varepsilon >0$ we may now use (2.2) and argue as in the proof of [19, Lemma 3.3] to find a compact set $\textbf{K}=\textbf{K}_{t,\varepsilon }\in \mathcal {M}_F(\mathcal {D})$ such that

$$\begin{aligned} \sup _n\mu _n(H_t\in \textbf{K}^c)<\varepsilon c_s, \end{aligned}$$

and hence (working now with the conditional measures) for $t>0$,

$$\begin{aligned} \sup _{n\ge n_s}\mu _n^{s}(H_t\in \textbf{K}^c)<\varepsilon . \end{aligned}$$

It follows that for any ${t}\in (0,\infty )^\ell$, $\big (\mu _{n,{t}}^{s}\big )_{n \in \mathbb {N}}$ is tight in $\mathcal {M}_F(\mathcal {D})^{\ell }$. Assume $\mu \in \mathcal {M}_F\big (M_F(\mathcal {D})^{\ell }\big )$ is a limit point of $(\mu ^s_{n,{t}})_{n\in \mathbb {N}}$. Then it follows from (2.1) and Dominated Convergence that

$$\begin{aligned} E_{\mu }\left[ \prod _{i=1}^\ell H_{t_i}(\phi _{i})\right] =\mathbb {N}^s_{{\scriptscriptstyle {H}},{t}}\left[ \prod _{i=1}^\ell H_{t_i}(\phi _{i})\right] \qquad \forall \phi _{1},\dots ,\phi _{\ell }\in \mathcal {G}. \end{aligned}$$

By [8, Proposition 3.4.6] it follows that $\mu =\mathbb {N}^s_{\scriptscriptstyle {H},{t}}$. Although this result is stated in [8] for $\mathcal {G}$ a set of real-valued functions, the fact that $\mathcal {G}$ is closed under complex conjugation allows one to see it is also a determining class for complex-valued measures and the proof in [8] then adapts easily to the complex-valued set of functions $\mathcal {G}$. It follows that $\mu _{n,{t}}^s\overset{w}{\longrightarrow }\mathbb {N}^s_{{\scriptscriptstyle H},{t}}$ for all ${t}\in (0,\infty )^\ell$. Hypothesis (iii) implies that under $\mu _n^s$, $H_0$ converges to the zero measure, which is also equal to $H_0$ under $\mathbb {N}^s_{\scriptscriptstyle H}$. Thus, $\mu _{n,{t}}^s\overset{w}{\longrightarrow }\mathbb {N}^s_{{\scriptscriptstyle H},{t}}$ for all ${t}\in [0,\infty )^\ell$, as required. $\square$

For ${s}=(s_0,\dots ,s_m)$, where $0=s_0<\dots <s_m$ and ${k}=(k_0,k_1,\dots ,k_m)\in \mathbb {R}^{d(m+1)}$ define $\phi _{{s},{k}}:\mathcal {D}\rightarrow \mathbb {C}$ by

2.3 $$\begin{aligned} \phi _{{s},{k}}(w)={\textrm{e}}^{{\textrm{i}}k_0w_{s_0}}\prod _{j=1}^m {\textrm{e}}^{{\textrm{i}}k_j(w_{s_j}-w_{s_{j-1}})}, \end{aligned}$$

and let $\mathcal {G}^*=\big \{\phi _{{s},{k}}: {s},{k} \text { as above for some }m \in \mathbb {N}\big \}$. Note that $\mathcal {G}^*$ is a determining class for $\mathcal {M}_F(\mathcal {D}(\mathbb {R}^d))$ since finite measures on $\mathcal {D}(\mathbb {R}^d)$ are determined by their finite-dimensional distributions, and the laws of these finite-dimensional random vectors are determined by the characteristic functions of appropriate dimension. The elements of $\mathcal {G}^*$ are precisely those which correspond to the characteristic function of the increments of the path at all finite sets of times. Setting ${k}={0}$ we see that $1\in \mathcal {G}^*$ and by replacing $k_j$ with $-k_j$ we observe that $\mathcal {G}^*$ is closed under complex conjugation. So we see that $\mathcal {G}^*$ satisfies the conditions on $\mathcal {G}$ in Theorem 2.1.

#### Remark 2.3

Under $\mathbb {N}_{\scriptscriptstyle H}$, $H_t$ assigns mass only to paths that are constant from time *t* onwards and start at *o* at time 0. The same holds for $H^{\scriptscriptstyle (n)}_t$ for all *n* for LT and BBM. Therefore, when applying Theorem 2.1 in these settings, with $\mathcal {G}=\mathcal {G}^*$ as above, we may restrict our attention to $\phi _{{s}^{\scriptscriptstyle (1)},{k}^{\scriptscriptstyle (1)}}, \dots , \phi _{{s}^{\scriptscriptstyle (\ell )},{k}^{\scriptscriptstyle (\ell )}}\in \mathcal {G}^*$ in part (iv) of the theorem satisfying $s^{\scriptscriptstyle (i)}_j\le t_i$ for each *i*, *j* and $k_0^{(i)}=0$. The latter means we can set $k^{(i)}=(k^{(i)}_1,\dots ,k^{(i)}_m)\in \mathbb {R}^{dm}$ and ignore the first factor in (2.3). Moreover we can without loss of generality assume that that the largest element of ${s}^{\scriptscriptstyle (i)}$ is $t_i$ for each *i* (i.e., if not we can append an extra component $t^{\scriptscriptstyle (i)}$ to ${s}^{\scriptscriptstyle (i)}$ and set the corresponding $k^{\scriptscriptstyle (i)}_j$ equal to zero without changing $\phi _{{s}^{\scriptscriptstyle (i)},{k}^{\scriptscriptstyle (i)}}$). $\bigstar$

In the context of Theorem 1.4, we will use Theorem 2.1 with the determining class $\mathcal {G}^*$ at the end of this section to first establish the following result:

#### Proposition 2.4

For $d>8$ there is an $L_0\ge 1$ so that for $L\ge L_0(d)$ there is a $\sigma _0^2(L,d)>0$ for which

$$\begin{aligned} \mu ^{\scriptscriptstyle {\textrm{LT}}}_n\overset{f.d.d.}{\longrightarrow }\mathbb {N}_{\scriptscriptstyle H}. \end{aligned}$$

Indeed, condition (i) of Theorem 2.1 trivially holds for lattice trees rooted at the origin. Condition (ii) of the Theorem is (1.6). The first part of Condition (iii) holds by [18, Theorem 2.1], and the second part is obvious because under $\mu _n^{\scriptscriptstyle {\textrm{LT}}}$, $H_0(1)=\frac{1}{C_0n}$. Condition (iv) of the Theorem (for the determining class $\mathcal {G}^*$) will follow immediately from Proposition 2.6 and Theorem 2.7 below. In order to state these results we need to introduce various notation, which we proceed to do now.

The *degree* of a vertex in a graph is the number of incident edges. Vertices of degree 1 are called *leaves*. Vertices of degree $\ge 3$ are called * branch points*.

**Fig. 5:**
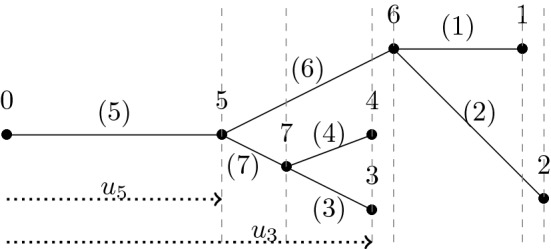
A depiction of a shape ${\digamma }\in \Sigma _4$ with vertex labels above vertices and edge labels in brackets. The set of edges in the path from vertex 0 to vertex 1 is $\mathcal {E}_1({\digamma })=\{1,5,6\}$. Variables $u_i$ are associated to each of the vertices *i*, describing a ‘length’ from 0 to *i*, to form $\mathcal {T}({\digamma },{u})$. Differences in these $u_i$ are then the “edge lengths”

#### Definition 2.5

A *non-degenerate shape* is an isomorphism class of finite connected rooted tree graphs whose vertices all have degree 1 or 3, and whose $r+1$ leaves (for some $r\ge 1$) are labelled $0, 1, 2,\dots , r$: the root 0 is always one of the leaves. To be more precise, two such graphs are considered to be the same shape if there is a graph isomorphism which preserves the labelling of the leaves (thus there is exactly one shape with 3 leaves and exactly 3 shapes with 4 leaves).

We let $\Sigma _r$ denote the set of non-degenerate shapes with $r+1$ leaves. For any ${\digamma }\in \Sigma _r$, we know that $\alpha$ has $r-1$ branch points, 2*r* vertices and $2r-1$ edges. Label the branch points as $r+1 ,\dots ,2r-1$ in order, as you encounter them as you move from the root to vertex 1, then continue to label new internal vertices in the order that you encounter them as you move from the root to vertex 2 and so on up to vertex *r*. See e.g. Fig. 5. This is just a convenient arbitrary but fixed order. For $i,j\in \{0,\dots ,2r-1\}$, we abuse the notation for the usual order and let $i\wedge j\in \{0,\dots ,2r-1\}$ denote the greatest common antecedent (gca) of *i* and *j*. The edges *e* of ${\digamma }\in \Sigma _r$ are labelled as $\mathcal {E}({\digamma })=\{1,\dots ,2r-1\}$ corresponding to the vertex labelling of the endvertex of *e* that is farthest from the root. For $e,f\in \mathcal {E}({\digamma })$, write $e\prec f$ if *e* is an ancestor of *f* in ${\digamma }$.

For leaves $\ell \in 1,\dots , r$, let $\mathcal {E}_\ell ({\digamma })$ be the set of edges in the unique path in ${\digamma }$ from *o* to $\ell$.

For ${\digamma }\in \Sigma _r$ we assign edge lengths by letting ${u}=(u_1,\dots ,u_{2r-1})\in (0,\infty )^{2r-1}$ give the distances from the vertices to the root. That is, $u_i$ is the distance from the root to vertex *i*, and the edge lengths can be found by differencing. We let $\mathbb {T}({\digamma },{u})$ denote the resulting tree with shape ${\digamma }$ and edge lengths ${u}$. See Fig. 5. We often will specify the distances ${t}=(t_1,\dots ,t_r)\in (0,\infty )^r$ of the *r* leaves to the root in advance. In this case we let $\mathcal {M}({t},{\digamma })$ denote the set of possible vertex distances from the root. That is, $\mathcal {M}({t},{\digamma })$ denotes the set of ${u}=(u_1,\dots ,u_{2r-1})\in (0,\infty )^{2r-1}$ such that:

2.4 $$\begin{aligned}&u_i=t_i, \text { for }i=1,\dots ,r \, ; \end{aligned}$$

2.5 $$\begin{aligned}&\text { if}\, k\,\text { and}\, j\,\text { are vertices of}\, {\digamma }\,\text {and}\, k\,\text { is an ancestor of}\, j\,\text {in }\,{\digamma },\,\text { then}\, u_k<u_j. \end{aligned}$$

$$\blacktriangleleft $$

**Fig. 6:**
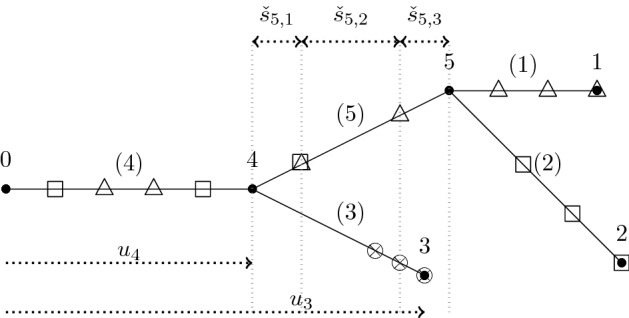
The tree $\mathbb {T}({\digamma },{u})$ together with times $\varvec{s}$. The ($m^{\scriptscriptstyle (1)}=7$) $\triangle$ symbols represent times $s^{\scriptscriptstyle (1)}_1,\ldots , s^{\scriptscriptstyle (1)}_{7}$. Similarly $\square$ symbols represent times $s^{\scriptscriptstyle (2)}_j$ (with $m^{\scriptscriptstyle (2)}=6$) and $\otimes$ symbols represent times $s^{\scriptscriptstyle (3)}_j$ (with $m^{\scriptscriptstyle (3)}=3$) respectively. In this example there is one point (on edge 5) that is both square and triangle simultaneously. The ‘subinterval’ lengths $\check{s}_{5,i}$ are indicated for edge 5

Consider a given (non-degenerate) shape ${\digamma }\in \Sigma _r$, ${t}\in (0,\infty )^r$, and ${u}\in \mathcal {M}({t},{\digamma })$ as above. Let ${\varvec{s}}=({s}^{\scriptscriptstyle (1)},\dots ,{s}^{\scriptscriptstyle (r)})$, where ${s}^{\scriptscriptstyle (\ell )}=(s_0^{\scriptscriptstyle (\ell )},\dots ,s^{\scriptscriptstyle (\ell )}_{m^{\scriptscriptstyle (\ell )}})$, and $0=s_0^{\scriptscriptstyle (\ell )}<s_1^{\scriptscriptstyle (\ell )}<\dots <s_{m^{\scriptscriptstyle (\ell )}}^{\scriptscriptstyle (\ell )}= t_\ell$ for each $\ell \in [r]:=\{1,\dots ,r\}$. If $e \notin \mathcal {E}_\ell ({\digamma })$ then set $\mathcal {I}(e ,{s}^{\scriptscriptstyle (\ell )})=\varnothing$. If $e\in \mathcal {E}_\ell ({\digamma })$, then let $\mathcal {I}(e ,{s}^{\scriptscriptstyle (\ell )})$ denote those elements of ${s}^{\scriptscriptstyle (\ell )}$ that fall in the interval $(u_-(e),u_+(e))$, where $u_-(e), u_+(e)$ are the elements of ${u}$ corresponding to the endvertices of *e* (if *e* is adjacent to the root, then set $u_-(e)=0$). Let $\mathcal {I}(e,\varvec{s})=\cup _{\ell =1}^r\mathcal {I}(e,{s}^{\scriptscriptstyle (\ell )})$. The $j(e):=|\mathcal {I}(e,\varvec{s})|$ elements of $\mathcal {I}(e,\varvec{s})$ divide the interval $[u_-(e),u_+(e)]$ into $j_e:=j(e)+1$ subintervals - denote their lengths by $(\check{s}_{e,k})_{k=1,\dots , j_e}$ and set $\check{\varvec{s}}=(\check{s}_{e,k})_{e\in \mathcal {E}({\digamma });k=1,\dots ,j_e}$. If $j(e)=0$ then $\check{s}_{e,1}=u_+(e)-u_-(e)$. Note that *j*(*e*) and $\check{\varvec{s}}$ depend on ${\digamma },{u}, \varvec{s}$. See Fig. 6.

For $\ell \in [r]$, $e\in \mathcal {E}_{\ell }({\digamma })$, and $a \in \{1,\dots ,j_e\}$, let

$$\begin{aligned} \zeta ^{[e]}(a,\ell )=\min \Bigg \{i \le m^{(\ell )}: s^{\scriptscriptstyle (\ell )}_{i}\ge u_-(e) +\sum _{i_e=1}^a\check{s}_{e,i_e}\Bigg \}.\end{aligned}$$

Given ${k}^{\scriptscriptstyle (\ell )}=(k_1^{\scriptscriptstyle (\ell )},\dots ,k^{\scriptscriptstyle (\ell )}_{m^{\scriptscriptstyle (\ell )}})\in (\mathbb {R}^d)^{m^{\scriptscriptstyle (\ell )}}$, for each $\ell \in [r]$, and for $e\in \{1,\dots ,2r-1\}$ and $a\le j_e$, let

$$\begin{aligned} \check{k}_{e,a}=\sum _{\ell :e\in \mathcal {E}_\ell ({\digamma })} k^{\scriptscriptstyle (\ell )}_{\zeta ^{[e]}(a,\ell )}. \end{aligned}$$

For given $\sigma ^2>0$, $r\in \mathbb {N}$, ${\digamma }\in \Sigma _r$, ${u}\in \mathcal {M}({t},{\digamma })$ for some ${t}\in (0,\infty )^r$, and for given ${\varvec{k}}=({k}^{\scriptscriptstyle (1)},\dots ,{k}^{\scriptscriptstyle (r)})$ and $\varvec{s}=(s_0^{\scriptscriptstyle (\ell )},\dots ,s^{\scriptscriptstyle (\ell )}_{m^{\scriptscriptstyle (\ell )}})$ (where, for $\ell \in [r]$, $m^{\scriptscriptstyle (\ell )}\in \mathbb {N}$, ${s}^{\scriptscriptstyle (\ell )}=(0=s^{\scriptscriptstyle (\ell )}_0,s^{\scriptscriptstyle (\ell )}_1,\dots , s^{\scriptscriptstyle (\ell )}_{m^{\scriptscriptstyle (\ell )}}=t_\ell )$, ($s^{\scriptscriptstyle (\ell )}_i<s^{\scriptscriptstyle (\ell )}_{i+1}$), ${k}^{\scriptscriptstyle (\ell )}\in \mathbb {R}^{m(\ell )}$), define

2.6 $$\begin{aligned} \Phi _{\sigma ^2}({\digamma },{u},{\varvec{s}}, {\varvec{k}})=\prod _{e=1}^{2r-1}\prod _{i=1}^{j_e}\exp \Big (\frac{-\sigma ^2|\check{k}_{e,i}({\digamma }, {u},\varvec{s})|^2\check{s}_{e,i}({\digamma },{u},\varvec{s})}{2}\Big ). \end{aligned}$$

The following proposition (proved in Sect. 2.2) gives an explicit formula for the right hand side of (2.1). The integral over $\mathcal {M}({t},{\digamma })$ is actually an $(r-1)$-dimensional integral over $(u_{r+1},\dots ,u_{2r-1})$ as the first *r* components are fixed.

#### Proposition 2.6

For any $r\in \mathbb {N}$, ${t}\in (0,\infty )^{r}$ and $\phi ^{\scriptscriptstyle (1)},\dots , \phi ^{\scriptscriptstyle (r)} \in \mathcal {G}^*$ (with $\phi ^{\scriptscriptstyle (\ell )}=\phi _{{s}^{\scriptscriptstyle (\ell )},{k}^{\scriptscriptstyle (\ell )}}$ where ${s}^{\scriptscriptstyle (\ell )}=(s^{\scriptscriptstyle (\ell )}_0=0,s^{\scriptscriptstyle (\ell )}_1,\dots , s^{\scriptscriptstyle (\ell )}_{m^{\scriptscriptstyle (\ell )}}=t_\ell )$, ($s^{\scriptscriptstyle (\ell )}_i<s^{\scriptscriptstyle (\ell )}_{i+1}$) and ${k}^{\scriptscriptstyle (\ell )}=(k_1^{\scriptscriptstyle (\ell )},\dots ,k^{\scriptscriptstyle (\ell )}_{m^{\scriptscriptstyle (\ell )}})$) as in Remark 2.3,

$$\begin{aligned} E_{\mathbb {N}_{\scriptscriptstyle H}^{1,\sigma ^2}}\left[ \prod _{\ell =1}^r H_{t_\ell }(\phi ^{\scriptscriptstyle (\ell )})\right] = \sum _{{\digamma }\in \Sigma _r}\int _{{u}\in \mathcal {M}({t},{\digamma })} \Phi _{\sigma ^2}({\digamma },{u},{\varvec{s}}, {\varvec{k}}) d{u}. \end{aligned}$$

The following result is proved in Sect. 2.4 below.

#### Theorem 2.7

Let $d>8$. There exists $L_0$ such that for all $L\ge L_0$, and $r\in \mathbb {N}$, ${t}$, and $\phi ^{\scriptscriptstyle (1)},\dots , \phi ^{\scriptscriptstyle (r)}\in \mathcal {G}^*$ as in Proposition 2.6,

2.7 $$\begin{aligned} E_{\mu _{n}^{\scriptscriptstyle {\textrm{LT}}}}\left[ \prod _{\ell =1}^r H^{\scriptscriptstyle (n)}_{t_\ell }(\phi ^{\scriptscriptstyle (\ell )})\right] \rightarrow \sum _{{\digamma }\in \Sigma _r}\int _{{u}\in \mathcal {M}({t},{\digamma })} \Phi _{\sigma _0^2}({\digamma },{u},{\varvec{s}}, {\varvec{k}}) d{u}\quad \text {as}\, n \rightarrow \infty . \end{aligned}$$

#### Proof of Proposition 2.4

As noted after the statement of the Proposition, we only need verify condition (2.1) in Theorem 2.1 with $\mathcal {G}=\mathcal {G}^*$, and this is immediate from Proposition 2.6 and Theorem 2.7. $\square$

### Branching Brownian motion f.d.d. and proof of Proposition 2.6

#### Definition 2.8

Let $r\in \mathbb {N}$, ${\digamma }\in \Sigma _r$, ${t}\in (0,\infty )^r$, and ${u}\in \mathcal {M}({t},{\digamma })$. For each edge *e* we let $\ell (e)\in \{1,\dots ,r\}$ be the minimal leaf such that $e\in \mathcal {E}_\ell ({\digamma })$. Let $(W^i_s)_{s\le t_i}$ for $i\in [r]$ be (dependent) *d*-dimensional Brownian motions with variance parameter $\sigma ^2$, such that for any distinct $i,j\in \{1,\dots ,r\}$,

2.8 $$\begin{aligned} W^i_s=W^j_s \quad \text { for all } s\le u_{i\wedge j}, \end{aligned}$$

(recall $u_{i\wedge j}$ is the distance from the root to the gca of *i* and *j*) and

2.9 $$\begin{aligned}&\big \{(W^{\ell (e)}_{u_-(e)+s}-W^{\ell (e)}_{u_-(e)})_{s\le u_+(e)-u_-(e)}:e \text { an edge of }\mathbb {T}({\digamma },{u})\big \}\nonumber \\&\quad \text { are independent}\,d\text {-dimensional Brownian motions with variance }\sigma ^2. \end{aligned}$$

We call $(W^1,\dots ,W^r)$ a *tree-indexed BM* with variance parameter $\sigma ^2$ on $\mathbb {T}({\digamma },{u})$. $\blacktriangleleft$

(2.9) simply says that the collection of Brownian motions run along the disjoint edges of $\mathbb {T}({\digamma },{u})$ are independent. Note that in (2.9) we could choose any $\ell$ such that $e\in \mathcal {E}_\ell ({\digamma })$ by (2.8). We remark that the law of $(W^1,\dots ,W^r)$ is uniquely specified by the above (note it is mean zero Gaussian with $\text {Cov}(W^i(s_i),W^j(s_j))=\sigma ^2\min (u_{i\wedge j}, s_i, s_j)$).

#### Proposition 2.9

Let $r\in \mathbb {N}$, ${\digamma }\in \Sigma _r$, ${t}\in (0,\infty )^r$, ${u}\in \mathcal {M}({t},{\digamma })$, and $(W^i_s)_{s\le t_i}$ for $i \in [r]$ be a tree-indexed BM with variance parameter $\sigma ^2$ on $\mathbb {T}({\digamma },{u})$. If $\ell \in [r]$, $m^{\scriptscriptstyle (\ell )}\in \mathbb {N}$, ${s}^{\scriptscriptstyle (\ell )}=(0=s^{\scriptscriptstyle (\ell )}_0,s^{\scriptscriptstyle (\ell )}_1,\dots , s^{\scriptscriptstyle (\ell )}_{m^{\scriptscriptstyle (\ell )}}=t_\ell )$, ($s^{\scriptscriptstyle (\ell )}_i<s^{\scriptscriptstyle (\ell )}_{i+1}$), ${k}^{\scriptscriptstyle (\ell )}\in \mathbb {R}^{m(\ell )}$, and $\phi ^{\scriptscriptstyle (\ell )}=\phi _{{s}^{\scriptscriptstyle (\ell )},{k}^{\scriptscriptstyle (\ell )}}$, then

$$\begin{aligned} \mathbb {E}\Big [\prod _{\ell =1}^r \phi ^{\scriptscriptstyle (\ell )}(W^\ell )\Big ]=\Phi _{\sigma ^2}({\digamma },{u},{\varvec{s}}, {\varvec{k}}). \end{aligned}$$

#### Proof

This is an elementary calculation which divides the dependent Brownian increments on the left-hand side into smaller non-overlapping independent increments and keeps track of the Fourier coefficients multiplying each increment. The details are left for the reader. $\square$

**Notation.** For $t\ge 0$, let

2.10 $$\begin{aligned} {[}t]_n=\max \{k/n\in [0,t]: k\in \mathbb {Z}_+\}. \end{aligned}$$

#### Proof of Proposition 2.6

We will work with the measures $\mu _{n}^{\scriptscriptstyle {\textrm{BBM}}}$ for branching Brownian motion where the variance parameter is $\sigma ^2>0$ and the offspring distribution is critical binary branching, i.e., $\frac{1}{2}\delta _0+\frac{1}{2}\delta _2$, and so $\gamma =1$. In this case, [23, Proposition 2.6(a)(i)] with $\phi =1$, and Doob’s strong $L^p$ inequality for martingales imply

2.11 $$\begin{aligned} \forall p>1\ \ \text { there is a }C_p \text { such that }\forall K\in \mathbb {N}\ \ \sup _{n}E_{\mu _{n}}\Big [\sup _{t\le K}H^{\scriptscriptstyle (n)}_t(1)^p\Big ]\le C_pK^{p-1}. \end{aligned}$$

Theorem 1.3 and the continuity of $t\rightarrow H_t$ under $\mathbb {N}_{\scriptscriptstyle H}=\mathbb {N}^{1,\sigma ^2}_{\scriptscriptstyle H}$ imply weak convergence of $(H^{\scriptscriptstyle (n)}_{t_1}(\phi ^{\scriptscriptstyle (1)}),\dots , H^{\scriptscriptstyle (n)}_{t_r}(\phi ^{\scriptscriptstyle (r)}))$ under $(\mu _n^{\scriptscriptstyle {\textrm{BBM}}})^{t_1}$ to $(H_{t_1}(\phi ^{\scriptscriptstyle (1)}),\dots , H_{t_r}(\phi ^{\scriptscriptstyle (r)}))$ under $\mathbb {N}_H^{t_1}$ (see, e.g., [8, Theorem 10.2 in Ch. 3]). Note also that for *K* large enough, $\big |\prod _{l=1}^r H^{\scriptscriptstyle (n)}_{t_\ell }(\phi ^{\scriptscriptstyle (\ell )})\big |^2\le \sup _{t\le K}H_t^{\scriptscriptstyle (n)}(1)^{2r}$. Therefore, the above together with (2.11) and Dominated Convergence imply that

2.12 $$\begin{aligned} E_{\mathbb {N}_{\scriptscriptstyle H}} \left[ \prod _{\ell =1}^r H_{t_\ell }(\phi ^{\scriptscriptstyle (\ell )})\right]&= E_{\mathbb {N}^{t_1}_{\scriptscriptstyle H}} \left[ \prod _{\ell =1}^r H_{t_\ell }(\phi ^{\scriptscriptstyle (\ell )})\right] \mathbb {N}_{\scriptscriptstyle H}(S>t_1)\nonumber \\&=\lim _{n\rightarrow \infty }E_{(\mu _{n}^{\scriptscriptstyle {\textrm{BBM}}})^{t_1}}\Bigg [\prod _{\ell =1}^rH^{\scriptscriptstyle (n)}_{t_\ell }(\phi ^{\scriptscriptstyle (\ell )})\Bigg ]\mu _{n}^{\scriptscriptstyle {\textrm{BBM}}}(S^{\scriptscriptstyle (n)}>t_1)\nonumber \\&=\lim _{n\rightarrow \infty }E_{\mu _{n}^{\scriptscriptstyle {\textrm{BBM}}}}\Bigg [\prod _{\ell =1}^rH^{\scriptscriptstyle (n)}_{t_\ell }(\phi ^{\scriptscriptstyle (\ell )})\Bigg ]. \end{aligned}$$

A moment calculation for branching Brownian motion which uses Proposition 2.9 and is much simpler than that for lattice trees in Theorem 2.7, shows that the limit on the right-hand side of the above equals the right-hand side of the equality in the proposition. We sketch the proof as it explains how the right-hand side of (2.7) arises. Let $\mathbb {Z}_{+}/n=\{j/n:j\in \mathbb {Z}_+\}$. Recall (1.10), and let $I_t=\{\beta \in I:|\beta |=\lfloor t\rfloor \}$. Fix $t_1,\dots ,t_r>0$ and consider only large enough *n* so that

$$\begin{aligned} \lfloor nt_i\rfloor \ge 2,\ \ i=1,\dots ,r. \end{aligned}$$

Recall the random subset *GW* of indices in *I* defined in Sect. 1.2.1. A simple expansion of the sum defining $H_{t_\ell }^{\scriptscriptstyle (n)}$ shows that

**Fig. 7:**
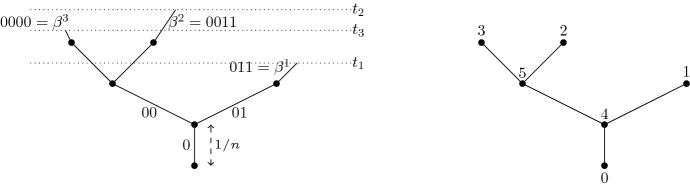
On the left is (part of) a GW tree with $\beta ^1,\beta ^2,\beta ^3$ indicated. Here $|\beta ^1|=|\beta ^5|=2$, $|\beta ^2|=|\beta ^3|=3$, and $|\beta ^4|=1$, and this contributes to (2.13) when $t_2,t_3\in [3/n,4/n)$ and $t_1\in [2/n,3/n)$ as depicted. On the right is the corresponding tree shape. The edge lengths associated to the latter are determined by taking differences of the $u_e$, where $u_4=1/n$, $u_5=2/n$, $u_1=t_1$, $u_2=t_2$, $u_3=t_3$

2.13 $$\begin{aligned} E_{\mu _{n}^{\scriptscriptstyle {\textrm{BBM}}}}\Bigg [\prod _{\ell =1}^rH^{\scriptscriptstyle (n)}_{t_\ell }(\phi ^{\scriptscriptstyle (\ell )})\Bigg ]&=\frac{1}{n^{r-1}}\sum _{\beta ^1\in I_{nt_1}}\dots \sum _{\beta ^r\in I_{nt_r}} \mathbb {E}\Bigg [\mathbb {1}_{\{\{\beta ^1,\dots ,\beta ^r\}\subset GW\}}\prod _{\ell =1}^r\phi ^{\scriptscriptstyle (\ell )}(B^{\beta ^\ell }_{\cdot \wedge t_{\ell }})\Bigg ]\nonumber \\&=\frac{1}{n^{r-1}}\sum _{\beta ^1\in I_{nt_1}}\dots \sum _{\beta ^r\in I_{nt_r}}\mathbb {P}\big (\{\beta ^1,\dots ,\beta ^r\}\subset GW\big )\mathbb {E}\Big [\prod _{\ell =1}^r\phi ^{\scriptscriptstyle (\ell )}({\hat{B}}^{\beta ^\ell }_{\cdot \wedge t_{\ell }})\Big ], \end{aligned}$$

where in the last we used the independence of the branching variables $\{e^\beta :\beta \in I\}$ and the spatial motions $\{\hat{B}^\beta :\beta \in I\}$ as well as the fact that $B^{\beta ^\ell }_{\cdot \wedge t_\ell }=\hat{B}^{\beta ^\ell }_{\cdot \wedge t_\ell }$ if $\beta ^\ell \in GW$. It is easy to see that the contribution to the above sum from $\beta ^1,\dots ,\beta ^r$ such that for some $i\ne j$: $\pi \beta ^i$ is an ancestor of $\beta ^j$, is bounded by *C*(*r*, *K*)/*n* for $\max \{t_i:i \in [r]\}\le K$. To see this, note that if $\pi \beta ^i$ is an ancestor of $\beta ^j$, then $\pi \beta ^i$ is determined by $\beta ^j$ since its length is $\lfloor nt_i\rfloor -1$. This means there are only two possible values of $\beta ^i$ and so we can bound this contribution by twice the $(r-1)$-fold sum with each ${k}^{\scriptscriptstyle (\ell )}={0}$ (so each $\phi ^{\scriptscriptstyle (\ell )}=1$), and applying (2.11), we obtain the above bound. Fix ${\beta }:=(\beta ^1,\dots ,\beta ^r)\in I_{nt_1}\times \dots \times I_{nt_r}$ so that none of the indices has a parent which is an ancestor of another index (in particular all are distinct). Call such a ${\beta }$ a good value of ${\beta }$. Then, in particular, ${\beta }$ uniquely determines a non-degenerate shape ${\digamma }({\beta })\in \Sigma _r$ where $\beta ^1,\dots , \beta ^r$ label the *r* leaves and one can define the internal vertices of the shape by locating the branch points from the root to $\beta ^1$, then the new branch points while proceeding from the root to $\beta ^2$, and so on up to $\beta ^r$. See e.g. Fig. 7. In this way we label the internal vertices by $\beta ^{r+1},\dots ,\beta ^{2r-1}$ using our labelling convention in Definition 2.5 (now with $\beta ^i$ in place of *i*). For example (assuming $r>1$), $\beta ^{r+1}=\beta ^1|\kappa _{r+1}$, where $\kappa _{r+1}=\max \{\kappa :\beta ^1|\kappa =\beta ^\ell |\kappa \text { for all }\ell >1\}\in \{0,\dots ,\min \{|\beta ^\ell |\}-2\}$ (the upper bound since ${\beta }$ is good), and then continue down the branch towards $\beta ^1$ until there is only one leaf ($\beta ^1$) along the remaining tree. Note that each $\beta ^\ell$ for $\ell >r$ is of the form $\beta ^i|\kappa _\ell$ for some $i=i(\ell )\le r$ and some $\kappa _\ell <|\beta ^i|$, i.e. is an ancestor of some $\beta ^i$.

We introduce tree distances ${u}({\beta })=(u_1,\dots ,u_{2r-1})$ for the above shape, with ${u_i\in \mathbb {Z}_{+}/n\setminus \{0\}}$ for $i>r$, by setting

$$\begin{aligned} u_\ell ={\left\{ \begin{array}{ll} t_\ell &{} \text { if }\ell \le r,\\ (|\beta ^\ell |+1)/n&{} \text { if }\ell \in \{r+1,\dots ,2r-1\}.\end{array}\right. } \end{aligned}$$

Recall that $u_\ell$ is the distance from vertex $\beta ^\ell$ to the root and so edge distances can be found by differencing. Denote this tree shape with edge lengths by $\mathbb {T}({\beta })$. Note that the fact that ${\beta }$ is good ensures that $u_\ell <|\beta ^i|/n \le u_i$, whenever $\beta ^\ell$ is an ancestor of $\beta ^i$ for $\ell >r$ and $i\le r$. In fact, the possible values of ${u}$ are now given by the discrete analogue of $\mathcal {M}({t},{\digamma })$,

2.14 $$\begin{aligned} {u}\in \mathcal {M}_n({t},{\digamma })&:=\{{u} \in (0,\infty )^{2r-1}:\, u_i=t_i \text { for }i\le r, u_i\in \mathbb {Z}_{+}/n\setminus \{0\} \text { for }i>r\nonumber \\&\qquad \qquad \qquad \text { and }u_k<u_j\text { whenever }\beta ^k \text { is an ancestor of }\beta ^j\}. \end{aligned}$$

In the above notation we use the fact that the ordering of the leaves given by ${t}$, the shape ${\digamma }$, and our convention on numbering internal vertices, determines the ancestral relationship between the ${\beta ^k}$, not the particular choice of ${\beta }$. The definition of $u_\ell$ for the internal branch points $\ell >r$ ensures that

2.15 $$\begin{aligned} (W^1,\dots ,W^r):=\,&(\hat{B}^{\beta ^1},\dots , \hat{B}^{\beta ^r})\text { is a tree-indexed Brownian motion}\nonumber \\&\quad \text {with variance parameter}\, \sigma ^2\,\text { on }\,\mathbb {T}({\beta }). \end{aligned}$$

To see this, note that at a branch point $\beta ^\ell =\beta ^i\wedge \beta ^j$ for leaves *i*, *j* and $\ell >r$, the Brownian paths $\hat{B}^{\beta ^i}$ and $\hat{B}^{\beta ^j}$ do not split apart and evolve independently until time $(|\beta ^\ell |+1)/n=u_\ell$.

We now decompose the sum over good ${\beta }$ in (2.13) according to its shape, ${\digamma }$, and edge lengths ${u}$. Abbreviating $(\beta _1,\dots ,\beta _r)\in I_{nt_1}\times \dots \times I_{nt_r}$ as ${\beta }\in I_{n{t}}$, and writing ${\beta }\subset GW$ for $\{\beta ^1,\dots , \beta ^r\}\subset GW$, the right hand side of (2.13) becomes

2.16 $$\begin{aligned} \sum _{{\digamma }\in \Sigma _r}\frac{1}{n^{r-1}}\sum _{{u}\in \mathcal {M}_n({t},{\digamma })}\ \ \sum _{\begin{array}{c} {\beta }\in I_{n{t}}:\\ \beta \text { good} \end{array}}&\mathbb {1}_{\{{\digamma }({\beta })={\digamma }\}}\mathbb {1}_{\{{u}({\beta })={u}\}} \mathbb {P}({\beta }\subset GW) \mathbb {E}\Big [\prod _{\ell =1}^r\phi ^{\scriptscriptstyle (\ell )}({\hat{B}}^{\beta ^\ell }_{\cdot \wedge t_{\ell }})\Big ]+\mathcal {O}\Big (\frac{1}{n}\Big ). \end{aligned}$$

Recall the notation (2.10). Choose ${\digamma }\in \Sigma _r$, ${u}\in \mathcal {M}_n({t},{\digamma })$, and ${\beta }\in I_{n{t}}$ such that ${\digamma }({\beta })={\digamma }$ and ${u}({\beta })={u}$. Let $N=N({\digamma },{u})\in \mathbb {Z}_+$ be the number of ancestors of $\beta ^1,\dots , \beta ^r$ in the index set *I*. Note that *N* is equal to *n* times the sum of (truncated) edge lengths in $\mathbb {T}({\beta })$ determined by ${u}'$ where $u'_\ell =u_\ell$ if $\ell >r$ and $u'_\ell =[u_{\ell }]_n=[t_\ell ]_n$ if $\ell \le r$ (see e.g. the left hand side of Fig. 7). (Here we identify each edge of rescaled length 1/*n* with the index of its entry vertex in *I*.) Therefore *N* is a function of $({\digamma },{u})$ as the notation suggests. It follows immediately that $\mathbb {P}({\beta }\subset GW)=2^{-N}$ since ${\beta }\subset GW$ if and only if each of these ancestors has two offspring.

It follows from this, (2.15), and Proposition 2.9, that (2.16) equals

$$\begin{aligned}&\sum _{{\digamma }\in \Sigma _r}\frac{1}{n^{r-1}}\sum _{{u}\in \mathcal {M}_n({t},{\digamma })}\ \ \sum _{{\beta }\in I_{n {t}}, \beta \text { good}} \mathbb {1}_{\{{\digamma }({\beta })={\digamma }\}}\mathbb {1}_{\{{u}({\beta })={u}\}} \mathbb {P}({\beta }\subset GW) \Phi ({\digamma },{u},{\varvec{s}}, {\varvec{k}})+\mathcal {O}\Big (\frac{1}{n}\Big )\\&\quad =\sum _{{\digamma }\in \Sigma _r}\frac{1}{n^{r-1}}\sum _{{u}\in \mathcal {M}_n({t},{\digamma })}2^{-N}\Phi ({\digamma },{u},{\varvec{s}}, {\varvec{k}})\sum _{{\beta }\in I_{n {t}}} \mathbb {1}_{\{{\digamma }({\beta })={\digamma }\}}\mathbb {1}_{\{{u}({\beta })={u}\}}+\mathcal {O}\Big (\frac{1}{n}\Big ). \end{aligned}$$

Here dropping the “good” requirement on $\beta$, at the cost of $\mathcal {O}\Big (\frac{1}{n}\Big )$, is again an easy calculation along the lines of that done earlier.

For fixed ${\digamma }\in \Sigma _r$ and ${u}\in \mathcal {M}_n({t},{\digamma })$, the number of choices for ${\beta }\subset I$ with this shape and edge lengths in the above is $2^{N}$. This is because there are two choices for the offspring labels for each of the *N* “ancestors” above. Therefore combining the above equalities leads to

$$\begin{aligned} E_{\mu _{n}^{\scriptscriptstyle {\textrm{BBM}}}}&\Bigg [\prod _{\ell =1}^rH^{\scriptscriptstyle (n)}_{t_\ell }(\phi ^{\scriptscriptstyle (\ell )})\Bigg ] =\sum _{{\digamma }\in \Sigma _r}\frac{1}{n^{r-1}}\sum _{{u}\in \mathcal {M}_n({t},{\digamma })}\Phi ({\digamma },{u},{\varvec{s}}, {\varvec{k}})+\mathcal {O}\Big (\frac{1}{n}\Big ). \end{aligned}$$

As $n\rightarrow \infty$ in the above, the $(r-1)$-fold Riemann sum converges to the $(r-1)$-dimensional integral in the right-hand side of the proposition, and so the result now follows from (2.12). For the Riemann sum convergence, we note that the ${u}$ dependence of the integrand admits finitely many jump discontinuities. $\square$

### Lattice tree f.d.d

We now turn to the LT setting. Fix $m\in \mathbb {N}$, $t>0$, ${k}=(k_1,\dots , k_m)\in \mathbb {R}^{dm}$, and ${s}=(0=s_0,\dots , s_m=t)$, where $s_i<s_{i+1}$. Then

2.17 $$\begin{aligned} H^{\scriptscriptstyle (n)}_{t}(\phi _{{s},{k}})&=\int _{\mathcal {D}}\phi dH^{\scriptscriptstyle (n)}_t=\frac{1}{C_0n} \sum _{\sqrt{n}x\in \mathcal {T}_{nt}} \prod _{j=1}^{m}{\textrm{e}}^{{\textrm{i}}k_{j}\big ( w^{\scriptscriptstyle (n)}_{s_j}(t,x)-w^{\scriptscriptstyle (n)}_{s_{j-1}}(t,x) \big )}. \end{aligned}$$

Letting ${x}_m=(x_1,\dots , x_m)$ and setting $x_0=0\in \mathbb {Z}^d$ we have

2.18 $$\begin{aligned} E_{\mu ^{\scriptscriptstyle {\textrm{LT}}}_{n}}[H_t^{\scriptscriptstyle (n)}(\phi _{{s},{k}})]=\frac{C_1}{C_0}\sum _{{x}_m \in (\mathbb {Z}^d)^m}\prod _{j=1}^m {\textrm{e}}^{{\textrm{i}}\frac{k_{j}}{\sqrt{n}}(x_j-x_{j-1})}\mathbb {P}\Big (x_m\in \mathcal {T}_{nt}, \cap _{j=1}^m\{ w_{ns_j}(nt,x_m) =x_j\}\Big ). \end{aligned}$$

**Fig. 8:**
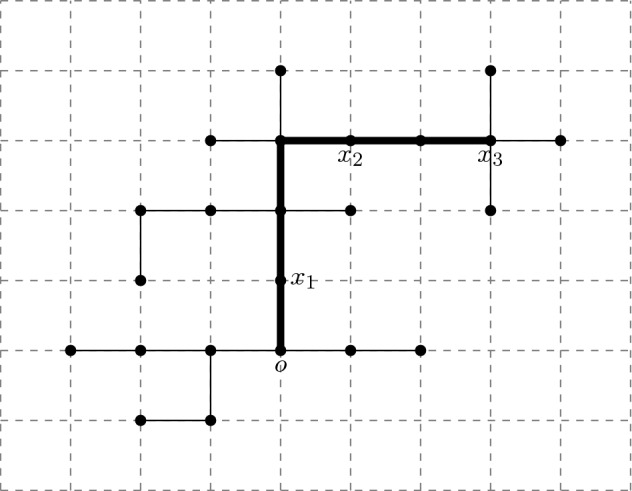
A depiction of the event in the detailed 1-particle function with $n=1$, $t=6$, $s_1=1$ and $s_2=4$, with the path $s\mapsto w_{s}(6,x_3)$ in bold (recall the notation from (1.1))

We call the quantity $\mathbb {P}\big (x_m\in \mathcal {T}_{nt},\cap _{j=1}^m\{ w_{ns_j}(nt,x_m) =x_j\}\big )$ a *detailed 1-particle function*, (see e.g. Fig. 8), and the Fourier transform of the increments is called a *detailed* 1-particle transform, i.e.

$$\begin{aligned} \sum _{{x}_m \in (\mathbb {Z}^d)^m}\prod _{j=1}^m {\textrm{e}}^{{\textrm{i}}k_{j}(x_j-x_{j-1})}\mathbb {P}\Big (x_m\in \mathcal {T}_{nt}, \cap _{j=1}^m\{ w_{ns_j}(nt,x_m) =x_j\}\Big ). \end{aligned}$$

Related quantities arising from expectations of the form

$$\begin{aligned} E_{\mu ^{\scriptscriptstyle {\textrm{LT}}}_{n}}\left[ \prod _{\ell =1}^{r} H^{\scriptscriptstyle (n)}_{t_\ell }(\phi ^{\scriptscriptstyle (\ell )})\right] , \end{aligned}$$

with $\phi ^{\scriptscriptstyle (\ell )}=\phi _{{s}^{\scriptscriptstyle (\ell )},{k}^{\scriptscriptstyle (\ell )}}$ $({s}^{\scriptscriptstyle (\ell )},{k}^{\scriptscriptstyle (\ell )}$ as in Theorem 2.7) are called *detailed* *r-particle transforms*. Therefore Theorem 2.7 amounts to verifying the appropriate asymptotics for the detailed *r*-particle transforms.

**Fig. 9:**
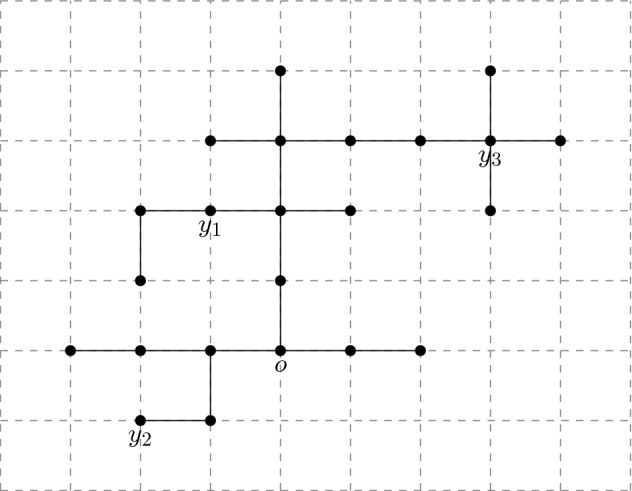
A depiction of the event in the 3-particle function $\rho _{(3,3,6)}(y_1,y_2,y_3)$

When $m=1$, the detailed 1-particle function is simply $\mathbb {P}(x_1\in \mathcal {T}_{nt})$, and its Fourier transform becomes $\sum _{x \in \mathbb {Z}^d}{\textrm{e}}^{{\textrm{i}}k_1 x}\mathbb {P}(x\in \mathcal {T}_{nt})$. These quantities are called the 1-particle functions (traditionally in the literature these have been called the 2-point functions, with the two points being the origin *o* and $x_1$). For ${n}\in \mathbb {Z}_+^{r}$ and ${x}=(x_1,\dots , x_{r})\in \mathbb {Z}^{dr}$ we can define the *r*-particle functions (see e.g. Fig. 9):

$$\begin{aligned} \rho _{{n}}({x})=\mathbb {P}(\cap _{i=1}^{r}\{x_i\in \mathcal {T}_{n_i}\}), \end{aligned}$$

and (their Fourier transforms) the *r*-particle transforms for ${k}\in (\mathbb {R}^d)^r$:

$$\begin{aligned} \hat{\rho }_{{n}}({k})=\sum _{{x}\in (\mathbb {Z}^d)^r}{\textrm{e}}^{{\textrm{i}}{k}\cdot {x}}\rho _{{n}}({x}). \end{aligned}$$

We write $\mathcal {O}(x)$ to denote a quantity whose absolute value is bounded by a constant times *x*. Using the inductive method of [12, 14] the following was shown in [17, Theorem 3.7]:

#### Theorem 2.10

[17]. Fix $d>8$. There exists $L_0 = L_0(d)\gg 1$ such that for every $L\ge L_0$:

There exist $K,C_A>0$ such that, for every $\delta \in (0, 1\wedge \frac{d-8}{2})$,

2.19 $$\begin{aligned} \sup _{n\in \mathbb {Z}_+}\sup _{k \in \mathbb {R}^d}|\hat{\rho }_n(k)|=\sup _{n\in \mathbb {Z}_+}\hat{\rho }_n(0)\le K, \end{aligned}$$

and

2.20 $$\begin{aligned} \hat{\rho }_n\left( \frac{k}{\sqrt{n}}\right) =C_A{\textrm{e}}^{-\frac{\sigma _0^2|k|^2}{2}}\left[ 1 + \mathcal {O}\left( \frac{|k|^2}{n^{\delta }}\right) +\mathcal {O}\left( n^{-\frac{d-8}{2}}\right) \right] . \end{aligned}$$

Recall that the constant $C_A$ is equal to $A'$ in the paper [17], while $\sigma _0^2$ is equal to $v\sigma ^2/d$ in [17]. The error terms (see [17, Theorem 3.7, Lemma 3.8]) in (2.20) depend on *d*, *L* but are uniform in $\{k \in \mathbb {R}^d:|k|^2 \le C \log n\}$ (where *C* depends on $\delta$). Taking $k=0$ above we see that, as claimed in Sect. 1.1, $C_A=\lim _{n \rightarrow \infty }\mathbb {E}[|\mathcal {T}_n|]$. Asymptotics for the *r*-particle transforms are provided in [17, Theorem 1.14]. In particular there exists $C_V>0$ depending on *D*, *d* such that

2.21 $$\begin{aligned} n^{-1}\mathbb {E}[|\mathcal {T}_n|^2]=n^{-1}\hat{\rho }_{(n,n)}(0,0)\rightarrow C_V C_A^3. \end{aligned}$$

Recall that the constant $C_V$ in our paper is equal to $V\rho ^2$ in [17]. Our task is to “upgrade” these kinds of results from [17] to get asymptotics for the “detailed” *r*-particle transforms. This is the focus of the next section.

### The LT detailed *r*-particle transforms and proof of Theorem 2.7

Recall the labelling convention for internal vertices (branch points) and edges in ${\digamma }$ from Definition 2.5.

A lattice tree $T\ni o$ having $r+1$ leaves ($o=x_0$ and $x_1,\dots , x_r$), $r-1$ vertices $x_{r+1}, \dots , x_{2r-1}$ of degree 3, and all other vertices degree 2, has an associated abstract tree $\Gamma$ as follows: $x_i \mapsto i$, and any two vertices $i,i'$ in $\Gamma$ are connected via a single edge if the shortest path from $x_i$ to $x_{i'}$ in *T* passes through no other $x_j$. All vertices in $\Gamma$ are degree 1 or 3. Relabelling the vertices of degree 3 according to the labelling convention in Definition 2.5 gives an abstract shape $\Gamma '$, which is the *shape of* *T* and the points $x_1,\dots , x_r$ (and *o*), and we write $v_g\in \{x_{r+1},\dots , x_{2r-1}\}$ for the vertex in *T* that mapped to branch point $g\in \Gamma '$.

Given ${\digamma }$, $\check{\varvec{y}}=(\check{y}_{e,i})_{i \in [j_e],e\in [2r-1]}$, and $\check{\varvec{n}}=(\check{n}_{e,i} )_{i \in [j_e],e\in [2r-1]}$ with each $\check{y}_{e,i}\in \mathbb {Z}^d$ and each $\check{n}_{e,i}\in \mathbb {N}$, let $\check{\varvec{T}}({\digamma },\check{\varvec{y}},\check{\varvec{n}})$ denote the set of lattice trees $T\ni o$ such that:

(*): for each $\ell \in [r]$ the tree *T* contains $x_\ell =\sum _{e\in \mathcal {E}_\ell ({\digamma })}\sum _{i=1}^{j_e}\check{y}_{e,i}$, and the shape of the minimal subtree $T'$ of *T* containing *o* and $x_1,\dots , x_r$ is ${\digamma }$, and for each branch point $g \in {\digamma }$, the corresponding vertex $v_g$ is tree distance $\sum _{f\prec g} \sum _{i=1}^{j_f}\check{n}_{f,i}+\sum _{i=1}^{j_g}\check{n}_{g,i}$ from the root in $T'$, and
(**): for each $\ell \in [r]$, each $e\in \mathcal {E}_\ell ({\digamma })$, and each $i_e\in \{1,\dots ,j_e\}$, the path from *o* to $x_\ell$ in *T* passes through the point $\sum _{f\prec e}\sum _{i=1}^{j_f}\check{y}_{f,i}+\sum _{i=1}^{i_e}\check{y}_{e,i}\in \mathbb {Z}^d$ at time (tree distance from the root) $\sum _{f\prec e}\sum _{i=1}^{j_f}\check{n}_{f,i}+\sum _{i=1}^{i_e}\check{n}_{e,i}\in \mathbb {Z}^d$.

Let

2.22 $$\begin{aligned} t^{({\digamma })}_{\check{\varvec{n}}}(\check{\varvec{y}})&=\rho \mathbb {P}\Big (\mathcal {T}\in \check{\varvec{T}}({\digamma },\check{\varvec{y}},\check{\varvec{n}})\Big ). \end{aligned}$$

Given *n*, ${\digamma }$, and $\check{\varvec{n}}$ as above, and $\check{\varvec{k}}=(\check{k}_{e,i} )_{i \in [j_e],e\in [2r-1]}$ with each $\check{k}_{e,i}\in \mathbb {R}^d$, define

$$\begin{aligned} \hat{t}^{({\digamma })}_{\check{\varvec{n}}}(\check{\varvec{k}})= \sum _{\check{\varvec{y}}} \prod _{e=1}^{2r-1}\prod _{i=1}^{j_e}{\textrm{e}}^{{\textrm{i}}\check{k}_{e,i}\cdot \check{y}_{e,i}} {t^{({\digamma })}_{\check{\varvec{n}}}(\check{\varvec{y}})}. \end{aligned}$$

The following proposition will be proved in Sect. 4.5 via modifications of [17, Theorem 4.8] (where each $j_e=1$) as indicated in [18]:

#### Proposition 2.11

Fix $d>8$. There exists $L_0(d)$ such that for every $L\ge L_0$: for every $\delta \in (0, 1\wedge \frac{d-8}{2})$, $\varepsilon >0$, $r\in \mathbb {N}$, ${\digamma }\in \Sigma _r$, $(j_e)_{e\in [2r-1]}\in \mathbb {N}^{2r-1}$, $\check{\varvec{n}}=(\check{n}_{e,i})_{i\in [j_e],e\in [2d-1]}$ (with each $\check{n}_{e,i}\in \mathbb {N}$ and each $\check{n}_{e,i}/n \in (\varepsilon ,1/\varepsilon )$), $R>0$, $\check{\varvec{k}}=(\check{k}_{e,i})_{i\in [j_e],e\in [2d-1]}$ (with each $\check{k}_{e,i}\in [-R,R]^d$),

$$\begin{aligned} \hat{t}^{({\digamma })}_{\check{\varvec{n}}}\Bigl (\frac{\check{\varvec{k}}}{\sqrt{ n}}\Bigr )&=\rho C_V^{r-1}C_A^{2r-1} \prod _{e=1}^{2r-1} \prod _{i=1}^{j_e}{\textrm{e}}^{-\sigma _0^2\frac{\check{k}_{e,i}^2}{2}\frac{\check{n}_{e,i}}{n}}\\&\quad + \mathcal {O}\Bigl (\sum _{e=1}^{2r-1}\sum _{i=1}^{j_e} \frac{1}{\check{n}_{e,i}^{\frac{d-8}{2}}}\Bigr ) +\mathcal {O}\Bigl (\sum _{e=1}^{2r-1}\sum _{i=1}^{j_e}\frac{\left| \check{\varvec{k}}\right| ^2\check{n}_{e,i}^{1-\delta }}{n}\Bigr ), \end{aligned}$$

where the constants in the error terms depend on *L*, $\delta$, *r*, *R*, $(j_e)_{e\in [2r-1]}$ and $\varepsilon >0$.

The purpose of this section is to prove Theorem 2.7 using Proposition 2.11.

We begin with generalisations of (2.17) and (2.18) (where $r=1$). Fix $r \ge 1$ and $t_1,\dots , t_r>0$. Let ${\varvec{s}}=({s}^{\scriptscriptstyle (1)},\dots ,{s}^{\scriptscriptstyle (r)})$, where ${s}^{\scriptscriptstyle (\ell )}=(s_0^{\scriptscriptstyle (\ell )},\dots ,s^{\scriptscriptstyle (\ell )}_{m^{\scriptscriptstyle (\ell )}})$, and $0=s_0^{\scriptscriptstyle (\ell )}<s_1^{\scriptscriptstyle (\ell )}<\dots <s_{m^{\scriptscriptstyle (\ell )}}^{\scriptscriptstyle (\ell )}=t_\ell$ for each $\ell$ (so each $m^{\scriptscriptstyle (\ell )}\in \mathbb {N}$). Then for $\phi ^{\scriptscriptstyle (1)},\dots , \phi ^{\scriptscriptstyle (r)} \in \mathcal {G}$ (with $\phi ^{\scriptscriptstyle (\ell )}=\phi _{{s}^{\scriptscriptstyle (\ell )},{k}^{\scriptscriptstyle (\ell )}}$ and ${k}^{\scriptscriptstyle (\ell )}=(k_1^{\scriptscriptstyle (\ell )},\dots ,k^{\scriptscriptstyle (\ell )}_{m^{\scriptscriptstyle (\ell )}})\in (\mathbb {R}^d)^{m^{\scriptscriptstyle (\ell )}}$),

$$\begin{aligned}&\prod _{\ell =1}^r H^{\scriptscriptstyle (n)}_{t_\ell }(\phi ^{\scriptscriptstyle (\ell )})\\&\quad =\left( \frac{1}{C_0n}\right) ^r \sum _{\sqrt{n}x_1\in \mathcal {T}_{ nt_1}}\cdots \sum _{\sqrt{n}x_r\in \mathcal {T}_{nt_r}}\prod _{\ell =1}^r \prod _{j_\ell =1}^{m^{\scriptscriptstyle (\ell )}}\exp \Bigg \{{\textrm{i}}k_{j_\ell }^{\scriptscriptstyle (\ell )}\big (w^{\scriptscriptstyle (n)}_{s_{j_\ell }^{\scriptscriptstyle (\ell )}}(t_\ell ,x_\ell )-w^{\scriptscriptstyle (n)}_{s^{\scriptscriptstyle (\ell )}_{j_\ell -1}}(t_\ell ,x_\ell )\big )\Bigg \}. \end{aligned}$$

Take expectations and work with the un-normalised functions $w_{}(t,x)=w_{}(t,x)\big (T\big )$ (a slight abuse of notation, as before $w_{}(t,x)$ was defined as a function of the random tree $\mathcal {T}$) to see that

2.23 $$\begin{aligned} E_{\mu _n^{\scriptscriptstyle {\textrm{LT}}}}\left[ \prod _{\ell =1}^r H^{\scriptscriptstyle (n)}_{t_\ell }(\phi ^{\scriptscriptstyle (\ell )})\right]&=\frac{C_1}{C_0^rn^{r-1}} \sum _{{x} \in (\mathbb {Z}^d)^r} \sum _{\begin{array}{c} T\ni o:\\ {x}\in T_{n{t}} \end{array}}\mathbb {P}(\mathcal {T}=T)\nonumber \\&\quad \times \prod _{\ell =1}^r \prod _{j_\ell =1}^{m^{\scriptscriptstyle (\ell )}}\exp \Bigg \{{\textrm{i}}\frac{k_{j_\ell }^{\scriptscriptstyle (\ell )}}{\sqrt{n}}\big ( w_{ns_{j_\ell }^{\scriptscriptstyle (\ell )}}(nt_\ell ,x_\ell ) -w_{ns_{j_\ell -1}^{\scriptscriptstyle (\ell )}}(nt_\ell ,x_\ell ) \big )\Bigg \}, \end{aligned}$$

where ${x}\in T_{n{t}}$ means $x_i\in T_{nt_i}$ for each $i \in [r]$.

Given ${x}=(x_1,\dots ,x_r)\in (\mathbb {Z}^d)^r$ and $T\ni o$ a lattice tree with $x_1,\dots , x_r\in T$, one can consider the minimal subtree containing the origin and these points. Typically this subtree has $r-1$ branch points that are connected to the root and the points $x_i$ according to an abstract (rooted) shape ${\digamma }$ consisting of $2r-1$ edges $e\in \mathcal {E}({\digamma })$ and 2*r* vertices. Call this the shape associated to $(T,{x})$. Contributions from subtrees containing fewer than $r-1$ branch points (arising if (i) the number of distinct elements in $\{x_1,\dots , x_r\}$ is smaller than *r*, or (ii) paths in *T* to one or more $x_i$ contain paths to one or more other $x_j$, or (iii) the most recent common ancestor of two $x_j$’s is the origin, or (iv) some branch point in the subtree has degree more than 3) will constitute error terms (see e.g. (2.26) below) and they will be said to have a *degenerate* shape. For a given (non-degenerate) shape ${\digamma }\in \Sigma _r$, and ${t}=(t_1,\dots ,t_r)\in (\mathbb {R}_{>0})^r$, recall the definition of $\mathcal {M}_n({t},{\digamma })$ from (2.14) (but now with $\ell$ in place of $\beta ^\ell$). For ${x}\in (\mathbb {Z}^d)^r$, ${y}=(y_{r+1},\dots ,y_{2r-1})\in (\mathbb {Z}^d)^{r-1}$, and ${u}\in \mathcal {M}_n({t},{\digamma })$, let $\varvec{T}_n({\digamma },{t},{u},{x},{y})$ denote the set of lattice trees *T* containing the origin and the points $x_i\in T_{\lfloor nt_i \rfloor }$ for $i \in [r]$ for which the shape associated to $(T,{x})$ is ${\digamma }$, such that for each branch point $j=r+1,\dots ,2r-1$ in ${\digamma }$, the spatial and temporal location of the corresponding branch point in *T* is $(y_j,nu_j)$. The main contribution to (2.23) is therefore

2.24 $$\begin{aligned}&\frac{C_1\rho ^{-1}}{C_0^rn^{r-1}}\sum _{{\digamma }\in \Sigma _r}\sum _{\begin{array}{c} {u}\in \\ \mathcal {M}_n({t},{\digamma }) \end{array}}\sum _{\begin{array}{c} ({x},{y})\in \\ (\mathbb {Z}^d)^{2r-1} \end{array}} \sum _{\begin{array}{c} T \in \\ \varvec{T}_n({\digamma },{t},{u},{x},{y}) \end{array}} W(T)\nonumber \\&\quad \times \prod _{\ell =1}^r \prod _{j_\ell =1}^{m^{\scriptscriptstyle (\ell )}}\exp \Bigg \{{\textrm{i}}\frac{k_{j_\ell }^{\scriptscriptstyle (\ell )}}{\sqrt{n}}\big ( w_{ns_{j_\ell }^{\scriptscriptstyle (\ell )}}(nt_\ell ,x_\ell ) -w_{ns_{j_\ell -1}^{\scriptscriptstyle (\ell )}}(nt_\ell ,x_\ell ) \big )\Bigg \}. \end{aligned}$$

The modulus of each exponential is bounded by 1. Next, using (2.19), and neglecting interaction between parts of the tree corresponding to the $2r-1$ different edges in the shape we get that for any shape ${\digamma }\in \Sigma _r$,

2.25 $$\begin{aligned} \sum _{({x},{y})}\sum _{\begin{array}{c} T \in \\ \varvec{T}_n({\digamma },{t},{u},{x},{y}) \end{array}} W(T)\le K_0^{2r-1}, \end{aligned}$$

for some $K_0>0$. If $\bar{t}=\max _{i\in [r]}t_i$, we can sum over ${u}$ to conclude that

$$\begin{aligned} \sum _{\begin{array}{c} {u}\in \\ \mathcal {M}_n({t},{\digamma }) \end{array}}\sum _{\begin{array}{c} ({x},{y}) \end{array}} \sum _{\begin{array}{c} T \in \\ \varvec{T}_n({\digamma },{t},{u},{x},{y}) \end{array}}  W(T)\le K_0^{2r-1}(n\bar{t}+1)^{r-1}. \end{aligned}$$

#### Remark 2.12

Bounds similar to (2.25) hold in great generality. For any abstract rooted tree graph (call it a generalised shape) ${\digamma }^*$ with edge set $E^*$, and any set of temporal lengths $(n_e)_{e \in E^*}$ (with each $n_e\in \mathbb {N}$) associated with those edges: the total weight of all lattice trees containing the origin having vertices with spatial and temporal displacements $({\Delta }_e)_{e \in E^*}$ and $(n_e)_{e \in E^*}$ with the generalised shape of the connections to these points being ${\digamma }^*$, summed over $({\Delta }_e)_{e \in E^*}$ gives at most $K_0^{\#E^*}$. This is also obtained by ignoring interactions between different parts of the trees corresponding to different edges in $E^*$. $\bigstar$

For degenerate shapes, one also has (2.25) (in fact the exponent $2r-1$ can be reduced). However, in comparison with (2.24), degenerate shapes give rise to sums over fewer (at most $r-2$ in fact) $u_j$’s, each of which takes at most $n\bar{t}+1$ possible values. After summing over finitely many degenerate shapes and summing over ${u}$ we may bound the version of (2.24) for degenerate shapes by

2.26 $$\begin{aligned} \frac{C}{n^{r-1}}(n\bar{t}+1)^{r-2}\le C\frac{(\bar{t} +1)^{r-2}}{n}. \end{aligned}$$

We conclude that contributions to (2.23) from degenerate shapes are bounded in absolute value by $Cn^{-1}(\bar{t}+1)^{r-2}$ and the main contribution from non-degenerate shapes is at most $C(\bar{t}+1)^{r-1}$. If we set $m^{(\ell )}=1,{k}^{(\ell )}=0$, we conclude the following as a special case:

#### Lemma 2.13

For each $r\in \mathbb {N}$ there exists a constant $C_{r}>0$ such that for all $t_1,\dots , t_r{\ge }0$,

$$\begin{aligned} \sup _{n \in \mathbb {N}}n\mathbb {E}\Big [\prod _{i=1}^r H_{t_i}^{\scriptscriptstyle (n)}(1)\Big ]\le C_r(\bar{t}+ 1)^{r-1}.\end{aligned}$$

Given $\varepsilon >0$, ${t}$, $\varvec{s}$, and a (non-degenerate) shape ${\digamma }\in \Sigma _r$, let $\mathcal {M}_{n,\varepsilon }({t},{\digamma },\varvec{s})$ denote the set of ${u}\in \mathcal {M}_n({t},{\digamma })$ for which (with $u_0:=0$) either:

- there exist a leaf $\ell \in \{1,\dots ,r\}$, a branch point $j\in \{r+1,\dots ,2r-1\}$ in the path from *o* to $\ell$, and an $i\in \{1,\dots ,m^{\scriptscriptstyle (\ell )}\}$, such that
  $$\begin{aligned}|u_j-s^{\scriptscriptstyle (\ell )}_i|\le \varepsilon ,\end{aligned}$$
- there exist $i,j\in \{0,\dots ,2r-1\}$ vertices of ${\digamma }$, such that *i* is an ancestor of *j* in ${\digamma }$ and
  $$\begin{aligned}|u_i-u_j|\le \varepsilon .\end{aligned}$$

Roughly speaking these correspond to situations where there is branching on a path close to one of the observation times along the path, or where one of the edge-lengths is short.

Let $\mathcal {M}_{n,*}({t},{\digamma },\varvec{s})=\mathcal {M}_n({t},{\digamma })\setminus \mathcal {M}_{n,\varepsilon }({t},{\digamma },\varvec{s})$. Then the sum over ${u}$ in (2.24) can be split into a sum over ${u}\in \mathcal {M}_{n,*}({t},{\digamma },\varvec{s})$ and a sum over ${u}\in \mathcal {M}_{n,\varepsilon }({t},{\digamma },\varvec{s})$. Using the same argument as for (2.26), we get that the absolute value of the sum over ${u}\in \mathcal {M}_{n,\varepsilon }({t},{\digamma },\varvec{s})$ is at most

2.27 $$\begin{aligned} n^{r-1}C\varepsilon (\bar{t}+1)^{r-1}n^{-(r-1)}=C(\bar{t}+1)^{r-1}\varepsilon . \end{aligned}$$

We therefore turn our attention to the quantity

2.28 $$\begin{aligned} \frac{C_1\rho ^{-1}}{C_0^rn^{r-1}}\sum _{{\digamma }\in \Sigma _r}\sum _{\begin{array}{c} {u}\in \\ \mathcal {M}_{n,*}({t},{\digamma },\varvec{s}) \end{array}} \sum _{\begin{array}{c} ({x},{y})\in \\ (\mathbb {Z}^d)^{2r-1} \end{array}} \sum _{\begin{array}{c} T \in \\ \varvec{T}_n({\digamma },{t},{u},{x},{y}) \end{array}} W(T)\prod _{\ell =1}^r \prod _{j_\ell =1}^{m^{\scriptscriptstyle (\ell )}}{\textrm{e}}^{{\textrm{i}}\frac{k_{j_\ell }^{\scriptscriptstyle (\ell )}}{\sqrt{n}}\big ( w_{ns_{j_\ell }^{\scriptscriptstyle (\ell )}}(nt_\ell ,x_\ell )-w_{ns_{j_\ell -1}^{\scriptscriptstyle (\ell )}}(nt_\ell ,x_\ell ) \big )}. \end{aligned}$$

We now define discrete analogues of the sets $\mathcal {I}$ following Definition 2.5. Recall the notation (2.10). Let ${\digamma }\in \Sigma _r$, ${t}\in (0,\infty )^r$, ${u}\in \mathcal {M}_n({t},{\digamma })$, and ${\varvec{s}}=({s}^{\scriptscriptstyle (1)},\dots ,{s}^{\scriptscriptstyle (r)})$, where ${s}^{\scriptscriptstyle (\ell )}=(s_0^{\scriptscriptstyle (\ell )},\dots ,s^{\scriptscriptstyle (\ell )}_{m^{\scriptscriptstyle (\ell )}})$, and $0=s_0^{\scriptscriptstyle (\ell )}<s_1^{\scriptscriptstyle (\ell )}<\dots <s_{m^{\scriptscriptstyle (\ell )}}^{\scriptscriptstyle (\ell )}= t_\ell$ for each $\ell \in [r]$ be given. If $e \notin \mathcal {E}_\ell ({\digamma })$ then set $\mathcal {I}_n(e ,{s}^{\scriptscriptstyle (\ell )})=\varnothing$. If $e\in \mathcal {E}_\ell ({\digamma })$, then let $\mathcal {I}_n(e ,{s}^{\scriptscriptstyle (\ell )})$ denote those elements of $[{s}^{\scriptscriptstyle (\ell )}]_n:=([s^{\scriptscriptstyle (\ell )}_1]_n,\dots , [s^{\scriptscriptstyle (\ell )}_{m^{\scriptscriptstyle (\ell )}}]_n)$ that fall in the interval $(u_-(e),u_+(e)\wedge [t_\ell ]_n)$, where $u_-(e), u_+(e)$ are the elements of ${u}$ corresponding to the endvertices of *e* (and $u_-(e)=0$ if *e* is adjacent to the root). Let $\mathcal {I}_n(e,\varvec{s})=\cup _{\ell =1}^r\mathcal {I}_n(e,{s}^{\scriptscriptstyle (\ell )})$. The $j(e):=|\mathcal {I}_n(e,\varvec{s})|$ elements of $\mathcal {I}_n(e,\varvec{s})$ divide the interval $[u_-(e),u_+(e)\wedge [t_\ell ]_n]$ into $j(e)+1$ subintervals - denote their lengths by $(\check{n}_{e,i}/n)_{i=1,\dots , j(e)+1}$, and set $\check{\varvec{n}}=(\check{n}_{e,i})_{e\in \mathcal {E}({\digamma });i=1,\dots ,j(e)+1}$. If $j(e)=0$ then $\check{n}_{e,1}/n=u_+(e)-u_-(e)$. Note that *j*(*e*) and $\check{\varvec{n}}$ depend on ${\digamma },{u}, \varvec{s}$ (and *n*), and that $\sum _{e\in \mathcal {E}_\ell ({\digamma })}\sum _{j=1}^{j(e)+1}\check{n}_{e,j}=\lfloor nt_\ell \rfloor$.

For $\ell \in [r]$, $e\in \mathcal {E}_{\ell }({\digamma })$ and $a \in \{1,\dots ,j(e)+1\}$ let

$$\begin{aligned} \zeta ^{[e]}_n(a,\ell )=\min \Bigg \{i \le m^{(\ell )}: s^{\scriptscriptstyle (\ell )}_{i}\ge u_-(e)+ \sum _{i_e=1}^a\frac{\check{n}_{e,i_e}}{n}\Bigg \}.\end{aligned}$$

(Note that $s^{\scriptscriptstyle (\ell )}_{i}$ is interchangeable with $[s^{\scriptscriptstyle (\ell )}_{i}]_n$ in the definition of $\zeta ^{[e]}_n$.) Given ${k}^{\scriptscriptstyle (\ell )}=(k_1^{\scriptscriptstyle (\ell )},\dots ,k^{\scriptscriptstyle (\ell )}_{m^{\scriptscriptstyle (\ell )}})\in (\mathbb {R}^d)^{m^{\scriptscriptstyle (\ell )}}$, for each $\ell \in [r]$, and for $e\in \{1,\dots ,2r-1\}$ and $a\le j(e)+1$, let

$$\begin{aligned} \check{k}_{e,a}(n)=\sum _{\ell :e\in \mathcal {E}_\ell ({\digamma })} k^{\scriptscriptstyle (\ell )}_{\zeta ^{[e]}_n(a,\ell )}.\end{aligned}$$

Let $\check{\varvec{k}}(n)=( \check{k}_{e,i}(n))_{e \in [2r-1],i\le j(e)+1}$ which depends on ${\digamma },\varvec{s},{u},n$ and of course $\varvec{k}$.

If $n\in \mathbb {N}$, ${\digamma }\in \Sigma _r$, $\varvec{s}$, and ${u}\in \mathcal {M}_{n,*}({t},{\digamma },\varvec{s})$ are given, this determines $\check{\varvec{n}}=\check{\varvec{n}}({\digamma },\varvec{s},{u})$ as above. If we are given $\varvec{k}$ as well then this also determines $\check{\varvec{k}}(n)$. By expressing locations of paths in terms of their spatial increments $\check{\varvec{y}}=(\check{y}_{e,i})_{i \in [j_e],e\in [2r-1]}$ (and recalling the definition of $\check{\varvec{T}}({\digamma },\check{\varvec{y}},\check{\varvec{n}})$ given prior to (2.22)) we see that (2.28) is equal to

2.29 $$\begin{aligned}&\frac{C_1\rho ^{-1}}{C_0^rn^{r-1}}\cdot \sum _{{\digamma }\in \Sigma _r}\sum _{\begin{array}{c} {u}\in \\ \mathcal {M}_{n,*}({t},{\digamma },\varvec{s}) \end{array}}\sum _{\check{\varvec{y}}}\sum _{\begin{array}{c} T \in \\ \check{\varvec{T}}({\digamma },\check{\varvec{y}},\check{\varvec{n}}({\digamma },\varvec{s},{u})) \end{array}} W(T)\prod _{e=1}^{2r-1}\prod _{i=1}^{j(e)+1}{\textrm{e}}^{{\textrm{i}}\frac{\check{k}_{e,i}(n)}{\sqrt{n}}\cdot \check{y}_{e,i}}\nonumber \\&\quad =\frac{C_1}{C_0^rn^{r-1}}\cdot \sum _{{\digamma }\in \Sigma _r}\sum _{\begin{array}{c} {u}\in \\ \mathcal {M}_{n,*}({t},{\digamma },\varvec{s}) \end{array}}\sum _{\check{\varvec{y}}}\prod _{e=1}^{2r-1}\prod _{i=1}^{j(e)+1}{\textrm{e}}^{{\textrm{i}}\frac{\check{k}_{e,i}(n)}{\sqrt{n}}\cdot \check{y}_{e,i}}\mathbb {P}\Big (\mathcal {T}\in \check{\varvec{T}}\big ({\digamma },\check{\varvec{y}},\check{\varvec{n}}({\digamma },\varvec{s},{u})\big )\Big ), \end{aligned}$$

Recall $\mathcal {M}({t},{\digamma })$ from Definition 2.5. Given $\varepsilon >0$ we define $\mathcal {M}_\varepsilon ({t},{\digamma },\varvec{s})$ to be the set of ${u}\in \mathcal {M}({t},{\digamma })$ for which either:

- there exist a leaf $\ell \in \{1,\dots ,r\}$, a branch point *j* in the path from *o* to $\ell$ in ${\digamma }$, and $i\in \{1,\dots ,m^{\scriptscriptstyle (\ell )}\}$ such that
  $$\begin{aligned}|u_j-s^{(\ell )}_i|\le \varepsilon .\end{aligned}$$
- there exists vertices $i\prec j$ of ${\digamma }$, such that
  $$\begin{aligned}|u_i-u_j|\le \varepsilon .\end{aligned}$$

Let $\mathcal {M}_{*}({t},{\digamma },\varvec{s})=\mathcal {M}({t},{\digamma })\setminus \mathcal {M}_\varepsilon ({t},{\digamma },\varvec{s})$. Then, as for (2.27), we have that

$$\begin{aligned} \int _{{u}\in \mathcal {M}_\varepsilon ({t},{\digamma },\varvec{s})}1 d{u}<C_r\varepsilon \bar{t} \end{aligned}$$

Recall the definition of $\Phi$ (and its arguments) from (2.6). Below we will show that as $n \rightarrow \infty$ (2.29) converges to

2.30 $$\begin{aligned} \sum _{{\digamma }\in \Sigma _r}\int _{{u}\in \mathcal {M}_{*}({t},{\digamma },\varvec{s})}\Phi ({\digamma },{u},{\varvec{s}}, {\varvec{k}})d{u}. \end{aligned}$$

Fix ${\digamma }\in \Sigma _r$ and consider the quantity in (2.29) with fixed ${\digamma }$ which can be written as

2.31 $$\begin{aligned} \frac{C_1}{C_0^rn^{r-1}}\sum _{\begin{array}{c} {u}\in \\ \mathcal {M}_{n,*}({t},{\digamma },\varvec{s}) \end{array}}\sum _{\check{\varvec{y}}}\prod _{e=1}^{2r-1}\prod _{i=1}^{j(e)+1}{\textrm{e}}^{{\textrm{i}}\frac{\check{k}_{e,i}(n)}{\sqrt{n}}\cdot \check{y}_{e,i}}\mathbb {P}\Big (\mathcal {T}\in \check{\varvec{T}}({\digamma },\check{\varvec{y}},\check{\varvec{n}}({\digamma },{u},\varvec{s}))\Big ). \end{aligned}$$

Then (2.31) is equal to

2.32 $$\begin{aligned} \frac{C_1\rho ^{-1}}{C_0^rn^{r-1}}\sum _{\begin{array}{c} {u}\in \\ \mathcal {M}_{n,*}({t},{\digamma },\varvec{s}) \end{array}} \hat{t}^{({\digamma })}_{\check{\varvec{n}}({\digamma },\varvec{s},{u})} \Big (\frac{\check{\varvec{k}}(n)}{\sqrt{n}}\Big ), \end{aligned}$$

where we recall that $\check{\varvec{k}}(n)$ depends on ${\digamma }, \varvec{s},{u},n,\varvec{k}$.

#### Proof of Theorem 2.7

Fix *r*, ${t}$ and the $\phi ^{\scriptscriptstyle (\ell )}$ (hence $\varvec{k}$ and $\varvec{s}$).

Let $\delta (\varvec{s})>0$ denote the minimum difference between distinct values in $\varvec{s}$ (recall that this includes 0 and each $t_\ell$). Let $\varepsilon \in (0,(\delta (\varvec{s})/2)\wedge 1)$. Above (see in particular (2.26), (2.27) and (2.32)), we have shown that the left hand side of (2.7) is equal to

$$\begin{aligned} \frac{C_1\rho ^{-1}}{C_0^rn^{r-1}}\sum _{{\digamma }\in \Sigma _r}\sum _{\begin{array}{c} {u}\in \\ \mathcal {M}_{n,*}({t},{\digamma },\varvec{s}) \end{array}} \hat{t}^{({\digamma })}_{\check{\varvec{n}}({\digamma },\varvec{s},{u})} \Big (\frac{\check{\varvec{k}}(n)}{\sqrt{n}}\Big ) +\mathcal {O}(\varepsilon )+\mathcal {O}(n^{-1}), \end{aligned}$$

where the constants in the $\mathcal {O}$ notation here only depend on $\bar{t}, r, L,d$. By definition of $\check{\varvec{n}}$, each $\check{n}_{e,i}$ is equal to $\lfloor ns \rfloor -\lfloor ns' \rfloor$ for some distinct $s>s'\in \varvec{s}$ (or is equal to $|\lfloor ns^{\scriptscriptstyle (\ell )}_i \rfloor -nu_j|$ for some branch point *j* in the path from *o* to $\ell$ in ${\digamma }$, or $|nu_i-nu_j|$ for some $i\prec j$ in ${\digamma }$). It follows from the definition of $\delta (\varvec{s})$ and the fact that ${u}\in \mathcal {M}_{n,*}({t},{\digamma },\varvec{s})$ that we have that $\check{n}_{e,i}>n\varepsilon /2$ for all *e*, *i* for *n* sufficiently large depending on $\varepsilon$ (which we assume in what follows). By Proposition 2.11 (recalling that $C_0=C_A^2C_V$ and $C_1=C_AC_V$, and $\delta \in (0,1)$ is as in Proposition 2.11) we see that this is equal to

2.33 $$\begin{aligned}&\sum _{{\digamma }\in \Sigma _r}\frac{1}{n^{r-1}} \sum _{\begin{array}{c} {u}\in \\ \mathcal {M}_{n,*}({t},{\digamma },\varvec{s}) \end{array}}\Bigg [\prod _{e=1}^{2r-1} \prod _{i=1}^{j(e)+1}{\textrm{e}}^{-\sigma _0^2\frac{\check{k}_{e,i}^2}{2}\frac{\check{n}_{e,i}}{n}}+ \mathcal {O}\Bigl (\sum _{e,i} \frac{1}{\check{n}_{e,i}^{\frac{d-8}{2}}}\Bigr ) +\mathcal {O}\Bigl (\sum _{e,i} \frac{|\check{\varvec{k}}|^2\check{n}_{e,i}^{1-\delta }}{n}\Bigr )\Bigg ]\nonumber \\&\quad +\mathcal {O}(\varepsilon )+\mathcal {O}(n^{-1}), \end{aligned}$$

where in the above, $\check{\varvec{n}}$ is determined by ${\digamma },{u},\varvec{s}$ (and *n*), and $\check{\varvec{k}}$ is determined by these and $\varvec{k}$. In addition the constants in the error terms in square brackets depends on $\varepsilon$ (among other things, as in Proposition 2.11). Also $\delta ,\varepsilon \in (0,1)$ and $n\varepsilon /2\le \check{n}_{e,i}\le n\bar{t}$ imply the error terms in square brackets are $\mathcal {O}((\varepsilon n)^{-(\delta \wedge (d-8)/2)})$ uniformly in ${u}\in \mathcal {M}_{n,*}({t},{\digamma },\varvec{s})$ (where again the constant in the $\mathcal {O}$ notation here depends on $\varepsilon ,\varvec{k}$). Since the sum over ${u}$ gives at most $(n\bar{t})^{r-1}$ we see that (2.33) is equal to

2.34 $$\begin{aligned} \sum _{{\digamma }\in \Sigma _r}\frac{1}{n^{r-1}} \sum _{\begin{array}{c} {u}\in \\ \mathcal {M}_{n,*}({t},{\digamma },\varvec{s}) \end{array}}\prod _{e=1}^{2r-1} \prod _{i=1}^{j(e)+1}{\textrm{e}}^{-\sigma _0^2\frac{\check{k}_{e,i}^2}{2}\frac{\check{n}_{e,i}}{n}}+\mathcal {O}((\varepsilon n)^{-(\delta \wedge (d-8)/2})+\mathcal {O}(\varepsilon ). \end{aligned}$$

Recall the definition of $\check{\varvec{s}}=\check{\varvec{s}}({\digamma },{u},\varvec{s})$ from below (2.4). Together with the definition of $\check{\varvec{n}}$ we see that $|\check{s}_{e,i}-\check{n}_{e,i}/n|\le 2/n$ for every *e*, *i*. Thus (for *n* large enough depending on $\varepsilon$) (2.34) is equal to

2.35 $$\begin{aligned} \sum _{{\digamma }\in \Sigma _r}\frac{1}{n^{r-1}} \sum _{\begin{array}{c} {u}\in \\ \mathcal {M}_{n,*}({t},{\digamma },\varvec{s}) \end{array}}\prod _{e=1}^{2r-1} \prod _{i=1}^{j(e)+1}{\textrm{e}}^{-\sigma _0^2\frac{\check{k}_{e,i}^2}{2}\check{s}_{e,i}+\mathcal {O}(n^{-1})}+\mathcal {O}(\varepsilon ), \end{aligned}$$

where the error term in the exponent depends on $\varvec{k}$ but is uniform in ${u}$. Recalling (2.6), it follows that (2.35) is equal to

$$\begin{aligned}&\sum _{{\digamma }\in \Sigma _r}\frac{1}{n^{r-1}} \sum _{\begin{array}{c} {u}\in \\ \mathcal {M}_{n,*}({t},{\digamma },\varvec{s}) \end{array}}\prod _{e=1}^{2r-1} \prod _{i=1}^{j(e)+1}{\textrm{e}}^{-\sigma _0^2\frac{\check{k}_{e,i}^2}{2}\check{s}_{e,i}}+\mathcal {O}(\varepsilon )\\&\quad =\sum _{{\digamma }\in \Sigma _r}\frac{1}{n^{r-1}} \sum _{\begin{array}{c} {u}\in \\ \mathcal {M}_{n,*}({t},{\digamma },\varvec{s}) \end{array}}\Phi ({\digamma },{u},\varvec{s},\varvec{k})+\mathcal {O}(\varepsilon ). \end{aligned}$$

As $n\rightarrow \infty$ in the above, the $(r-1)$-fold Riemann sum converges to the $(r-1)$-dimensional integral in (2.30). We have therefore shown that there exists a constant *C* (depending on $\varvec{k},\varvec{s},{t}$) such that for any $\varepsilon >0$, for *n* sufficiently large we have that

$$\begin{aligned}\Bigg |E_{\mu _n^{\scriptscriptstyle {\textrm{LT}}}}\left[ \prod _{\ell =1}^r H^{(n)}_{t_\ell }(\phi ^{\scriptscriptstyle (\ell )})\right] -\sum _{{\digamma }\in \Sigma _r}\int _{{u}\in \mathcal {M}({t},\alpha )} \Phi _{\sigma ^2}({\digamma },{u},{\varvec{s}}, {\varvec{k}}) d{u}\Bigg |\le C\varepsilon ,\end{aligned}$$

which completes the proof. $\square$

## Tightness

In this section we work in an abstract setting for historical processes motivated by the historical paths $\{w(m,x): m\in \mathbb {Z}_+, x\in \mathcal {T}_m\}$ of lattice trees and those for branching Brownian motion, $\{B^\alpha :|\alpha |\in \mathbb {Z}_+,\alpha \in GW\}$ (with $n=1$), both introduced in Sect. 1.

As before, add $\Delta$ to $\mathbb {R}^d$ as a cemetery point. Assume on a probability space $(\Omega ,\mathcal {F},\mathbb {P})$ we have

3.1 $$\begin{aligned}{} & {} \forall k\in \mathbb {Z}_+,\ \mathcal {S}_k \text { is an a.s. finite random subset of a countable set}\, \mathcal {S}. \end{aligned}$$

3.2 $$\begin{aligned}{} & {} \forall k\in \mathbb {Z}_+, \beta \in \mathcal {S},\ (w_j(k,\beta ))_{j\in \mathbb {Z}+} \text { are}\, \mathbb {R}^d\cup \{\Delta \}\text {-valued random variables such that} \nonumber \\{} & {} \quad \text {for }\beta \in \mathcal {S}_k, w_j(k,\beta )\text { are}\, \mathbb {R}^d\text {-valued}, w_0(k,\beta )=0, w_j(k,\beta )=w_k(k,\beta )\ \forall j\ge k,\nonumber \\{} & {} \quad \text {and for }\beta \in \mathcal {S}\setminus \mathcal {S}_k, \, w_j(k,\beta )=\Delta . \end{aligned}$$

So for each $k\in \mathbb {Z}_+$ and $\beta$ in the random finite set $\mathcal {S}_k$ we have a discrete-time $\mathbb {R}^d$-valued stochastic process starting at 0 and freezing at time *k*.

For

3.3 $$\begin{aligned} w\in \mathcal {W}:=\{w(k,\beta ):\beta \in \mathcal {S}_k, k\in \mathbb {Z}_+\}\,\text { (the set of historical paths),} \end{aligned}$$

we define the rescaled paths by

3.4 $$\begin{aligned} w^{\scriptscriptstyle (n)}_s(t,\beta )=\frac{w_{\lfloor ns\rfloor }(\lfloor nt\rfloor ,\beta )}{\sqrt{n}},\quad s,t\ge 0,\end{aligned}$$

so that for $t\ge 0$ and $\beta \in \mathcal {S}_{\lfloor nt\rfloor }$, $w^{\scriptscriptstyle (n)}(t,\beta )\in \mathcal {D}(\mathbb {R}^d)$. Define a càdlàg $\mathcal {M}_F(\mathcal {D}(\mathbb {R}^d))$-valued process by

3.5 $$\begin{aligned} H_t^{\scriptscriptstyle (n)}=\frac{1}{C_gn}\sum _{\beta \in \mathcal {S}_{\lfloor nt\rfloor }}\delta _{w^{\scriptscriptstyle (n)}(t,\beta )}, \end{aligned}$$

where $C_g>0$ is a model-dependent constant. We call this class of measure valued processes, the historical processes associated with $\mathcal {W}$.

### Example 3.1

**(Lattice trees).** Here $\mathcal {S}=\mathbb {Z}^d$, $\mathcal {S}_m=\mathcal {T}_m$ for $m\in \mathbb {Z}_+$ and for $x\in \mathcal {S}_m$, *w*(*m*, *x*) is the tree history from the root to (*m*, *x*) in (1.1). If $C_g=C_0$ then one can easily check that $H^{\scriptscriptstyle (n)}$ as defined in (3.5) agrees with the historical process for lattice trees in (1.4). Note here that the index set for $w^{\scriptscriptstyle (n)}$ has been changed from that in (1.2) (and so we have abused the notation) but the actual empirical measures are unchanged. Properties (3.1) and (3.2) are clear if we extend the definition of *w*(*m*, *x*) to $\Delta$ for $x\notin \mathcal {S}_m$.

### Example 3.2

**(Branching random walk).** We discretize (in time) the branching Brownian motions introduced in Sect. 1 and use the notation from that construction. We denote dependence on $n\in \mathbb {N}$ now in our notation for $\hat{B}^{\beta ,\scriptscriptstyle (n)}$ for $\beta \in I$. Let $\mathcal {S}=I$, $\mathcal {S}_m=\{\beta \in I:\beta \in GW, |\beta |=m\}$, and for $\beta \in \mathcal {S}_m$ set

$$\begin{aligned} w_j(m,\beta )=\hat{B}^{\beta ,\scriptscriptstyle (1)}_{j\wedge m}.\end{aligned}$$

Then one can check that for $\alpha \in \mathcal {S}_{\lfloor nt\rfloor }$,

$$\begin{aligned} w^{\scriptscriptstyle (n)}_s(t,\beta )=\frac{\hat{B}^{\beta ,\scriptscriptstyle (1)}_{\lfloor n(t\wedge s)\rfloor }}{n^{1/2}}.\end{aligned}$$

Set $C_g=1$ in (3.5), and for $|\beta |=\lfloor nt\rfloor$, let $Z^{\beta ,\scriptscriptstyle (n)}_s=\hat{B}^{\beta ,\scriptscriptstyle (n)}_{\lfloor n(s\wedge t)\rfloor /n}$ be a time discretization of the stopped Brownian paths $\hat{B}^{\beta ,\scriptscriptstyle (n)}$. Brownian scaling shows that

3.6 $$\begin{aligned}&\text { if }\tilde{H}^{\scriptscriptstyle (n)}_t=\frac{1}{n}\sum _{\begin{array}{c} \beta \in GW:\\ |\beta |=\lfloor nt\rfloor \end{array}}\delta _{Z^{\beta ,\scriptscriptstyle (n)}}, \text { then}\, \tilde{H}^{\scriptscriptstyle (n)}\,\text { is equal in law to}\nonumber \\&\quad \text {the}\,n\text {th historical process given by (3.5) for each}\, n\in \mathbb {N}. \end{aligned}$$

Clearly $\tilde{H}^{\scriptscriptstyle (n)}$ is a rescaled branching random walk with Gaussian mean 0, variance $\sigma ^2$ increments. Properties (3.1) and (3.2) are again clear if we extend the definition of *w*(*m*, *x*) to $\Delta$ for $x\notin \mathcal {S}_m$.

In order to prove historical tightness, we will assume that the collection $\mathcal {W}$ (as in (3.3)) of historical paths satisfies the following condition. Recall that $w^{\scriptscriptstyle (n)}$ is the scaled version of *w*, as in (3.4).

### Condition 3.3

(*Modulus of continuity*). For some $q\in (0,1/2),\theta \in (0,1]$, and constant $C_2>0$, there exist random variables $(\delta _n)_{n \in \mathbb {N}}$ so that for all historical paths $w\in {\mathcal {W}}$ and $n\in \mathbb {N}$,

3.7 $$\begin{aligned}&\forall s_i\in \mathbb {Z}_{+}/n, \quad |s_2-s_1|\le \delta _n\Rightarrow |w^{\scriptscriptstyle (n)}_{s_2}-w^{\scriptscriptstyle (n)}_{s_1}|\le |s_2-s_1|^q,\nonumber \\&\quad \text {where }n\mathbb {P}(\delta _n\le \rho )\le C_2\rho ^\theta \quad \forall \rho \in [0,1). \end{aligned}$$

This condition is verified for any $q\in (0,1/2)$ and $\theta =1$ in [20, Theorem 6] for sufficiently spread-out lattice trees in more than 8 dimensions in Example 3.1 above (as well as a number of other models)—see Lemma 3.12 below. For the Branching Random Walks with Gaussian increments in Example 3.2 it is easy to derive it from [6, Theorem 8.1] for the same parameter values (in fact $\theta$ can be taken to be any value in $(0,\infty )$) . Here one takes the underlying diffusion to be Brownian motion, restricts the time steps to be in $\mathbb {Z}_+/n$, and then uses (3.6).

In our abstract setting, the extinction times become

$$\begin{aligned} S^{\scriptscriptstyle (1)}=\min \{k\in \mathbb {Z}_+: \mathcal {S}_k=\emptyset \}\in \mathbb {Z}_+\cup {\infty }, \end{aligned}$$

so that

$$\begin{aligned} S^{\scriptscriptstyle (n)}:=S^{\scriptscriptstyle (1)}/n=\inf \{t\ge 0: H^{\scriptscriptstyle (n)}_t(1)=0\}, \end{aligned}$$

agreeing with our earlier definition for lattice trees. We assume $S^{\scriptscriptstyle (1)}$ satisfies the following:

### Condition 3.4

(*Survival bounds*). There exist $\underline{c},\overline{c}>0$ such that

3.8 $$\begin{aligned} \underline{c}\le \inf _{t\ge 0}\mathbb {P}(S^{\scriptscriptstyle (1)}>t)(t\vee 1)\le \sup _{t\ge 0}\mathbb {P}(S^{\scriptscriptstyle (1)}>t)(t\vee 1)\le \overline{c}. \end{aligned}$$

This condition holds for the branching random walks in Example 3.2 by Kolmogorov’s classical result for survival of critical branching processes (e.g. see [24, Theorem II.1.1(a)]) and for the lattice tree historical paths in Example 3.1 by (1.6) (or see [20, (1.22) and (1.27)]).

### Definition 3.5

For a metric space, *E*, a collection $\{Q_n:n\in \mathbb {N}\}$ of probabilities on $\mathcal {D}(\mathbb {R}_+,E)=\mathcal {D}(E)$, is $\mathcal {C}$*-relatively compact* iff every sequence $n_k\rightarrow \infty$ has a subsequence $\{n'_k\}$ s.t. $Q_{n'_k}$ converges weakly in $\mathcal {D}(E)$ to a law, *Q*, supported on $\mathcal {C}(E)$, the set of continuous *E*-valued paths. If $\{X_n\}$ is a sequence of càdlàg *E*-valued processes on our underlying probability space, we say $\{X_n:n\in \mathbb {N}\}$ is $\mathcal {C}^{\text {cond}}$*-relatively compact* iff for every $s_0>0$, the set of conditional laws $\{\mathbb {P}(X_n\in \cdot |S^{\scriptscriptstyle (n)}>s_0):n\in \mathbb {N}\}$ is $\mathcal {C}$-relatively compact in $\mathcal {D}(E)$. $\blacktriangleleft$

We start with a general tightness result for historical processes in this abstract setting:

### Theorem 3.6

Assume $H^{\scriptscriptstyle (n)}$ is given by (3.5), where $\mathcal {W}$ satisfies Condition 3.3. Suppose also that Condition 3.4 holds and $\{H^{\scriptscriptstyle (n)}_\cdot (\phi ):n\in \mathbb {N}\}$ is $\mathcal {C}^{\text {cond}}$-relatively compact in $\mathcal {D}(\mathbb {C})$ for each $\phi$ in a determining class $D_0$ (for $\mathcal {M}_F(\mathcal {D}(\mathbb {R}^d))$) containing 1. Then $\{H^{\scriptscriptstyle (n)}_\cdot :n\in \mathbb {N}\}$ is $\mathcal {C}^{\text {cond}}$-relatively compact, and for every $s_0>0$, every limit point, *H*, of $\{\mathbb {P}(H^{\scriptscriptstyle (n)}\in \cdot |S^{(n)}>s_0):n\in \mathbb {N}\}$ satisfies $H_t(\mathcal {C}(\mathbb {R}^d)^c)=0$ for all $t\ge 0$ a.s.

In practice it is the relative compactness of $\{H^{\scriptscriptstyle (n)}_\cdot (\phi ):n\in \mathbb {N}\}$ for a rich class of test functions $\phi$ that will require most of the effort. For LT’s this is done in Proposition 3.11, which is in turn proved in Sect. 3.2 below. Applying Theorem 3.6 to the case of lattice trees (conditional on survival), we will then deduce the following below:

### Theorem 3.7

Let $H^{\scriptscriptstyle (n)}$ be the sequence of rescaled historical processes associated with sufficiently spread-out lattice trees in $d>8$ dimensions, defined in (1.4). Then $\{H^{\scriptscriptstyle (n)}_\cdot :\, n\in \mathbb {N}\}$ is $\mathcal {C}^{\text {cond}}$-relatively compact.

### Proofs of Theorems 3.6 and 3.7

Our starting point for proving Theorem 3.6 is a version of the Jakubowski-Kurtz Theorem for $\mathcal {M}_F(\mathcal {D}(\mathbb {R}^d))$-valued processes. It is a simple extension of that for $\mathcal {M}_F(\mathbb {R}^d)$-valued processes in [11, Theorem 5.2].

#### Theorem 3.8

Let $D_0\subset \mathcal {C}(\mathcal {D}(\mathbb {R}^d),\mathbb {C})$ be a determining class for $\mathcal {M}_F(\mathcal {D}(\mathbb {R}^d))$ containing 1.

A sequence of probabilities $\{P_k,\,k\in \mathbb {N}\}$ on $\mathcal {D}(\mathcal {M}_F(\mathcal {D}(\mathbb {R}^d)))$ is $\mathcal {C}$-relatively compact iff

3.9 $$\begin{aligned}&\forall \,\eta>0,\ \forall T\in \mathbb {N},\text { there is a compact set}\, K_{\eta ,T}\subset \mathcal {D}(\mathbb {R}^d)\,\text { such that} \nonumber \\&\quad \sup _kP_k\Big (\sup _{t\le T}H_t(K_{\eta ,T}^c)>\eta \Big )<\eta , \end{aligned}$$

and

3.10 $$\begin{aligned}&\text { for all }\phi \in D_0\text { the sequence of probabilities, }\{P_k(H_\cdot (\phi )\in \cdot )\},\nonumber \\&\quad \text { is}\,\mathcal {C}\text {-relatively compact in}\, \mathcal {D}(\mathbb {C}). \end{aligned}$$

For $\delta ,T>0$ and $w\in \mathcal {D}(\mathbb {R}^d)$, we define

$$\begin{aligned} W'(w,\delta ,T)=\inf _{\{t_i\}}\max _i\sup _{s,t\in [t_{i-1},t_i)}|w_s-w_t|,\end{aligned}$$

where the infimum is over all partitions $\{t_i\}$ such that $0=t_0<t_1<\dots t_{N-1}<T\le t_N$ such that $t_i-t_{i-1}>\delta$ for all *i*. Note that $W'$ is decreasing in $\delta$ and increasing in *T*. We restate [8, Ch. 3, Theorem 6.3 and Remark 6.4] with their general metric space *E* replaced by $\mathbb {R}^d$ and use the above monotonicity to take sequential limits and restrict $T\in \mathbb {N}$.

#### Proposition 3.9

Let $\delta '_m\downarrow 0$. The closure of a set $A\subset \mathcal {D}(\mathbb {R}^d)$ is compact iff

$$\begin{aligned} \sup _{w\in A, t\le T}|w_t|<\infty \quad \text { and }\quad \lim _{m\rightarrow \infty }\sup _{w\in A}W'(w,\delta '_m,T)=0, \quad \forall \, T\in \mathbb {N}. \end{aligned}$$

For $0{<}q{<}1/2$ let $B_m{=}B_m(q){=}\{w\in \mathcal {D}(\mathbb {R}^d):W'(w,2^{-m},T){\le } 2^{-(m-2)q}\forall \, T{\in } \mathbb {N}\}$, and for $M\in \mathbb {N}$ define

$$\begin{aligned} A_M=A_M(q)=\{w\in \mathcal {D}(\mathbb {R}^d):|w_t|\le (t+1)2^{M+1}\ \forall t\ge 0\}\cap \Bigl (\cap _{m=M}^\infty B_m\Bigr ).\end{aligned}$$

An easy application of Proposition 3.9 shows that $A_M$ has compact closure in $\mathcal {D}(\mathbb {R}^d)$.

#### Lemma 3.10

Assume Condition 3.3, and let $q, \delta _n$ be as in (3.7). For any $n,M\in \mathbb {N}$, if $\delta _n>\max (2^{2-M},n^{-1})$, then $H^{\scriptscriptstyle (n)}_t(A_M^c)=0$ for all $t\ge 0$.

#### Proof

Assume $\delta _n\ge \max (2^{2-M},n^{-1})$, and let $m\in \mathbb {N}^{\ge M}$, $T\in \mathbb {N}$ and $w\in {\mathcal W}$. If we divide $[0,\lceil t\rceil ]$ into $\lceil t\rceil 2^M$ intervals of length $2^{-M}<\delta _n$, then the triangle inequality, (3.7) and $\delta _n\ge n^{-1}$ imply

3.11 $$\begin{aligned} |w^{\scriptscriptstyle (n)}_t|=|w^{\scriptscriptstyle (n)}_{[t]_n}-w^{\scriptscriptstyle (n)}_0|\le \lceil t\rceil 2^M[2^{-Mq}+n^{-q}]\le (t+1)2^{M+1}, \end{aligned}$$

where in the first inequality we have moved an interval endpoint to an appropriate neighbouring point in $\mathbb {Z}_{+}/n$ resulting in an error of at most $n^{-q}$. Consider next $W'(w^{\scriptscriptstyle (n)},2^{-m},T)$ for $w\in \mathcal {W}$. If $2^{-m}<\frac{1}{n}$, then $W'(w^{\scriptscriptstyle (n)},2^{-m},T)=0$, as one can see by taking $t_i=\frac{i}{n}$, $i\in \mathbb {Z}_+$ in the definition of $W'$, and using the fact that $w^{\scriptscriptstyle (n)}$ is constant on $[i/n,(i+1)/n)$ for $i\in \mathbb {Z}_+$. Assume therefore that $2^{-m}\ge \frac{1}{n}$. Now set $t_i=i 2^{-m+1}$, for $i\in \mathbb {Z}_+$, which gives $t_i-t_{i-1}>2^{-m}$ for all *i*. We also have

3.12 $$\begin{aligned} {[}t_{i}]_n-[t_{i-1}]_n\le 2^{1-m}+\frac{1}{n}< 2^{2-m}\le 2^{2-M}\le \delta _n. \end{aligned}$$

By (3.7) this implies that for $s,t\in [t_{i-1},t_i)$

$$\begin{aligned} |w^{\scriptscriptstyle (n)}_t-w^{\scriptscriptstyle (n)}_s|&=|w^{\scriptscriptstyle (n)}_{[t]_n}-w^{\scriptscriptstyle (n)}_{[s]_n}|\le |[t]_n-[s]_n|^q\le 2^{-(m-2)q}, \end{aligned}$$

where in the last line we have used the middle expression in (3.12). This proves that $W'(w^{\scriptscriptstyle (n)},2^{-m},T)\le 2^{-(m-2)q}$, which together with (3.11), shows that $w^{\scriptscriptstyle (n)}\in A_M$, and so completes the proof. $\square$

#### Proof of Theorem 3.6

Let $n_k\rightarrow \infty$, fix $s_0>0$, and define probabilities on $\mathcal {D}(\mathcal {M}_F(\mathcal {D}(\mathbb {R}^d)))$ by

$$\begin{aligned} P_{n_k}(\cdot )=\mathbb {P}(H^{\scriptscriptstyle (n_k)}\in \cdot |S^{\scriptscriptstyle (n_k)}>s_0).\end{aligned}$$

For the first assertion we need to show this sequence of probability laws are $\mathcal {C}$-relatively compact on $\mathcal {D}(\mathcal {M}_F(\mathcal {D}(\mathbb {R}^d)))$. For this we will use Theorem 3.8, and so need to verify the hypotheses of that result. For (3.9), for all $T\in \mathbb {N}$ we set $K_{\eta ,T}=\overline{A_M}$, where *M* is chosen below. The compactness of this set follows from Proposition 3.9, as has already been noted above. By Lemma 3.10,

$$\begin{aligned} P_{n_k}\big (\sup _t H_t(K^c_{\eta ,T})>0\big )&\le \mathbb {P}\big (H^{\scriptscriptstyle (n_k)}_t(A_M^c)>0\ \ \text { for some } t\ge 0\big |\, S^{\scriptscriptstyle (n_k)}>s_0\big )\\&\le \mathbb {P}\big (\delta _{n_k}\le \max (2^{2-M},n_k^{-1})\big )/\mathbb {P}(S^{\scriptscriptstyle (n_k)}>s_0)\\&\le \underline{c}^{-1}(n_ks_0+1)n_k^{-1}C_2(2^{(2-M)\theta }+n_k^{-\theta }), \end{aligned}$$

where in the last inequality we have used (3.8) and (3.7). The above bound is at most $\underline{c}^{-1}(s_0+1)C_2(2^{(2-M)\theta }+n_k^{-\theta })$ which will be smaller than $\eta$ if we set $M=M(\eta )$ large enough and assume $n_k>N(\eta )$. This proves (3.9) for large enough *k*. It is easy to enlarge $K_{\eta ,T}$ to obtain a compact set which satisfies (3.9) for all *k*. For example, for fixed $n=n_k\le N(\eta )$ and all $t\ge 0$, $H^{\scriptscriptstyle (n)}_t$ is supported on the space of càdlàg paths which are constant on $[i/n,(i+1)/n)$ and on $[S^{\scriptscriptstyle (n)},\infty )$, and whose jumps are uniformly bounded in absolute value by

3.13 $$\begin{aligned} \max _{m/n\le S^{\scriptscriptstyle (n)}, \beta \in \mathcal {S}_m,1\le j\le m}\frac{|w_j(m,\beta )-w_{j-1}(m,\beta )|}{n^{1/2}}<\infty \ \ P_n-a.s.\end{aligned}$$

Now use (3.8) to bound $S^{\scriptscriptstyle (n)}$ and bound the upper bound on the jumps in (3.13), with high $P_n$-probability, and so obtain a compact set of paths which supports $H^{\scriptscriptstyle (n)}_t$ for all $t\ge 0$ with $P_n$ probability at least $1-\eta$, for the finite many values of $n=n_k\le N(\eta )$.

The other condition (3.10) of Theorem 3.8 holds by assumption and so the $\mathcal {C}$-relative compactness is established.

For the last statement we note first that if $\Delta w_t=w_t-w_{t-}$ for $w\in \mathcal {D}(\mathbb {R}^d)$ and $t>0$, then a simple Skorokhod topology exercise (e.g. use [8, Chapter 3, Proposition 5.3]) shows that for any $\delta >0$,

$$\begin{aligned} \Big \{w\in \mathcal {D}(\mathbb {R}^d):\, \sup _{s>0}\, |\Delta w_s|\le \delta \Big \}\text { is a closed set in }\mathcal {D}(\mathbb {R}^d). \end{aligned}$$

Consider a weak limit *H* of $\{P_{n_k}\}$. By Skorokhod’s representation theorem and the continuity of the limit point, $H_\cdot$, we may realize all our processes on a space with underlying law $\mathbb {P}'$ and assume $H^{\scriptscriptstyle (n_k)}_t\rightarrow H_t$ in $\mathcal {M}_F(\mathcal {D}(\mathbb {R}^d))$ for all $t\ge 0$, $\mathbb {P}'$-a.s. So the Portmanteau Theorem for the weak topology gives for all $t\ge 0$ and $M\in \mathbb {N}$,

$$\begin{aligned}&H_t\Big (\big \{\sup _{s>0}|\Delta w_s|\le 1/M\big \}^c\Big )\le \liminf _{k\rightarrow \infty }H_t^{\scriptscriptstyle (n_k)}\Big (\big \{\sup _{s>0}|\Delta w_s|\le 1/M\big \}^c\Big ). \end{aligned}$$

Now fix $t>0$ and use Fatou’s Lemma to see that for $\delta >0$,

$$\begin{aligned} \mathbb {P}'\Big (H_t\big (\big \{\sup _{s>0}|\Delta w_s|>1/M\big \}\big )>\delta \Big )&\le \mathbb {P}'\Big ( \liminf _{k\rightarrow \infty }H_t^{\scriptscriptstyle (n_k)}(\{\sup _{s>0}|\Delta w_s|> 1/M\})>\delta \Big )\\&\le \liminf _{k\rightarrow \infty }\mathbb {P}'\Big (H_t^{\scriptscriptstyle (n_k)}(\{\sup _{s>0}|\Delta w_s|> 1/M\})>\delta \Big )\\&\le \liminf _{k\rightarrow \infty }P_{n_k}(\delta _{n_k}<1/n_k)=0. \end{aligned}$$

In the last inequality we use the fact that for *k* large enough $\delta _{n_k}\ge 1/n_k$ implies that for all ancestral paths, and all $s>0$, $|\Delta w^{\scriptscriptstyle (n_k)}_s|\le (1/n_k)^q <1/M$, and in the final equality we use Conditions 3.3 and 3.4. Now let $M\uparrow \infty$ to see that $H_t$ is supported by $\mathcal {C}=\mathcal {C}(\mathbb {R}^d)$ a.s. for each $t>0$. Therefore $H_t(\mathcal {C}^c)=0$ $\forall t\in \mathbb {Q}^{>0}$. So using the openness of $\mathcal {C}^c$ and the Portmanteau theorem again, we get from the continuity of $t\rightarrow H_t$ that $H_t(\mathcal {C}^c)=0$ for all $t\ge 0$ a.s. $\square$

Let Lip$_K$ denote the set of functions $\phi :\mathcal {D}(\mathbb {R}^d)\rightarrow \mathbb {R}$ such that for each $w,w'\in \mathcal {D}(\mathbb {R}^d)$, $|\phi (w)|\le K$ and $|\phi (w)-\phi (w')|\le K\Vert w-w'\Vert$, where $\Vert w\Vert =\sup _{t\in \mathbb {R}_+}|w_t|$.

#### Proposition 3.11

For critical sufficiently spread-out lattice trees in dimensions $d>8$: For each $\phi \in \textrm{Lip}_1$, $\{H^{\scriptscriptstyle (n)}_\cdot (\phi ):n\in \mathbb {N}\}$ is $\mathcal {C}^{\text {cond}}$-relatively compact in $\mathcal {D}(\mathbb {R})$.

The proof of this key result is more complicated and so is deferred until Sect. 3.2. Assuming this, Theorem 3.7 now follows:

#### Proof of Theorem 3.7

We have already noted that the historical process for lattice trees is a special case of the general framework in this Section, that Condition 3.3 was verified in [20] with $q=1/4$ and $\theta =1$ (see Lemma 3.12 below), and Condition 3.4 holds by (1.6). Proposition 3.11 shows the last hypothesis of Theorem 3.6 holds with $D_0=\textrm{Lip}_1$. $D_0$ is a determining class because it includes appropriate multiples of all finite-dimensional Lipschitz continuous functions. The result now follows from Theorem 3.6.

$$\square $$

One can also prove the analogue of Theorem 3.7 for the branching random walks in Example 3.2, where the analogue of Proposition 3.11 yields easily to martingale methods, but the convergence results here can be readily proved as in [24, Chapter II].

### Tightness for lattice trees

The goal of this section is to prove Proposition 3.11. For lattice trees, we will use the modulus of continuity in the following form:

#### Lemma 3.12

For each $n\in \mathbb {N}$ there exists a random $\delta _n\ge \frac{1}{n}$ and a constant $c>0$ satisfying $n\mathbb {P}(\delta _n\le \rho )\le c\rho$ for every $\rho \in [0,1)$ and every $w\in \mathcal {W}$ (the system of ancestral paths to points in the tree)

$$\begin{aligned} |s_2-s_1|\le \delta _n \Rightarrow |w^{\scriptscriptstyle (n)}_{s_2}-w^{\scriptscriptstyle (n)}_{s_1}|\le c(|s_2-s_1|^{1/4}+n^{-1/4}).\end{aligned}$$

#### Proof

Apply [20, Theorem 6] with $\alpha =1/4$. The fact that we can take $\delta _n\ge \frac{1}{n}$ follows from the finite-range assumption on the lattice trees, which gives $|w^{\scriptscriptstyle (n)}_{i/n}-w^{\scriptscriptstyle (n)}_{(i-1)/n}|\le Ln^{-1/2}\le Ln^{-1/4}$, and so allows us to replace $\delta _n$ with $\delta _n\vee (1/n)$. $\square$

The other main ingredient we use is a bound on the fourth moments of the increments of the total mass:

#### Proposition 3.13

There is a $\gamma >1$ and for any $T>0$, there is a $c_T$ such that for all $n\in \mathbb {N}$ and all $s_1,s_2\in (\mathbb {Z}_{+}/n)\cap [0,T]$,

$$\begin{aligned} n\mathbb {E}\big [(H^{\scriptscriptstyle (n)}_{s_2}(1)-H^{\scriptscriptstyle (n)}_{s_1}(1))^4\big ]\le c_T|s_2-s_1|^\gamma .\end{aligned}$$

The above is condition (ii) of [11, Theorem 2.2] with $k=0$ and is verified in that reference (see [11, Theorem 3.3, Lemma 3.5, and Section 7]).

For $w \in \mathcal {D}(\mathbb {R}^d)$ and $t\ge 0$ let $w^t\in \mathcal {D}(\mathbb {R}^d)$ be defined by $w^t_s=w_{s \wedge t}$ and for $\phi \in$ Lip$_1$ let $\phi ^t\in \textrm{Lip}_1$ be defined by $\phi ^t(w)=\phi (w^t)$. Define $\mathcal {T}^{\scriptscriptstyle (n)}_t=n^{-1/2}\mathcal {T}_{nt}$. We will use $\mathcal {T}^{\scriptscriptstyle (n)}_t$ as our index set for $w^{\scriptscriptstyle (n)}$, as in (1.2), and so depart from the notation in (3.4).

#### Lemma 3.14

Let $\delta _n$ be as in Lemma 3.12, and assume that $0\le v\le t_1<t_2$ satisfy

$$\begin{aligned} t_2-v\le \delta _n(\omega ). \end{aligned}$$

Then for $\phi \in \textrm{Lip}_1$ and $i=1,2$,

$$\begin{aligned} |H^{\scriptscriptstyle (n)}_{t_i}(\phi )-H^{\scriptscriptstyle (n)}_{t_i}(\phi ^v)|\le c((t_2-v)^{1/4}+n^{-1/4})(X^{\scriptscriptstyle (n)}_{t_1}(1)\vee X^{\scriptscriptstyle (n)}_{t_2}(1)).\end{aligned}$$

#### Proof

Note that $H^{\scriptscriptstyle (n)}_{t}(\phi ^t)=H^{\scriptscriptstyle (n)}_{t}(\phi )$ (recall (1.1), (1.2), and (1.4)), and therefore for $t_i$ and *v* as above,

$$\begin{aligned} |H^{\scriptscriptstyle (n)}_{t_i}(\phi )-H^{\scriptscriptstyle (n)}_{t_i}(\phi ^{v})|&\le \frac{c}{n}\sum _{x \in \mathcal {T}^{\scriptscriptstyle (n)}_{t_i}}\big |\phi \big (w^{\scriptscriptstyle (n)}(t_i,x)\big )-\phi \big ((w^{\scriptscriptstyle (n)}(t_i,x))^{v}\big )\big |\\&\le \frac{c}{n}\sum _{x \in \mathcal {T}^{\scriptscriptstyle (n)}_{t_i}}\Vert w^{\scriptscriptstyle (n)}(t_i,x) - (w^{\scriptscriptstyle (n)}(t_i,x))^{v}\Vert \\&= \frac{c}{n}\sum _{x \in \mathcal {T}^{\scriptscriptstyle (n)}_{t_i} }\sup _{s \in [v,t_i]}|w^{\scriptscriptstyle (n)}_s(t_i,x)-w^{\scriptscriptstyle (n)}_{v}(t_i,x)|\\&\le c(|t_i-v|^{1/4}+n^{-1/4})X^{\scriptscriptstyle (n)}_{t_i}(1), \end{aligned}$$

where we have used $t_2-v\le \delta _n$ and Lemma 3.12 in the last line. The result follows. $\square$

For a lattice tree *T* containing *x* (and *o*), let $T_{\ngtr x}$ denote the tree consisting of all vertices that are not descendants of *x*. If $x \notin T$ then let $T_{\ngtr x}=\varnothing$. Let $\mathcal {F}_{\ngtr x}=\sigma (\mathcal {T}_{\ngtr x})$. Let $\mathbb {T}_x$ denote the set of lattice trees containing the vertex *x*. If $x \in T$, let $R_x(T)\in \mathbb {T}_x$ denote the descendants of *x* in *T* together with *x* and all the edges joining them (if $x \notin T$, let $R_x(T)=\varnothing$), and let $R_x(T)-x\in \mathbb {T}_o$ denote the translation of $R_x(T)$ by $-x$.

#### Lemma 3.15

For $x\in \mathbb {Z}^d$, for every Borel measurable $\varphi ^*:\mathbb {T}_o \rightarrow \mathbb {R}_+$, and $\varphi :\{(x,R):x\in \mathbb {Z}^d, R\in \mathbb {T}_x\}\rightarrow \mathbb {R}_+$ defined by $\varphi (x,R)=\varphi ^*(R-x)$ a.s.

$$\begin{aligned} \mathbb {E}\left[ \varphi (x,R_x(\mathcal {T}))\big |\mathcal {F}_{\ngtr x}\right] \mathbb {1}_{\{x \in \mathcal {T}\}}\le \rho \mathbb {E}[\varphi (o,\mathcal {T})]\mathbb {1}_{\{x \in \mathcal {T}\}}.\end{aligned}$$

#### Proof

Let $\varphi ^*(R):=\mathbb {1}_{\{R \in F\}}$, where $F\subset \mathbb {T}_o$. For *S* such that $\mathbb {P}(x \in \mathcal {T}, \mathcal {T}_{\ngtr x}=S)>0$,

$$\begin{aligned}&\mathbb {E}\left[ \varphi (x,R_x(\mathcal {T}))\big |\mathcal {T}_{\ngtr x}=S, \,x \in \mathcal {T}\right] \mathbb {1}_{\{\mathcal {T}_{\ngtr x}=S\}}\mathbb {1}_{\{x \in \mathcal {T}\}}\\&\quad =\sum _{{n\in \mathbb {Z}_+}}\dfrac{\mathbb {E}[\mathbb {1}_{\{R_x(\mathcal {T})-x\in F\}}\mathbb {1}_{\{\mathcal {T}_{\ngtr x}= S\}}\mathbb {1}_{\{x\in \mathcal {T}_n\}}]}{\mathbb {P}(\mathcal {T}_{\ngtr x}= S,x \in \mathcal {T})}\mathbb {1}_{\{\mathcal {T}_{\ngtr x}=S\}}\mathbb {1}_{\{x \in \mathcal {T}\}} \end{aligned}$$

By [20, Lemma 9.4] this is at most

$$\begin{aligned}&\rho \mathbb {P}(\mathcal {T}\in F)\sum _n\dfrac{\mathbb {E}[\mathbb {1}_{\{\mathcal {T}_{\ngtr x}= S\}}\mathbb {1}_{\{x\in \mathcal {T}_n\}}]}{\mathbb {P}(\mathcal {T}_{\ngtr x}= S,x \in \mathcal {T})}\mathbb {1}_{\{\mathcal {T}_{\ngtr x}=S\}}\mathbb {1}_{\{x \in \mathcal {T}\}}=\rho \mathbb {P}(\mathcal {T}\in F)\mathbb {1}_{\{\mathcal {T}_{\ngtr x}=S\}}\mathbb {1}_{\{x \in \mathcal {T}\}}. \end{aligned}$$

Summing over *S* gives

$$\begin{aligned} \mathbb {E}\left[ \varphi (x,R_x(\mathcal {T}))\big |\mathcal {F}_{\ngtr x}\right] \mathbb {1}_{\{x \in \mathcal {T}\}}\le \rho \mathbb {P}(\mathcal {T}\in F)\mathbb {1}_{\{x \in \mathcal {T}\}}.\end{aligned}$$

The right-hand side is equal to $\rho \mathbb {E}[\varphi (o,\mathcal {T})]\mathbb {1}_{\{x \in \mathcal {T}\}}$ as claimed. Use linearity to get the result for simple non-negative functions, and monotone convergence to complete the proof. $\square$

Assume $0\le v\le t_1<t_2$ and $\phi \in \textrm{Lip}_1$. We want to bound

3.14 $$\begin{aligned} |H^{\scriptscriptstyle (n)}_{t_2}(\phi )-H^{\scriptscriptstyle (n)}_{t_1}(\phi )|&\le |H^{\scriptscriptstyle (n)}_{t_2}(\phi )-H^{\scriptscriptstyle (n)}_{t_2}(\phi ^{v})|+|H^{\scriptscriptstyle (n)}_{t_2}(\phi ^{v})-H^{\scriptscriptstyle (n)}_{t_1}(\phi ^{v})|\nonumber \\&\quad +|H^{\scriptscriptstyle (n)}_{t_1}(\phi )-H^{\scriptscriptstyle (n)}_{t_1}(\phi ^{v})|. \end{aligned}$$

Lemma 3.14 will allow us to handle the first and last terms; the majority of the work will be in bounding the expected 4th power of the middle term. For fixed *n*, $T\in \mathbb {T}_o$ and $x\in n^{-1/2}\mathbb {Z}^d$, let $R^{\scriptscriptstyle (n)}_x(T^{\scriptscriptstyle (n)})=n^{-1/2}R_{\sqrt{n}x}(T)\subset T^{\scriptscriptstyle (n)}$ denote the subtree consisting of *x* and its descendants. Write $\mathcal {R}^{\scriptscriptstyle (n)}_x=R^{\scriptscriptstyle (n)}_x(\mathcal {T}^{\scriptscriptstyle (n)})$.

$$\begin{aligned}&H^{\scriptscriptstyle (n)}_{t_2}(\phi ^{v})-H^{\scriptscriptstyle (n)}_{t_1}(\phi ^{v})=\frac{1}{C_0n} \left[ \sum _{z_2\in \mathcal {T}^{\scriptscriptstyle (n)}_{t_2}}\phi ((w^{\scriptscriptstyle (n)}(t_2,z_2))^{v}) -\sum _{z_1\in \mathcal {T}^{\scriptscriptstyle (n)}_{t_1}}\phi ((w^{\scriptscriptstyle (n)}(t_1,z_1))^{v})\right] . \end{aligned}$$

Using the tree structure and $v\le t_1<t_2$, this is equal to

$$\begin{aligned}&\frac{1}{C_0n}\left[ \sum _{x\in \mathcal {T}^{\scriptscriptstyle (n)}_{v}}\sum _{z_2 \in \mathcal {T}^{\scriptscriptstyle (n)}_{t_2}\cap \mathcal {R}^{\scriptscriptstyle (n)}_x}\phi ((w^{\scriptscriptstyle (n)}(t_2,z_2))^{v})-\sum _{x\in \mathcal {T}^{\scriptscriptstyle (n)}_{v}}\sum _{z_1 \in \mathcal {T}^{\scriptscriptstyle (n)}_{t_1}\cap \mathcal {R}^{\scriptscriptstyle (n)}_x}\phi ((w^{\scriptscriptstyle (n)}(t_1,z_1))^{v})\right] \\&\quad =\frac{1}{C_0n}\left[ \sum _{x\in \mathcal {T}^{\scriptscriptstyle (n)}_{v}}\phi (w^{\scriptscriptstyle (n)}(v,x))\Big (\sum _{z_2 \in \mathcal {T}^{\scriptscriptstyle (n)}_{t_2}\cap \mathcal {R}^{\scriptscriptstyle (n)}_x}1-\sum _{z_1 \in \mathcal {T}^{\scriptscriptstyle (n)}_{t_1}\cap R_x(\mathcal {T})}1\Big )\right] \\&\quad =\frac{1}{C_0n}\left[ \sum _{x\in \mathcal {T}^{\scriptscriptstyle (n)}_{v}}\phi (w^{\scriptscriptstyle (n)}(v,x))\left[ |\mathcal {T}^{\scriptscriptstyle (n)}_{t_2}\cap \mathcal {R}^{\scriptscriptstyle (n)}_x|-|\mathcal {T}^{\scriptscriptstyle (n)}_{t_1}\cap \mathcal {R}^{\scriptscriptstyle (n)}_x|\right] .\right] \end{aligned}$$

If $x\in \mathbb {Z}^d/\sqrt{n}$, let $\Delta ^{\scriptscriptstyle (n)}_{x,v}=\mathbb {1}_{\{x\in \mathcal {T}^{\scriptscriptstyle (n)}_{v}\}}(|\mathcal {T}^{\scriptscriptstyle (n)}_{t_2}\cap \mathcal {R}^{\scriptscriptstyle (n)}_x|-|\mathcal {T}^{\scriptscriptstyle (n)}_{t_1}\cap \mathcal {R}^{\scriptscriptstyle (n)}_x|)$. If ${x}_m=(x_1,\dots ,x_m)$, then

3.15 $$\begin{aligned} (H^{\scriptscriptstyle (n)}_{t_2}(\phi ^{v})-H^{\scriptscriptstyle (n)}_{t_1}(\phi ^{v}))^m =\frac{1}{C_0^mn^m}\sum _{{x}_m\in (\mathcal {T}^{\scriptscriptstyle (n)}_{v})^m}\prod _{j=1}^m \phi (w^{\scriptscriptstyle (n)}(v,x_j)) \prod _{j'=1}^m \Delta ^{\scriptscriptstyle (n)}_{x_{j'},v}. \end{aligned}$$

Let $\mathbb {1}_{\{x,v\}}^{\scriptscriptstyle (n)}:=\mathbb {1}_{\{x \in \mathcal {T}^{\scriptscriptstyle (n)}_{v}\}}$ and recall $\gamma >1$ is as in Proposition 3.13.

#### Lemma 3.16

Let $\varepsilon \in (0,1]$, $K>0$ and $T\in \mathbb {N}$. There is a $C_{K,T}>0$ so that for $n\in \mathbb {N}$, $0<p\le 4$, all $x\in \mathbb {Z}^d/\sqrt{n}$, all $t_i\in \mathbb {Z}_{+}/n$ such that $0\le t_1\le t_2\le t_1+1$, and all $0\le v\le t_1-K(t_2-t_1)^\varepsilon$,

3.16 $$\begin{aligned} n\mathbb {E}\Big [|\Delta ^{\scriptscriptstyle (n)}_{x,v}/n|^p \Big |\mathcal {F}_{\ngtr x}\Big ]\le C_{K,T}|t_2-t_1|^{\gamma p/4-\varepsilon }\mathbb {1}_{\{x,v\}}^{\scriptscriptstyle (n)}\quad \text {a.s.} \end{aligned}$$

#### Proof

By Lemma 3.15 with the function $\varphi ^*(R)=\big ||R_{n(t_2-v)}|-|R_{n(t_1-v)}|\big |^p$, the left hand side of (3.16) is at most

$$\begin{aligned}&Cn\rho \mathbb {E}\Big [|H_{t_2-v}^{\scriptscriptstyle (n)}(1)-H_{t_1-v}^{\scriptscriptstyle (n)}(1)|^p\Big ]\mathbb {1}_{\{x,v\}}^{\scriptscriptstyle (n)}\\&\quad =Cn\rho \mathbb {E}\Big [|H_{t_2-v}^{\scriptscriptstyle (n)}(1)-H_{t_1-v}^{\scriptscriptstyle (n)}(1)|^p\Big |H_{t_1-v}^{\scriptscriptstyle (n)}(1)>0\Big ]\mathbb {P}(H_{t_1-v}^{\scriptscriptstyle (n)}(1)>0) \mathbb {1}_{\{x,v\}}^{\scriptscriptstyle (n)}, \end{aligned}$$

where we have used the fact that the integrand is 0 on $\{H_{t_1-v}^{\scriptscriptstyle (n)}(1)=0\}$. By Jensen’s inequality, this is at most

$$\begin{aligned}&Cn\mathbb {P}(H_{t_1-v}^{\scriptscriptstyle (n)}(1)>0) \mathbb {E}\Big [|H_{t_2-v}^{\scriptscriptstyle (n)}(1)-H_{t_1-v}^{\scriptscriptstyle (n)}(1)|^4\Big |H_{t_1-v}^{\scriptscriptstyle (n)}(1)>0\Big ]^{p/4} \mathbb {1}_{\{x,v\}}^{\scriptscriptstyle (n)}\\&\quad \le Cn \mathbb {P}(H_{t_1-v}^{\scriptscriptstyle (n)}(1)>0) \left( n^{-1}\mathbb {P}(H_{t_1-v}^{\scriptscriptstyle (n)}(1)>0)^{-1}n\mathbb {E}\Big [|H_{t_2-v}^{\scriptscriptstyle (n)}(1)-H_{t_1-v}^{\scriptscriptstyle (n)}(1)|^4\Big ]\right) ^{p/4}\mathbb {1}_{\{x,v\}}^{\scriptscriptstyle (n)}\\&\quad \le C_T(n \mathbb {P}(H_{t_1-v}^{\scriptscriptstyle (n)}(1)>0))^{1-p/4}(|t_2-t_1|^{\gamma })^{p/4}\mathbb {1}_{\{x,v\}}^{\scriptscriptstyle (n)}, \end{aligned}$$

where we have used Proposition 3.13, and that, without loss of generality, $t_2-t_1\ge n^{-1}$, so $[t_2-v]_n-[t_1-v]_n\le t_2-t_1+n^{-1}\le 2(t_2-t_1)$. Now use the uniform bound on the survival probability from Condition 3.4 for lattice trees, to bound the above by

$$\begin{aligned}&C(t_1-v)^{p/4-1}|t_2-t_1|^{\gamma p/4}\mathbb {1}_{\{x,v\}}^{\scriptscriptstyle (n)}, \end{aligned}$$

Since $t_1-v\ge K(t_2-t_1)^\varepsilon$ and $|t_2-t_1|\le 1$, this is at most

$$\begin{aligned} C_{K,T} |t_2-t_1|^{\frac{p}{4} (\gamma +\varepsilon )-\varepsilon }\mathbb {1}_{\{x,v\}}\le C_{K,T}|t_2-t_1|^{\frac{p}{4}\gamma -\varepsilon }\mathbb {1}_{\{x,v\}}^{\scriptscriptstyle (n)},\end{aligned}$$

as required. $$\square $$

In proving our next result, we will make use of Lemma 2.13 with each $t_i=t$.

#### Proposition 3.17

There are $\eta ,\varepsilon \in (0,1]$, and for any $T\in \mathbb {N}$ a constant $C_T$, such that for all $\phi \in \textrm{Lip}_1$, all $t_1,t_2\in [0,T]$ satisfying $(2n)^{-1}\le t_2-t_1\le 1/2$ and $v\le t_1-5(t_2-t_1)^\varepsilon$ (*v* may be negative), and all $n\in \mathbb {N}$,

$$\begin{aligned} n\mathbb {E}\Big [(H^{\scriptscriptstyle (n)}_{t_2}(\phi ^{v^+})-H^{\scriptscriptstyle (n)}_{t_1}(\phi ^{v^+}))^4\Big ]\le C_T|t_2-t_1|^{1+\eta }. \end{aligned}$$

#### Proof

We first show that it suffices to prove the above for $t_i\in \mathbb {Z}_{+}/n$ satisfying

3.17 $$\begin{aligned} t_i\le T,\ t_1\le t_2\le t_1+1, \text { and any }v\le t_1-(t_2-t_1)^\varepsilon . \end{aligned}$$

Assume this result and let *n*, $t_i$ and *v* be as in the theorem. Using $t_2-t_1\le 1/2$, we have

$$\begin{aligned} {[}t_2]_n-[t_1]_n\le (t_2-t_1)+\frac{1}{n}\le \frac{1}{2}+\frac{1}{n}<1.\end{aligned}$$

In addition, using $t_2-t_1\ge 1/(2n)$ we have

3.18 $$\begin{aligned} {[}t_2]_n-[t_1]_n\le (t_2-t_1)+\frac{1}{n}\le 3(t_2-t_1),\end{aligned}$$

which implies

$$\begin{aligned} {[}t_1]_n-([t_2]_n-[t_1]_n)^\varepsilon&\ge t_1-\frac{1}{n}-(3(t_2-t_1))^\varepsilon \\&\ge t_1-2(t_2-t_1)-3^\varepsilon (t_2-t_1)^\varepsilon \\&\ge t_1-5(t_2-t_1)^\varepsilon \ge v. \end{aligned}$$

The above inequalities show that our hypotheses (3.17) hold for $[t_i]_n$ and the given *v*. Using the fact that $H^{\scriptscriptstyle (n)}_{t_i}=H^{\scriptscriptstyle (n)}_{[t_i]_n}$ we have from our assumed result, that

$$\begin{aligned} n\mathbb {E}\Big [(H^{\scriptscriptstyle (n)}_{t_2}(\phi ^{v^+})-H^{\scriptscriptstyle (n)}_{t_1}(\phi ^{v^+}))^4\Big ]\le C_T|[t_2]_n-[t_1]_n|^{1+\eta }\le C_T3^{1+\eta }(t_2-t_1)^{1+\eta } \end{aligned}$$

(the last by (3.18)), as required.

So consider now only $t_i\in \mathbb {Z}_{+}/n$ satisfying (3.17) and $t_2>t_1$ (without loss of generality). We first assume $v\le 0$. In this case for all $x\in \mathcal {T}_{t_i}^{\scriptscriptstyle (n)}$, $w^{\scriptscriptstyle (n)}(t_i,x)^0$ is the zero path, $\bar{0}$, and so

$$\begin{aligned} H^{\scriptscriptstyle (n)}_{t_i}(\phi ^{v^+})=H^{\scriptscriptstyle (n)}_{t_i}(\phi ^0)=\phi (\bar{0})H^{\scriptscriptstyle (n)}_{t_i}(1)=\phi (\bar{0})X_{t_i}^{\scriptscriptstyle (n)}(1).\end{aligned}$$

The required inequality now follows (recall $t_i\in \mathbb {Z}_{+}/n$) from Proposition 3.13 and $|\phi (\bar{0})|\le 1$.

So assume henceforth that $0\le v\le t_1-(t_2-t_1)^\varepsilon$. For $x\in \mathcal {T}^{\scriptscriptstyle (n)}_{v}$, and $\phi \in$Lip$_1$, write $\phi _{x,v}^{\scriptscriptstyle (n)}:=\phi (w^{\scriptscriptstyle (n)}(v,x))$. Note that from (3.15) we have

3.19 $$\begin{aligned} D_4&:=n\mathbb {E}\left[ (H^{\scriptscriptstyle (n)}_{t_2}(\phi ^{v})-H^{\scriptscriptstyle (n)}_{t_1}(\phi ^{v}))^4\right] \nonumber \\&=\frac{1}{C_0^4n^3} \mathbb {E}\Bigg [\sum _{\begin{array}{c} {x}=(x_1,x_2,x_3,x_4)\\ \in (\mathbb {Z}^d /\sqrt{n})^4 \end{array}}\prod _{j=1}^4 \Big (\phi ^{\scriptscriptstyle (n)}_{x_j,v}\Delta ^{\scriptscriptstyle (n)}_{x_j,v}\Big )\Bigg ]\nonumber \\&=\frac{1}{C_0^4n^3} \mathbb {E}\Big [\sum _{{x}\in (\mathbb {Z}^d /\sqrt{n})^4 }\phi ^{\scriptscriptstyle (n)}_{{x},v}\Delta ^{\scriptscriptstyle (n)}_{{x},v}\Big ], \end{aligned}$$

where $\Delta ^{\scriptscriptstyle (n)}_{{x},v}$ denotes the product of the indicators $\Delta ^{\scriptscriptstyle (n)}_{x_i,v}$ over the elements $x_i$ of the vector ${x}$ and $\phi ^{\scriptscriptstyle (n)}_{{x},v}$ is the product (running over the elements of the vector ${x}$) of the $\phi ^{\scriptscriptstyle (n)}_{x_i,v}$.

We’d like to condition $\Delta ^{\scriptscriptstyle (n)}_{x_4,v}$ on $\mathcal {F}_{\ngtr x_4}$ in order to extract a positive power of $t_2-t_1$ using Lemma 3.16. This is complicated by the fact that there are terms in the sums where other $x_i=x_4$. If we specify for which *i* this is true for then we will also have a constraint that the remaining $x_j$ are not equal to $x_4$. After conditioning we wish to restore the possibility that these $x_j=x_4$ in order to recover a term of the form $(H^{\scriptscriptstyle (n)}_{t_2}(\phi ^v)-H_{t_1}^{\scriptscriptstyle (n)}(\phi ^v))$ raised to some power smaller than 4 and so derive a recursive inequality which will bound the mean of fourth power of this increment. This results in an inclusion–exclusion argument below. To shorten the notation we will drop the dependence on *v* and *n* in our notation and also suppress the summation range of ${x}$.

In what follows, $A_1\subset [4]$ denotes the set of indices *i* for which $x_i=x_4$ (so in particular $4\in A_1$). Then letting $A_1^c=[4]\setminus A_1$, and writing ${x}(A):=\{x_i:i \in A\}$ and ${x}_A$ for the vector ${x}$ with coordinates restricted to *A*, we have

$$\begin{aligned} D_4=&n^{-3}C_0^{-4}\sum _{\begin{array}{c} A_1\subset [4]:\\ 4 \in A_1 \end{array}}\mathbb {E}\Bigg [\sum _{{x}_{A_1^c}}\phi _{{x}_{A_1^c}}\Delta _{{x}_{A_1^c}} \sum _{x_4\notin {x}(A_1^c)} \phi _{x_4}^{|A_1|}\Delta _{x_4}^{|A_1|}\Bigg ], \end{aligned}$$

where in the case $A_1=[4]$ we interpret the term in the expectation as $\sum _{x_4}\phi _{x_4}^{4}\Delta _{x_4}^{4}$.

Taking conditional expectation with respect to $\mathcal {F}_{\ngtr x_4}$ and using the fact that (for $x_i\ne x_4$), $\mathbb {1}_{\{x_4,v\}}\phi _{x_i}\Delta _{x_i}$ is $\mathcal {F}_{\ngtr x_4}$-measurable (as is $\mathbb {1}_{\{x_4,v\}}\phi _{x_4}$) we have that $D_4$ is equal to

$$\begin{aligned}&n^{-3}C_0^{-4}\sum _{\begin{array}{c} A_1\subset [4]:\\ 4 \in A_1 \end{array}}\mathbb {E}\Bigg [\sum _{{x}_{A_1^c}}\phi _{{x}_{A_1^c}}\Delta _{{x}_{A_1^c}}\sum _{x_4\notin {x}(A_1^c)}\phi _{x_4}^{|A_1|}\mathbb {E}[\Delta _{x_4}^{|A_1|}|\mathcal {F}_{\ngtr x_4}]\Bigg ]. \end{aligned}$$

Interpreting the empty sum $\sum _{x_4\in x(A_1^c)}$ as zero when $A_1^c=\varnothing$, we can write the above as

3.20 $$\begin{aligned}&n^{-3}C_0^{-4}\sum _{\begin{array}{c} A_1\subset [4]:\\ 4 \in A_1 \end{array}}\mathbb {E}\Bigg [\sum _{{x}_{A_1^c}}\phi _{{x}_{A_1^c}}\Delta _{{x}_{A_1^c}}\sum _{x_4}\phi _{x_4}^{|A_1|}\mathbb {E}[\Delta _{x_4}^{|A_1|}|\mathcal {F}_{\ngtr x_4}]\Bigg ] \end{aligned}$$

3.21 $$\begin{aligned}&-n^{-3}C_0^{-4}\sum _{\begin{array}{c} A_1\subset [4]:\\ 4 \in A_1 \end{array}}\mathbb {E}\Bigg [\sum _{{x}_{A_1^c}}\phi _{{x}_{A_1^c}}\Delta _{{x}_{A_1^c}}\sum _{x_4\in {x}(A_1^c)}\phi _{x_4}^{|A_1|}\mathbb {E}[\Delta _{x_4}^{|A_1|}|\mathcal {F}_{\ngtr x_4}]\Bigg ]. \end{aligned}$$

Note that $|A_1|+|A_1^c|=4$ and reason as in (3.19) to see that (3.20) equals

$$\begin{aligned} C\sum _{\begin{array}{c} A_1\subset [4]:\\ 4 \in A_1 \end{array}}\mathbb {E}\Bigg [(H^{\scriptscriptstyle (n)}_{t_2}(\phi ^{v})-H^{\scriptscriptstyle (n)}_{t_1}(\phi ^{v}))^{|A_1^c|}\sum _{x_4}\phi _{x_4}^{|A_1|}n\mathbb {E}[(\Delta _{x_4}/n)^{|A_1|}|\mathcal {F}_{\ngtr x_4}]\Bigg ], \end{aligned}$$

which, by Lemma 3.16 and $|\phi |\le 1$, is bounded in absolute value by

$$\begin{aligned} C\sum _{\begin{array}{c} A_1\subset [4]:\\ 4 \in A_1 \end{array}}\mathbb {E}\Bigg [|H^{\scriptscriptstyle (n)}_{t_2}(\phi ^{v})-H^{\scriptscriptstyle (n)}_{t_1}(\phi ^{v})|^{|A_1^c|}\sum _{x_4}\mathbb {1}_{\{x_4,v\}}\Bigg ]|t_2-t_1|^{(\gamma |A_1|/4)-\varepsilon }. \end{aligned}$$

Expressing the sum over $x_4$ in terms of $H^{\scriptscriptstyle (n)}_{v}(1)$ this is equal to

$$\begin{aligned} C\sum _{\begin{array}{c} A_1\subset [4]:\\ 4 \in A_1 \end{array}}n\mathbb {E}\Bigg [|H^{\scriptscriptstyle (n)}_{t_2}(\phi ^{v})-H^{\scriptscriptstyle (n)}_{t_1}(\phi ^{v})|^{|A_1^c|}H^{\scriptscriptstyle (n)}_{v}(1)\Bigg ]|t_2-t_1|^{(\gamma |A_1|/4)-\varepsilon }. \end{aligned}$$

By Hölder’s inequality this is at most

$$\begin{aligned}&C\sum _{\begin{array}{c} A_1\subset [4]:\\ 4 \in A_1 \end{array}}n\mathbb {E}\Big [|H^{\scriptscriptstyle (n)}_{t_2}(\phi ^{v})-H^{\scriptscriptstyle (n)}_{t_1}(\phi ^{v})|^4\Big ]^{|A_1^c|/4}\mathbb {E}\Big [H^{\scriptscriptstyle (n)}_{v}(1)^{4/(4-|A_1^c|)}\Big ]^{(4-|A_1^c|)/4} |t_2-t_1|^{(\gamma |A_1|/4)-\varepsilon }\\&\quad \le C\sum _{\begin{array}{c} A_1\subset [4]:\\ 4 \in A_1 \end{array}}D_4^{|A_1^c|/4}|t_2-t_1|^{\gamma |A_1|/4-\varepsilon }\Big (n\mathbb {E}\Big [H^{\scriptscriptstyle (n)}_{v}(1)^{4/(4-|A_1^c|)}\Big ]\Big )^{(4-|A_1^c|)/4}. \end{aligned}$$

Note that for $b\le 3$ we have that for $H\ge 0$, $H^{4/(4-b)}\le H +H^4$. Since $n\mathbb {E}[(H_v^{\scriptscriptstyle (n)}(1))^r]<C_{r,T}$ for each $r\in \mathbb {N}$ (by Lemma 2.13), this shows that this quantity is at most (*C* may depend on *T* throughout)

$$\begin{aligned} C\sum _{\begin{array}{c} A_1\subset [4]:\\ 4 \in A_1 \end{array}}D_4^{|A_1^c|/4}|t_2-t_1|^{(\gamma |A_1|/4)-\varepsilon }. \end{aligned}$$

We turn now to the quantity (3.21), and it is convenient to introduce further notation. For sets $A_i\subset [4]$, let $B_i=\cup _{j=1}^iA_j$. In particular $B_1=A_1$. Thus (3.21) is equal to the negative of

3.22 $$\begin{aligned}&n^{-3}C_0^{-4}\sum _{\begin{array}{c} A_1\subset [4]:\\ 4 \in A_1 \end{array}}\mathbb {E}\Bigg [\sum _{{x}_{B_1^c}}\phi _{{x}_{B_1^c}}\Delta _{{x}_{B_1^c}} \sum _{x_4}\mathbb {1}_{\{x_4\in {x}({B_1^c})\}} \phi _{x_4}^{|A_1|}\mathbb {E}[\Delta _{x_4}^{|A_1|}|\mathcal {F}_{\ngtr x_4}]\Bigg ]. \end{aligned}$$

Abusing notation by writing $x(A)=x$ to mean that $x_i=x$ for each $i \in A$ we can write

$$\begin{aligned} \mathbb {1}_{\{x_4\in {x}(B_1^c)\}}=\sum _{\begin{array}{c} A_2\subset B_1^c:\\ A_2\ne \varnothing \end{array}}\mathbb {1}_{\{x(A_2)=x_4\}}\mathbb {1}_{\{x_4\notin {x}(B_2^c)\}},\end{aligned}$$

which is simply the statement that $x_4\in x(B_1^c)$ if and only if the set $A_2:=\{i \in [4]\setminus B_1:x_4=x_i\}$ is non-empty. Thus, since $x_i=x_4$ for $i\in A_2$ in this expression, (3.22) is equal to

3.23 $$\begin{aligned}&n^{-3}C_0^{-4}\sum _{\begin{array}{c} A_1\subset [4]:\\ 4 \in A_1 \end{array}}\sum _{\begin{array}{c} A_2\subset B_1^c:\\ A_2\ne \varnothing \end{array}}\mathbb {E}\Bigg [\sum _{{x}_{B_2^c}}\phi _{{x}_{B_2^c}}\Delta _{{x}_{B_2^c}} \sum _{x_4} \mathbb {1}_{\{x_4\notin {x}(B_2^c)\}} \phi _{x_4}^{|B_2|}\Delta _{x_4}^{|A_2|}\mathbb {E}[\Delta _{x_4}^{|A_1|}|\mathcal {F}_{\ngtr x_4}]\Bigg ], \end{aligned}$$

where we have also used the fact that $\phi _{{x}_{B_1^c}}=\phi _{{x}_{B_2^c}}\phi _{{x}_{A_2}}=\phi _{{x}_{B_2^c}}\phi _{x_4}^{|A_2|}$, and $|A_2|+|A_1|=|B_2|$. In the case $B_2^c=\varnothing$ the term in the expectation in (3.23) should be interpreted as $\sum _{x_4}\phi _{x_4}^{4}\Delta _{x_4}^{|A_2|}\mathbb {E}[\Delta _{x_4}^{|A_1|}|\mathcal {F}_{\ngtr x_4}]$.

We can again condition on $\mathcal {F}_{\ngtr x_4}$ to see that (3.23) is equal to

$$\begin{aligned}&n^{-3}C_0^{-4}\sum _{\begin{array}{c} A_1\subset [4]:\\ 4 \in A_1 \end{array}}\sum _{\begin{array}{c} A_2\subset B_1^c:\\ A_2\ne \varnothing \end{array}}\mathbb {E}\Bigg [\sum _{{x}_{B_2^c}}\phi _{{x}_{B_2^c}}\Delta _{{x}_{B_2^c}} \sum _{x_4 \notin {x}(B_2^c )} \phi _{x_4}^{|B_2|} \prod _{i=1}^2\mathbb {E}[\Delta _{x_4}^{|A_i|}|\mathcal {F}_{\ngtr x_4}]\Bigg ]. \end{aligned}$$

Using inclusion–exclusion in the sum over $x_4$ this can be written as

3.24 $$\begin{aligned}&n^{-3}C_0^{-4}\sum _{\begin{array}{c} A_1\subset [4]:\\ 4 \in A_1 \end{array}}\sum _{\begin{array}{c} A_2\subset B_1^c:\\ A_2\ne \varnothing \end{array}} \mathbb {E}\Bigg [\sum _{{x}_{B_2^c}}\phi _{{x}_{B_2^c}}\Delta _{{x}_{B_2^c}} \sum _{x_4} \phi _{x_4}^{|B_2|}\prod _{i=1}^2\mathbb {E}[\Delta _{x_4}^{|A_i|}|\mathcal {F}_{\ngtr x_4}]\Bigg ] \end{aligned}$$

3.25 $$\begin{aligned}&-n^{-3}C_0^{-4}\sum _{\begin{array}{c} A_1\subset [4]:\\ 4 \in A_1 \end{array}}\sum _{\begin{array}{c} A_2\subset B_1^c:\\ A_2\ne \varnothing \end{array}}\mathbb {E}\Bigg [\sum _{{x}_{B_2^c}}\phi _{{x}_{B_2^c}}\Delta _{{x}_{B_2^c}} \sum _{x_4\in {x}(B_2^c )} \phi _{x_4}^{|B_2|}\prod _{i=1}^2\mathbb {E}[\Delta _{x_4}^{|A_i|}|\mathcal {F}_{\ngtr x_4}]\Bigg ], \end{aligned}$$

where the sum over $x_4$ in (3.25) is interpreted as 0 when $B_2^c=\varnothing$. The quantity (3.24) is equal to [reasoning as in (3.19)]

$$\begin{aligned} \sum _{\begin{array}{c} A_1\subset [4]:\\ 4 \in A_1 \end{array}}\sum _{\begin{array}{c} A_2\subset B_1^c:\\ A_2\ne \varnothing \end{array}}\frac{C}{n}\mathbb {E}\Bigg [&(H^{\scriptscriptstyle (n)}_{t_2}(\phi ^{v})-H^{\scriptscriptstyle (n)}_{t_1}(\phi ^{v}))^{|B_2^c|} \sum _{x_4} \phi _{x_4}^{|B_2|}\prod _{i=1}^2\ n\mathbb {E}[(\Delta _{x_4}/n)^{|A_i|}|\mathcal {F}_{\ngtr x_4}]\Bigg ]. \end{aligned}$$

We have also used $|B_2^c|+|A_1|+|A_2|=4$ to get the correct powers of *n*. Using Lemma 3.16 again as before, we may bound the summand (in absolute value) by

3.26 $$\begin{aligned}&C\mathbb {E}\Big [|H^{\scriptscriptstyle (n)}_{t_2}(\phi ^{v})-H^{\scriptscriptstyle (n)}_{t_1}(\phi ^{v})|^{|B_2^c|} H^{\scriptscriptstyle (n)}_{v}(1)\Big ]\prod _{i=1}^2 |t_2-t_1|^{(\gamma |A_i|/4)-\varepsilon } \end{aligned}$$

3.27 $$\begin{aligned}&\le n^{-1}|t_2-t_1|^{(\gamma |B_2|/4)-2\varepsilon }D_4^{|B_2^c|/4}, \end{aligned}$$

where we have again used Hölder’s inequality, Lemma 2.13, and $\sum _{i=1}^2|A_i|=|B_2|$ since $A_1$ and $A_2$ are disjoint. As in (3.23), the negative of (3.25) is equal to

$$\begin{aligned}&\frac{C_0^{-4}}{n^{3}}\sum _{\begin{array}{c} A_1\subset [4]:\\ 4 \in A_1 \end{array}}\sum _{\begin{array}{c} A_2\subset B_1^c:\\ A_2\ne \varnothing \end{array}}\sum _{\begin{array}{c} A_3\subset B_2^c:\\ A_3\ne \varnothing \end{array}}\mathbb {E}\Bigg [\sum _{{x}_{B_3^c}}\phi _{{x}_{B_3^c}}\Delta _{{x}_{B_3^c}} \sum _{x_4\notin {x}(B_3^c)} \phi _{x_4}^{|B_3|}\Delta _{x_4}^{|A_3|} \prod _{i=1}^2\mathbb {E}[\Delta _{x_4}^{|A_i|}|\mathcal {F}_{\ngtr x_4}]\Bigg ], \end{aligned}$$

where if $B_3^c=\varnothing$ the term in the expectation is interpreted as $\sum _{x_4} \phi _{x_4}^{4}\Delta _{x_4}^{|A_3|} \prod _{i=1}^2\mathbb {E}[\Delta _{x_4}^{|A_i|}|\mathcal {F}_{\ngtr x_4}]$. Conditioning again, this is equal to

3.28 $$\begin{aligned}&\frac{C_0^{-4}}{n^{3}}\sum _{\begin{array}{c} A_1\subset [4]:\\ 4 \in A_1 \end{array}}\sum _{\begin{array}{c} A_2\subset B_1^c:\\ A_2\ne \varnothing \end{array}}\sum _{\begin{array}{c} A_3\subset B_2^c:\\ A_3\ne \varnothing \end{array}}\mathbb {E}\Bigg [\sum _{{x}_{B_3^c}}\phi _{{x}_{B_3^c}}\Delta _{{x}_{B_3^c}} \sum _{x_4 \notin {x}(B_3^c )} \phi _{x_4}^{|B_3|}\prod _{i=1}^3\mathbb {E}[\Delta _{x_4}^{|A_i|}|\mathcal {F}_{\ngtr x_4}]\Bigg ]\nonumber \\&\quad =\frac{C_0^{-4}}{n^{3}}\sum _{\begin{array}{c} A_1\subset [4]:\\ 4 \in A_1 \end{array}}\sum _{\begin{array}{c} A_2\subset B_1^c:\\ A_2\ne \varnothing \end{array}}\sum _{\begin{array}{c} A_3\subset B_2^c:\\ A_3\ne \varnothing \end{array}}\mathbb {E}\Bigg [\sum _{{x}_{B_3^c}}\phi _{{x}_{B_3^c}}\Delta _{{x}_{B_3^c}} \sum _{x_4} \phi _{x_4}^{|B_3|}\prod _{i=1}^3\mathbb {E}[\Delta _{x_4}^{|A_i|}|\mathcal {F}_{\ngtr x_4}]\Bigg ] \end{aligned}$$

3.29 $$\begin{aligned}&\qquad -\frac{C_0^{-4}}{n^{3}}\sum _{\begin{array}{c} A_1\subset [4]:\\ 4 \in A_1 \end{array}}\sum _{\begin{array}{c} A_2\subset B_1^c:\\ A_2\ne \varnothing \end{array}}\sum _{\begin{array}{c} A_3\subset B_2^c:\\ A_3\ne \varnothing \end{array}}\mathbb {E}\Bigg [\sum _{{x}_{B_3^c}}\phi _{{x}_{B_3^c}}\Delta _{{x}_{B_3^c}} \sum _{x_4\in {x}(B_3^c )} \phi _{x_4}^{|B_3|}\prod _{i=1}^3\mathbb {E}[\Delta _{x_4}^{|A_i|}|\mathcal {F}_{\ngtr x_4}]\Bigg ]. \end{aligned}$$

As in (3.26) and (3.27), the term (3.28) is bounded in absolute value by

$$\begin{aligned}&\frac{C}{n^2}\sum _{\begin{array}{c} A_1\subset [4]:\\ 4 \in A_1 \end{array}}\sum _{\begin{array}{c} A_2\subset B_1^c:\\ A_2\ne \varnothing \end{array}}\sum _{\begin{array}{c} A_3\subset B_2^c:\\ A_3\ne \varnothing \end{array}}n\mathbb {E}\Bigg [|H^{\scriptscriptstyle (n)}_{t_2}(\phi ^{v})-H^{\scriptscriptstyle (n)}_{t_1}(\phi ^{v})|^{|B_3^c|}H^{\scriptscriptstyle (n)}_{v}(1)\Bigg ] \prod _{i=1}^3 |t_2-t_1|^{\gamma |A_i|/4-\varepsilon }\\&\quad \le \frac{C}{n^2}\sum _{\begin{array}{c} A_1\subset [4]:\\ 4 \in A_1 \end{array}}\sum _{\begin{array}{c} A_2\subset B_1^c:\\ A_2\ne \varnothing \end{array}}\sum _{\begin{array}{c} A_3\subset B_2^c:\\ A_3\ne \varnothing \end{array}}D_4^{|B_3^c|/4}|t_2-t_1|^{\gamma |B_3|/4-3\varepsilon }. \end{aligned}$$

Since in (3.29) $B_3^c$ can contain at most one element, the sums over ${x}_{B_3^c}$ and $x_4\in x(B_3^c)$ therein reduce to a sum over $x_4$ (with ${x}_{B_3^c}=x_4$). After conditioning again we get that the negative of (3.29) is equal to

3.30 $$\begin{aligned} \frac{C_0^{-4}}{n^{3}}\sum _{\begin{array}{c} A_1\subset [4]:\\ 4 \in A_1 \end{array}}\sum _{\begin{array}{c} A_2\subset B_1^c:\\ A_2\ne \varnothing \end{array}}\sum _{\begin{array}{c} A_3\subset B_2^c:\\ A_3\ne \varnothing \end{array}}\sum _{\begin{array}{c} A_4\subset B_3^c:\\ A_4\ne \varnothing \end{array}} \mathbb {E}\Bigg [ \sum _{x_4} \phi _{x_4}^{|B_4|}\prod _{i=1}^4\mathbb {E}[\Delta _{x_4}^{|A_i|}|\mathcal {F}_{\ngtr x_4}]\Bigg ], \end{aligned}$$

where we note that if this term is to be non-zero then each $|A_i|=1$, and in particular $B_4=[4]$. By Lemma 3.16 and then Lemma 2.13, (3.30) is bounded in absolute value by

$$\begin{aligned}&\frac{C}{n^{3}}\sum _{\begin{array}{c} A_1\subset [4]:\\ 4 \in A_1 \end{array}}\sum _{\begin{array}{c} A_2\subset B_1^c:\\ A_2\ne \varnothing \end{array}}\sum _{\begin{array}{c} A_3\subset B_2^c:\\ A_3\ne \varnothing \end{array}}\sum _{\begin{array}{c} A_4\subset B_3^c:\\ A_4\ne \varnothing \end{array}}\mathbb {E}\Big [ \sum _{x_4}\mathbb {1}_{\{x_4,v\}}\Big ]\prod _{i=1}^4|t_2-t_1|^{\gamma |A_i|/4-\varepsilon }\\&\quad \le \frac{C}{n^{3}}\sum _{\begin{array}{c} A_1\subset [4]:\\ 4 \in A_1 \end{array}}\sum _{\begin{array}{c} A_2\subset B_1^c:\\ A_2\ne \varnothing \end{array}}\sum _{\begin{array}{c} A_3\subset B_2^c:\\ A_3\ne \varnothing \end{array}}\sum _{\begin{array}{c} A_4\subset B_3^c:\\ A_4\ne \varnothing \end{array}}|t_2-t_1|^{\gamma -4\varepsilon }. \end{aligned}$$

After dropping some negative powers of *n*, we have shown above that

$$\begin{aligned} D_4\le C'\sum _{\ell =0}^3 D_4^{\ell /4}|t_2-t_1|^{\frac{\gamma }{4}(4-\ell )-4\varepsilon }, \end{aligned}$$

Thus, letting $d=D_4 |t_2-t_1|^{16\varepsilon -\gamma }$ and recalling that $|t_2-t_1|\le 1$, we have

$$\begin{aligned} d\le C'\sum _{\ell =0}^3 d^{\ell /4}. \end{aligned}$$

Recall that $D_4$ is finite by Lemma 2.13, and so from the above, $d\le C=C(C')$, and therefore $D_4\le C|t_2-t_1|^{\gamma -16\varepsilon }$. Choosing $\varepsilon <(\gamma -1)/16$ completes the proof. $\square$

For $v<0$ define $\phi ^v=\phi ^0$ so that $\phi ^v=\phi ^{v^+}$.

#### Proof of Proposition 3.11

Let $\phi \in \textrm{Lip}_1$ and $n_k\rightarrow \infty$. For a fixed $s_0>0$ we must show that $\{n_k\}$ has a subsequence $\{n'_k\}$ along which $\mathbb {P}\big (H_\cdot ^{\scriptscriptstyle (n'_k)}(\phi )\in \cdot |S^{\scriptscriptstyle (n'_k)}>s_0\big )$ converges weakly to a continuous limit. The argument remains unchanged if we assume $n_k=k$, and to ease the notation we will assume this. So our goal is to show that

3.31 $$\begin{aligned}&\{\mathbb {P}(H^{\scriptscriptstyle (n)}_{\cdot }(\phi )\in \cdot )|S^{\scriptscriptstyle (n)}>s_0):n\in \mathbb {N}\}\text { has a weakly convergent}\nonumber \\&\quad \text {subsequence in }\mathcal {D}(\mathbb {R}_+,\mathbb {R})\text { to a continuous limit}. \end{aligned}$$

For $$T\in \mathbb {N}$$, define

$$\begin{aligned} {X_T^{\scriptscriptstyle (n)}}^*(1)=\sup _{t\le T}X^{\scriptscriptstyle (n)}_t(1). \end{aligned}$$

Now fix $T\in \mathbb {N}$ and assume

3.32 $$\begin{aligned} t_1,t_2\in [0,T],\ 0\le t_2-t_1\le 1 \qquad \text { and }\qquad t_1-5(t_2-t_1)^\varepsilon \le v\le t_1, \end{aligned}$$

where $\varepsilon$ is as in Proposition 3.17; note that *v* may be negative. Recall from (3.14) that

3.33 $$\begin{aligned} |H^{\scriptscriptstyle (n)}_{t_2}(\phi )-H^{\scriptscriptstyle (n)}_{t_1}(\phi )|&\le \Bigl [ \sum _{i=1}^2|H^{\scriptscriptstyle (n)}_{t_i}(\phi )-H^{\scriptscriptstyle (n)}_{t_i}(\phi ^{v})|\Bigr ]+|H^{\scriptscriptstyle (n)}_{t_2}(\phi ^{v})-H^{\scriptscriptstyle (n)}_{t_1}(\phi ^{v})|. \end{aligned}$$

Note that (3.32) implies $t_2-v^+\le t_2-v\le (t_2-t_1)+5(t_2-t_1)^\varepsilon$, and so if $\delta _n(\omega )$ is as in Lemma 3.12, then Lemma 3.14 (applied to $v^+\ge 0$) together with the facts that $\phi ^v=\phi ^{v^+}$ and $t_2-t_1\le 1$ show that

3.34 $$\begin{aligned}&(t_2-t_1)+5(t_2-t_1)^\varepsilon \le \delta _n\text { implies } \nonumber \\&\quad \sum _{i=1}^2|H^{\scriptscriptstyle (n)}_{t_i}(\phi )-H^{\scriptscriptstyle (n)}_{t_i}(\phi ^{v})| \le C{X_T^{\scriptscriptstyle (n)}}^*(1)[(t_2-t_1)^{\varepsilon /4}+n^{-1/4}]. \end{aligned}$$

If $\eta >0$ is as in Proposition 3.17, let $\eta _0=\eta /8$. Proposition 3.17 shows that for $m,n\in \mathbb {N}$ satisfying

$$\begin{aligned} m\le (\log _2n)+1, \quad \text { that is, }\quad 2^{-m}\ge \frac{1}{2n}, \end{aligned}$$

then, by taking a union bound over $k\in \mathbb {Z}_+:0\le k2^{-m}\le T+1$,

$$\begin{aligned}&n\mathbb {P}\Big (\max _{0\le k\le 2^m(T+1)}\big |H^{\scriptscriptstyle (n)}_{(k+1)2^{-m}}(\phi ^{(k2^{-m}-5\cdot 2^{-m\varepsilon })^+})-H^{\scriptscriptstyle (n)}_{k2^{-m}}(\phi ^{(k2^{-m}-5\cdot 2^{-m\varepsilon })^+})\big |>2^{-m\eta _0}\Big )\\&\quad \le 2^{4m\eta _0}(T+2)2^mC_{T+2}2^{-m(1+\eta )}=C'_T2^{-m(\eta /2)}. \end{aligned}$$

By a union bound there is an $M_0^{\scriptscriptstyle (n)}(\omega )\in \mathbb {N}^{\ge 2}$ so that

3.35 $$\begin{aligned} \text {for all }M\ge 2,\ n\mathbb {P}(M_0^{\scriptscriptstyle (n)}\ge M)\le C_{T,\eta }2^{-M\eta /2}, \end{aligned}$$

and for all $m\in \mathbb {N}$ satisfying $M_0^{\scriptscriptstyle (n)}\le m\le (\log _2n)+1$, we have

$$\begin{aligned} \max _{0\le k\le 2^m(T+1)}\big |H^{\scriptscriptstyle (n)}_{(k+1)2^{-m}}(\phi ^{(k2^{-m}-5\cdot 2^{-m\varepsilon })^+})-H^{\scriptscriptstyle (n)}_{k2^{-m}}(\phi ^{(k2^{-m}-5\cdot 2^{-m\varepsilon })^+})\big |\le 2^{-m\eta _0}. \end{aligned}$$

Set $\eta _1=(\varepsilon /4)\wedge \eta _0>0$. Combine the above bound with (3.34) and use it in (3.33) (with $T+1$ in place of *T* in the latter two) to see that for all natural numbers *m* satisfying

$$\begin{aligned} \frac{1}{2n}\le 2^{-m}\le 2^{-M_0^{\scriptscriptstyle (n)}}\text { and }6\cdot 2^{-m\varepsilon }\le \delta _n,\end{aligned}$$

we have

3.36 $$\begin{aligned} \max _{0\le k\le 2^m(T+1)}|H^{\scriptscriptstyle (n)}_{(k+1)2^{-m}}(\phi )-H^{\scriptscriptstyle (n)}_{k2^{-m}}(\phi )|&\le 2C{{X_{T+2}^{\scriptscriptstyle (n)}}^*(1)}(2^{-m\varepsilon /4}+n^{-1/4})+2^{-m\eta _0}\nonumber \\&\le (6C{X_{T+2}^{\scriptscriptstyle (n)}}^*(1)+1)2^{-m\eta _1}. \end{aligned}$$

Set $m_n=\lfloor (\log _2n)+1\rfloor$ and $T_n=\{j2^{-m_n}:j\in \mathbb {Z}_+\cap [0,(T+1)2^{m_n}]\}$. Lévy’s binary expansion argument and (3.36) shows that if

$$\begin{aligned} t_1, t_2\in T_n, \text { and }0\le t_2-t_1<2^{-M^{\scriptscriptstyle (n)}_0}\wedge (\delta _n/6)^{1/\varepsilon },\end{aligned}$$

then

3.37 $$\begin{aligned} |H^{\scriptscriptstyle (n)}_{t_2}(\phi )-H^{\scriptscriptstyle (n)}_{t_1}(\phi )|\le C( {X_{T+2}^{\scriptscriptstyle (n)}}^*(1) +1)|t_2-t_1|^{\eta _1}. \end{aligned}$$

Since $\frac{1}{2^{m_n}}<\frac{1}{n}$, for any $t\in [0,T]$ we may choose $\{t\}_n\in \big [[t]_n,[t]_n+\frac{1}{n}\big )\cap T_n$. Let

$$\begin{aligned} \delta _n'=\frac{1}{3}(2^{-M_0^{\scriptscriptstyle (n)}}\wedge (\delta _n/6)^{1/\varepsilon }). \end{aligned}$$

Let $t_1,t_2\in [0,T]\cap \mathbb {Z}_{+}/n$ be such that $0<t_2-t_1\le \delta _n'$. Then $|t_i-\{t_i\}_n|<1/n\le |t_2-t_1|$, which implies that

$$\begin{aligned} |\{t_2\}_n-\{t_1\}_n|\le |t_2-t_1|+\frac{2}{n}<3|t_2-t_1|\le 2^{-M_0^{\scriptscriptstyle (n)}}\wedge (\delta _n/6)^{1/\varepsilon }. \end{aligned}$$

Thus (3.37) holds for $\{t_2\}_n,\{t_1\}_n$, that is,

$$\begin{aligned} |H^{\scriptscriptstyle (n)}_{\{t_2\}_n}(\phi )-H^{\scriptscriptstyle (n)}_{\{t_1\}_n}(\phi )|&\le C( {X_{T+2}^{\scriptscriptstyle (n)}}^*(1) +1)|\{t_2\}_n-\{t_1\}_n|^{\eta _1}\\&\le C3^{\eta _1}( {X_{T+2}^{\scriptscriptstyle (n)}}^*(1) +1)|t_2-t_1|^{\eta _1}. \end{aligned}$$

Now use the fact that $H^{\scriptscriptstyle (n)}_{t_i}(\phi )=H^{\scriptscriptstyle (n)}_{\{t_i\}_n}(\phi )$ for $i=1,2$ to conclude that:

3.38 $$\begin{aligned}&\text {for all }t_1<t_2\in [0,T]\cap (\mathbb {Z}_{+}/n)\text { such that }t_2-t_1\le \delta _n'\text { we have } \end{aligned}$$

3.39 $$\begin{aligned}&|H^{\scriptscriptstyle (n)}_{t_2}(\phi )-H^{\scriptscriptstyle (n)}_{t_1}(\phi )|\le C_T({X_{T+2}^{\scriptscriptstyle (n)}}^*(1)+1)|t_2-t_1|^{\eta _1}. \end{aligned}$$

Next use Lemma 3.12 and (3.35) to see that for $r\in (0,\frac{1}{12})$, then

3.40 $$\begin{aligned} n\mathbb {P}(\delta _n'\le r)&\le n\mathbb {P}(M_0^{\scriptscriptstyle (n)}\ge \log _2(1/3r))+n\mathbb {P}(\delta _n\le 6(3r)^\varepsilon )\nonumber \\&\le C_{T,\eta }(3r)^{\eta /2}+c6(3r)^\varepsilon \nonumber \\&\le C'_{T,\eta }r^{\varepsilon \wedge (\eta /2)}\le C'_{T,\eta }r^{\eta _1}. \end{aligned}$$

Our objective now follows easily from (3.39) and (3.40). Let $\{{\tilde{H}}^{\scriptscriptstyle (n)}_{t},\ t\ge 0\}$ be the continuous process obtained by linearly interpolating $\{H^{\scriptscriptstyle (n)}_{j/n}(\phi ):j\in \mathbb {Z}_+\}$. It follows from (3.38) and (3.39), with $T+1$ in place of *T*, that for some $C'_T$,

3.41 $$\begin{aligned}&\text {if }t_1\le t_2\in [0,T]\text { and } (t_2-t_1)\vee \frac{1}{n}\le \delta '_n,\text { then }\nonumber \\&\quad |{\tilde{H}}^{\scriptscriptstyle (n)}_{t_2}-{\tilde{H}}^{\scriptscriptstyle (n)}_{t_1}|\le C'_T({X^{\scriptscriptstyle (n)}_{T+3}}^*(1)+1)|t_2-t_1|^{\eta _1}. \end{aligned}$$

For $t_2-t_1\ge \frac{1}{n}$ this is an easy consequence of the triangle inequality and the fact that $\delta '_n\ge \frac{1}{n}$. For $0<t_2-t_1<\frac{1}{n}$, either $[t_1]_n=[t_2]_n$ and the linear interpolation and $\delta _n'\ge 1/n$ easily give the desired bound, or $[t_2]_n=[t_1]_n+1/n$, and the triangle inequality gives

$$\begin{aligned} |{\tilde{H}}^{\scriptscriptstyle (n)}_{t_2}-{\tilde{H}}^{\scriptscriptstyle (n)}_{t_1}|&\le |{\tilde{H}}^{\scriptscriptstyle (n)}_{t_2}-{\tilde{H}}^{\scriptscriptstyle (n)}_{[t_2]_n}|+|{\tilde{H}}^{\scriptscriptstyle (n)}_{[t_2]_n}-{\tilde{H}}^{\scriptscriptstyle (n)}_{t_1}| \end{aligned}$$

which leads to the required bound using the linear interpolation and $\delta '_n\ge 1/n$ again.

Recall that $|\phi |\le 1$ implies $|\tilde{H}_0^{\scriptscriptstyle (n)}|\le \frac{1}{C_0 n}$. We now fix $T\in \mathbb {N}$, and for $\delta ,M>0$, define a compact set of paths in $\mathcal {C}=\mathcal {C}([0,T],\mathbb {R})$ by

$$\begin{aligned} \begin{aligned} K_{\delta ,M}=\big \{w\in \mathcal {C}:&\,{|w_0|}\le C_0^{-1} \text{ and }\ \forall t_1,t_2\in [0,T],\ \text{ if } |t_2-t_1|\le \delta \text{ then } \\ {}&\quad |w_{t_2}-w_{t_1}|\le C'_T(M+1)|t_2-t_1|^{\eta _1}\big \}. \end{aligned} \end{aligned}$$

Compactness is clear by the Arzela–Ascoli Theorem. Recall that $s_0>0$. It follows from (3.41) and (3.40) that for small enough $\delta _k>0$ and large enough $M_k, n_k\in \mathbb {N}$,

3.42 $$\begin{aligned} n\mathbb {P}({\tilde{H}}^{\scriptscriptstyle (n)}_{\cdot }|_{[0,T]}\notin K_{\delta ,M}, S^{\scriptscriptstyle (n)}>s_0)&\le n\mathbb {P}\big ({X^{\scriptscriptstyle (n)}_{T+3}}^*(1)>M, S^{\scriptscriptstyle (n)}>s_0\big )+n\mathbb {P}\big (\delta '_n\le \frac{1}{n}\vee \delta \big )\nonumber \\&\le 2^{-k}, \quad \text {if }\delta \le \delta _k,\ M\ge M_k,\text { and }n\ge n_k. \end{aligned}$$

Here we are using the tightness of the maximum total mass processes from [11, Theorem 1.2 and Corollary 1.3]. By further decreasing $\delta _k$ and increasing $M_k$ we can realize the bound in (3.42) for all $n\in \mathbb {N}$. It now follows that for the compact sets $\hat{K}_m=\cap _{k=m}^\infty K_{\delta _k,M_k}$ we have

3.43 $$\begin{aligned} \text {for all }m,n\in \mathbb {N},\ \ n\mathbb {P}\big ({\tilde{H}}^{\scriptscriptstyle (n)}_{ }|_{[0,T]}\notin \hat{K}_m, S^{\scriptscriptstyle (n)}>s_0\big )\le 2^{1-m}. \end{aligned}$$

We use the lower bound on the survival probability from (3.8):

$$\begin{aligned} \mathbb {P}(S^{\scriptscriptstyle (n)}>s_0)\ge \underline{c}((ns_0)\vee 1)^{-1}. \end{aligned}$$

Combine the above with (3.43) to conclude that for all $m,n\in \mathbb {N}$,

$$\begin{aligned} \mathbb {P}\big ({\tilde{H}}^{\scriptscriptstyle (n)}_{}|_{[0,T]}\notin \hat{K}_m|S^{\scriptscriptstyle (n)}>s_0\big )&\le \frac{\mathbb {P}({\tilde{H}}^{\scriptscriptstyle (n)}_{}|_{[0,T]}\notin \hat{K}_m, S^{\scriptscriptstyle (n)}>s_0)}{\underline{c}/((ns_0)\vee 1)}\\&\le \underline{c}^{-1}(ns_0+1)\mathbb {P}({\tilde{H}}^{\scriptscriptstyle (n)}_{}|_{[0,T]}\notin \hat{K}_m, S^{\scriptscriptstyle (n)}>s_0)\\&\le \underline{c}^{-1}(s_0+1)2^{1-m}. \end{aligned}$$

This shows that $\{\mathbb {P}({\tilde{H}}^{\scriptscriptstyle (n)}_{}\in \cdot |S^{\scriptscriptstyle (n)}>s_0):n\in \mathbb {N}\}$ is tight in $\mathcal {C}(\mathbb {R}_+,\mathbb {R})$ and so by Prohorov’s theorem is relatively compact in $\mathcal {C}(\mathbb {R}_+,\mathbb {R})$. This implies (see, e.g., [8, Proposition 10.4 in Chapter 3]) that $\{\mathbb {P}(H^{\scriptscriptstyle (n)}_{}(\phi )\in \cdot |S^{\scriptscriptstyle (n)}>s_0):n\in \mathbb {N}\}$ is $\mathcal {C}$-relatively compact in $\mathcal {D}(\mathbb {R}_+,\mathbb {R})$, proving (3.31), as required. $\square$

## Proof of Proposition 2.11

The goal of this section is to prove Proposition 2.11. The proof is a modification of that of [17, Theorem 4.8], so we will not give all of the details here. Instead we will indicate the main ideas of the proof, and refer the reader to [17] for various details.

For ${\digamma }\in \Sigma _r$, [17, Theorem 4.8] proves Proposition 2.11 in the simplified setting where $j_e=1$ for every $e\in \mathcal {E}({\digamma })$. In that reference (and with $j_e=1$ for each *e*) the quantity $\hat{t}^{({\digamma })}_{\check{\varvec{n}}}(\cdot )$ is written as $\hat{t}_{\mathcal {N}({\digamma },\check{\varvec{n}})}(\cdot )$, where $\mathcal {N}({\digamma },\check{\varvec{n}})$ denotes a skeleton network consisting of inserting $\check{n}_{e,1}-1$ vertices into edge *e*, for each $e \in \mathcal {E}({\digamma })$. The quantity $\rho ^{-1}\hat{t}_{\mathcal {N}({\digamma },\check{\varvec{n}})}$ then encodes (in Fourier space) the probability of our random tree $\mathcal {T}$ connecting the origin to *r* specified space–time points with the spatial and temporal locations of the branch points, as well as the “shape” of the connections also specified (consider the set $\check{\varvec{T}}({\digamma },\check{\varvec{y}},\check{\varvec{n}})$ in the case where each $j_e=1$). In our paper $j_e$ need not be equal to 1. In this more general setting, $\hat{t}^{({\digamma })}_{\check{\varvec{n}}}(\cdot )$ encodes (in Fourier space) the probability of a subset of the above event, where now the spatial locations at various other fixed times are also specified. The appropriate skeleton network is now a *marked skeleton network* $\mathcal {N}^+$ (see below), where certain vertices on the skeleton network $\mathcal {N}$ at fixed times (graph distance from the root) are marked.

The approach in [17, proof of Theorem 4.8] relies on the so-called *lace expansion* and involves an inductive argument (on *r*). To be more precise [17] uses the lace expansion on a tree network (introduced in [15] for networks of self-avoiding walks) in the context of lattice trees, with the expansion applied at the closest branch point to the root in the network $\mathcal {N}$. The expansion gives rise to certain diagrams that involve lattice trees connecting or intersecting in various ways. Some of these connections are of fixed temporal length, and others are of unrestricted length. A crucial part of the analysis involves bounding these diagrams. The bounds depend on the complexity of the diagram, as well as the total temporal length in the diagram. Diagrams where either the complexity or the length is large give small contributions (recall that we are in high dimensions), as they are asking for either lots of intersections, or for intersections to occur over a large distance.

**Fig. 10:**
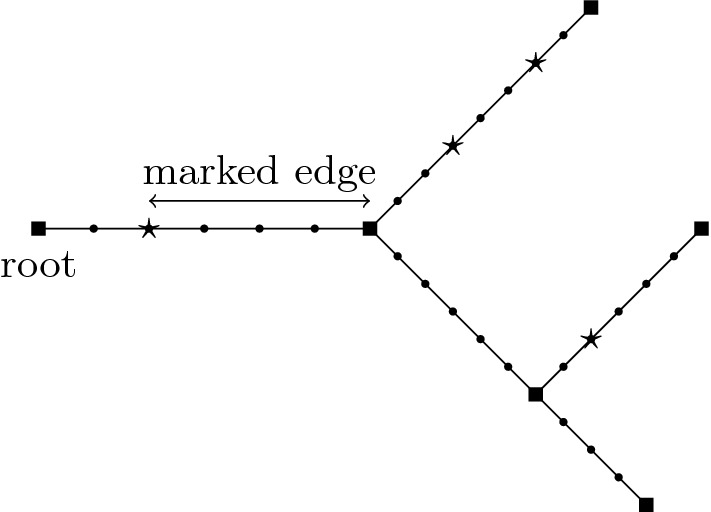
An example of a marked skeleton network from a shape ${\digamma }\in \Sigma _4$. Branch points and leaves are $\blacksquare$, marked points are $\star$

The point of this discussion is that, in our setting, when $j_e$ need not be 1, one can perform exactly the same expansion. It turns out that there are essentially no new diagrams to deal with in our setting. Below we introduce the definition of a marked skeleton network (see also Fig. 10) and then proceed in the following subsections to expand the above outline of the proof of Proposition 2.11.

### Definition 4.1

Given ${\digamma }\in \Sigma _r$ and $\check{\varvec{n}}=(\check{n}_{e,i})_{i\in [j_e],e\in [2r-1]}$ where $j_e\in \mathbb {N}$ for each $e\in \mathcal {E}({\digamma })$, define $\mathcal {N}^+({\digamma },\check{\varvec{n}})$ to be the *marked skeleton network* which is obtained from ${\digamma }$ by

- inserting $j_e-1$ *marked points* into edge *e* of ${\digamma }$ for each $e\in [2r-1]$, thus each edge *e* in ${\digamma }$ becomes a path of $j_e$ edges, called *marked edges*, which are labelled as (*e*, *i*) for $i\le j_e$; and
- inserting $\check{n}_{e,i}-1$ vertices into every marked edge (*e*, *i*), so $\check{n}_{e,i}\in \mathbb {N}$ denotes the length of the marked edge (*e*, *i*).

Write $\varvec{E}(\mathcal {N}^+)$ for the set of marked edges of $\mathcal {N}^+$. Marked edges are *adjacent* if they share a vertex in common.

- The *branch* $\mathcal {N}^+_e$ of $\mathcal {N}^+$ associated to an edge *e* of ${\digamma }$ is the set of vertices of $\mathcal {N}^+$ consisting of the endpoints of *e* together with all points (marked or not) inserted into that edge as per the definition of $\mathcal {N}^+$. The set of branches is written $B(\mathcal {N}^+):=(\mathcal {N}^+_e)_{e \in \mathcal {E}({\digamma })}$. Two distinct branches $\mathcal {N}^+_e$ and $\mathcal {N}^+_{e'}$ are *adjacent* if and only if they have a vertex in common (equivalent to *e* and $e'$ being adjacent in ${\digamma }$).
- A *special point* of $\mathcal {N}^+$ is any marked point, branch point or leaf. $\blacktriangleleft$

### Remark 4.2

The sets of (all) vertices and edges of a marked skeleton network $\mathcal {N}^+$ will be denoted by $\mathcal {N}^+$ and $E(\mathcal {N}^+)$ respectively (note the abuse of notation that $\mathcal {N}^+$ denotes both the marked skeleton and its set of vertices). The cardinality of $E(\mathcal {N}^+)$ is $\#E(\mathcal {N}^+)=\sum _{e \in {\digamma }}\sum _{i=1}^{j_e}\check{n}_{e,i}$ and the number of vertices is 1 larger. All special points are also vertices of $\mathcal {N}^+$, while marked edges should be considered as distinct objects from edges, even for marked edges (*e*, *i*) such that $\check{n}_{e,i}=1$ (note that we have thus far specified a labelling scheme for marked edges, but not edges). The set of marked edges of $\mathcal {N}^+$ is $\varvec{E}(\mathcal {N}^+)$. $\bigstar$

### Asymptotics of the detailed 1-particle transform

For the case where $r=1$, there exists only one shape in $\Sigma _1$ which consists of a single edge *e*. In this case, we use the notation $[\check{n}_1,\ldots , \check{n}_\ell ]$, for $(\check{n}_i)_{i\le \ell }\in \mathbb {N}^{\ell }$ with $\ell \ge 0$ to designate the corresponding marked skeleton network (containing no branch point) with $\check{\varvec{n}} = \{\check{n}_{e,1},\ldots , \check{n}_{e,\ell }\}$.

One of the main results of [18] (see Theorem 4.3(ii) of that reference) can be reformulated as the following proposition (the error terms are not stated explicitly in [18, Theorem 4.3], but if we keep track of them we get the following result), which is the $r=1$ case of Proposition 2.11:

#### Proposition 4.3

Fix $d>8$. There exists $L_0(d)\gg 1$ such that for all $L\ge L_0$:

For each $\delta \in (0, 1\wedge \frac{d-8}{2})$, $R>0$, every $\ell \in \mathbb {N}$ and $(\check{n}_i)_{i\le \ell }\in \mathbb {N}^{\ell }$ and for any $\check{\varvec{k}} \in [-R,R]^{\ell }$ we have, for the unique shape ${\digamma }\in \Sigma _1$,

$$\begin{aligned} \hat{t}^{({\digamma })}_{\check{\varvec{n}}}\Big (\frac{\check{\varvec{k}}}{\sqrt{n}}\Big ) =C_A\prod _{i=1}^{\ell } {\textrm{e}}^{-\sigma _0^2\frac{{\check{k}}_i^2}{2} (\frac{\check{n}_i}{n})}+\mathcal {O}\left( \sum _{i=1}^\ell \frac{1}{\check{n}_i^{\frac{d-8}{2}}}\right) +\mathcal {O}\left( |\check{\varvec{k}}|^2\sum _{i=1}^\ell \frac{\check{n}_i^{1-\delta }}{n}\right) , \end{aligned}$$

where the error depends on *R*, $\delta$, *L*, *d*, $\ell$, and any lower bound on $\min _{i\le \ell } \check{n}_i/n$ and upper bound on $\max _{i\le \ell }\check{n}_i/n$.

Note that in [18] each $\check{n}_i$ is of the form $\lfloor nt_i \rfloor -\lfloor nt_{i-1} \rfloor$, where $0=t_0<t_1<\dots <t_\ell \le t^*$ and where the error term depends on $\min \{t_i-t_{i-1}\}$ and $t^*$.

### Lace expansion

We will use the lace expansion (and induction on *r*) to reduce our required estimates on a shape in $\Sigma _r$ with $r \ge 2$ to the shape in $\Sigma _1$. In the following we let $\mathcal {N}^+=\mathcal {N}^+({\digamma },\check{\varvec{n}})$ for some ${\digamma }\in \Sigma _r$ and some $\check{\varvec{n}}$, where $r\ge 2$. Since each $\check{n}_{e,i}/n\ge \varepsilon$ in Proposition 2.11, for fixed $\varepsilon$ we may assume that *n* is sufficiently large so that each $\check{n}_{e,i}\ge 2$ in what follows.

#### Definition 4.4

If $\mathcal {N}^+$ is a marked skeleton network, we say that $\mathcal {M}^+$ is a *marked subnetwork* of $\mathcal {N}^+$ and write $\mathcal {M}^+\subset \mathcal {N}^+$ if

- as a graph, $\mathcal {M}^+$ is a (connected) subgraph of $\mathcal {N}^+$, and
- the marked points of $\mathcal {M}^+$ are those vertices in $\mathcal {M}^+$ that were marked points in $\mathcal {N}^+$ (i.e. marked points are inherited from $\mathcal {N}^+$).

As usual we also write $\mathcal {M}^+$ for the set of vertices of the marked subnetwork $\mathcal {M}^+$. $\blacktriangleleft$

#### Definition 4.5

Let $\mathcal {M}^+$ be a marked subnetwork of some marked skeleton network $\mathcal {N}^+$.

**Fig. 11:**
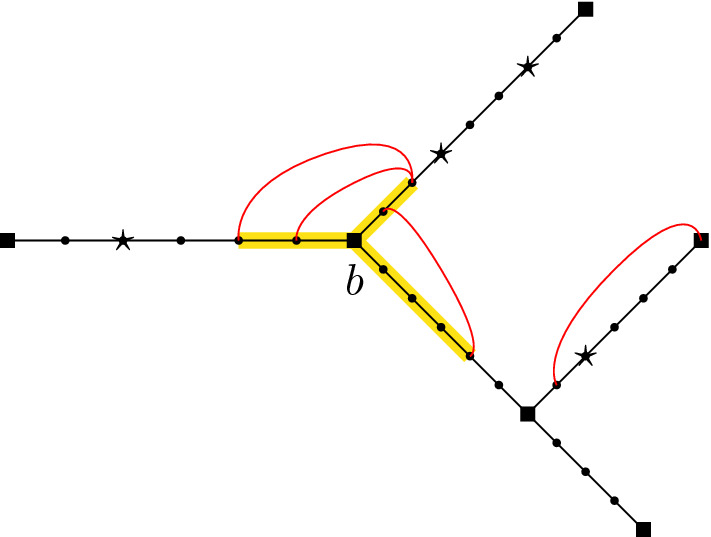
A graph $\Gamma$ on a marked skeleton network $\mathcal {N}^+$, with *b* denoting the branch point nearest to the root. The rightmost bond is in $\mathcal {R}$ since it covers two special points. Also, $\Gamma \in \mathcal {E}^b_{\mathcal {N}^+}$ since $\mathcal {A}_b(\Gamma )$ (highlighted) contains a neighbour of a marked point

1. A *bond* $vv'$ is a pair of distinct vertices $v,v'$ of $\mathcal {M}^+$. The set of vertices in the unique path in $\mathcal {M}^+$ from *v* to $v'$ is written $[v,v']$. We say that the bond $vv'$ covers the vertices in $[v,v']$ (and the edges therein). We write $vv'\in \mathcal {M}^+$ to mean that $vv'$ is a bond in $\mathcal {M}^+$.
2. A *graph* on $\mathcal {M}^+$ is a set of bonds and we denote the set of graphs on $\mathcal {M}^+$ by $\mathcal {G}_{\mathcal {M}^+}$.
3. Let $\mathcal {R}_{\mathcal {M}^+}$ denote the set of bonds in $\mathcal {M}^+$ that cover 2 or more special points. Furthermore set $\mathcal {G}_{\mathcal {M}^+}^{-\mathcal {R}}=\{\Gamma \in \mathcal {G}_{\mathcal {M}^+},\ \Gamma \cap \mathcal {R}_{\mathcal {M}^+}=\emptyset \}$, i.e. the graphs on $\mathcal {M}^+$ that do not contain any bond in $\mathcal {R}_{\mathcal {M}^+}$.
4. A graph $\Gamma \in \mathcal {G}_{\mathcal {M}^+}$ is said to be *connected* on $\mathcal {M}^+$ if every edge of $\mathcal {M}^+$ is covered by some $st\in \Gamma$. Let $\mathcal {G}_{\mathcal {M}^+}^{\text {con}}$ be the set of connected graphs on $\mathcal {M}^+$, and $\mathcal {G}^{-\mathcal {R},\text {con}}_{\mathcal {M}^+}=\mathcal {G}_{\mathcal {M}^+}^{\text {con}}\cap \mathcal {G}_{\mathcal {M}^+}^{-\mathcal {R}}$.
5. Given $\Gamma \in \mathcal {G}_{\mathcal {M}^+}$ and $\mathcal {A} \subset \mathcal {M}^+$, we define $\left. \Gamma \right| _{\mathcal {A}}=\{vv' \in \Gamma ,\ v,v'\in \mathcal {A}\}$.
6. For a vertex $v\in \mathcal {M}^+$ and $\Gamma \in \mathcal {G}_{\mathcal {M}^+}$, we let $\mathcal {A}_v(\Gamma )$ be the largest connected subnetwork $\mathcal {A}$ of $\mathcal {M}^+$ containing *v* and such that $\left. \Gamma \right| _{\mathcal {A}}$ is a connected graph on $\mathcal {A}$. In words, this is the connected component of covered (by $\Gamma$) vertices containing *v*. By convention we take $\mathcal {A}_v(\Gamma )=\{v\}$ if no bond in $\Gamma$ covers *v* .
7. If $v \in \mathcal {N}^+$, we let $\mathcal {E}_{\mathcal {N}^+}^v$ denote the set of graphs $\Gamma \in \mathcal {G}_{\mathcal {N}^+}$ such that $\mathcal {A}_v(\Gamma )$ contains a vertex adjacent to some special point $u\ne v$ of $\mathcal {N}^+$, and $\mathcal {E}_{\mathcal {N}_+}^{-\mathcal {R},v}=\mathcal {G}_{\mathcal {N}^+}^{-\mathcal {R}}\cap \mathcal {E}_{\mathcal {N}^+}^v$. See e.g. Fig. 11. $\blacktriangleleft$

In this section, for a bond $vv'\in \mathcal {N}^+$, $U_{vv'}$ will denote a quantity in $\{-1,0\}$. Observe that (with $\mathcal {R}=\mathcal {R}_{\mathcal {N}^+}$),

4.1 $$\begin{aligned} \prod _{vv'\in \mathcal {N}^+} [1+U_{vv'}]=\prod _{vv'\in \mathcal {N}^+\setminus \mathcal {R}} [1+U_{vv'}]-\Bigl (\prod _{vv'\in \mathcal {N}^+\setminus {\mathcal {R}}} [1+U_{vv'}]\Bigr )\Bigl (1-\prod _{vv'\in \mathcal {R}} [1+U_{vv'}]\Bigr ). \end{aligned}$$

#### Definition 4.6

For $m\in \mathbb {Z}_+^{3}$ we write $\mathcal {S}_{m }$ for the (unmarked) network consisting of paths of lengths $(m_j)_{j=1}^3$ respectively meeting at a common vertex. If exactly *i* of the $m_j$ are strictly positive then this is a *star-shaped network* of degree *i*. The case $i=0$ is a single vertex. The *central point* of $\mathcal {S}_{{m}}$ is the common vertex of the 3 paths. $\blacktriangleleft$

#### Definition 4.7

For a marked skeleton network $\mathcal {N}^+=\mathcal {N}^+({\digamma }, \check{\varvec{n}})$ with ${\digamma }\in \Sigma _r$ for some $r\ge 2$, let *b* denote the branch point lying on the same branch as the root. Let $\mathcal {S}_{\mathcal {N}^+}^-$ be the largest subnetwork of $\mathcal {N}^+$ containing *b* and which does not contain a neighbour of any other special point of $\mathcal {N}^+$ $\blacktriangleleft$

#### Remark 4.8

If $\Gamma \in \mathcal {G}_{\mathcal {N}^+}^{-\mathcal {R}}\setminus \mathcal {E}^b_{\mathcal {N}_+}$ then $\mathcal {A}_b(\Gamma )$ is a (connected subnetwork of a) star-shaped network of degree at most 3 (since ${\digamma }\in \Sigma _r$ with $r\ge 2$). $\bigstar$

#### Definition 4.9

If $\mathcal {N}^+$ is a marked skeleton network and $\mathcal {A}\subset \mathcal {S}_{\mathcal {N}^+}^-$ with $b\in \mathcal {A}$, then the vertex set $\mathcal {N}^+\setminus \mathcal {A}$ (with the edge structure and marked points induced from $\mathcal {N}^+$) consists of exactly three marked skeleton networks (each of which is connected) that we write as $(\mathcal {N}^+\setminus \mathcal {A})_i$ for $i=1,2,3$. Those three subnetworks together contain all special points of $\mathcal {N}^+$ except *b*. $\blacktriangleleft$

For a subnetwork $\mathcal {A}\subset \mathcal {N}^+$, let $\mathcal {K}(\mathcal {A})=\sum _{\Gamma \in \mathcal {G}_{\mathcal {A}}^{-\mathcal {R}}} \prod _{st\in \Gamma } U_{st}$. Then we can write

4.2 $$\begin{aligned} \mathcal {K}(\mathcal {N}^+)&:=\sum _{\Gamma \in \mathcal {G}_{\mathcal {N}^+}^{-\mathcal {R}}} \prod _{st\in \Gamma } U_{st}\nonumber \\&=\sum _{\begin{array}{c} \mathcal {A}\subset \mathcal {S}_{\mathcal {N}^+}^- \\ b\in \mathcal {A} \end{array}} \,\,\sum _{\Gamma \in \mathcal {G}_{\mathcal {A}}^{-\mathcal {R},\text {con}}} \prod _{st\in \Gamma } U_{st} \prod _{i=1}^3 \Bigl [\sum _{\Gamma _i \in \mathcal {G}^{-\mathcal {R}}_{(\mathcal {N}^+\setminus \mathcal {A})_i}} \prod _{v_iv_i'\in \Gamma _i} U_{v_iv'_i}\Bigr ]+\sum _{\Gamma \in \mathcal {E}_{\mathcal {N}^+}^{-\mathcal {R},b}} \prod _{st\in \Gamma } U_{st}, \end{aligned}$$

with the convention that $\sum _{\Gamma _i \in \mathcal {G}^{-\mathcal {R}}_{\emptyset }} \sum _{v_iv_i'\in \Gamma _i} U_{v_iv'_i}=1$. In words this decomposition says that the set of graphs $\Gamma$ on $\mathcal {N}^+$ containing no bonds that cover two or more special points consists of (i) those graphs $\Gamma$ for which the induced connected subnetwork containing *b* also contains a neighbour of some other special point (this is the last term in (4.2)) and (ii) those graphs $\Gamma$ for which this induced subnetwork does not contain the neighbour of another special point. For (ii) the induced connected subnetwork is some set $\mathcal {A}$ contained in $\mathcal {S}_{\mathcal {N}^+}^-$ so we can first sum over the possibilities for $\mathcal {A}$ and then sum over connected graphs on $\mathcal {A}$ and graphs on each $(\mathcal {N}^+\setminus \mathcal {A})_i$. Introducing

$$\begin{aligned} \mathcal {J}(\mathcal {A})=\sum _{\Gamma \in \mathcal {G}_{\mathcal {A}}^{-\mathcal {R},\text {con}}} \prod _{st\in \Gamma } U_{st}, \end{aligned}$$

then (4.2) becomes

4.3 $$\begin{aligned} \mathcal {K}(\mathcal {N}^+)=\sum _{\begin{array}{c} \mathcal {A}\subset \mathcal {S}_{\mathcal {N}^+}^-: \\ b\in \mathcal {A} \end{array}} \mathcal {J}(\mathcal {A}) \prod _{i=1}^3 \mathcal {K}\big ((\mathcal {N}^+\setminus \mathcal {A})_i\big )+\sum _{\Gamma \in \mathcal {E}_{\mathcal {N}^+}^{-\mathcal {R},b}} \prod _{st\in \Gamma } U_{st}. \end{aligned}$$

### Application of the Lace expansion

Given $\mathcal {N}^+=\mathcal {N}^+({\digamma },\check{\varvec{n}})$ for some ${\digamma }\in \Sigma _r$ ($r\ge 1$), and $\check{\varvec{n}}=(\check{n}_{e,i} )_{i \in [j_e],e\in [2r-1]}\in \mathbb {N}^{\varvec{E}(\mathcal {N}^+)}$, and given $\check{\varvec{y}}=(\check{y}_{e,i})_{i \in [j_e],e\in [2r-1]}\in (\mathbb {Z}^d)^{\varvec{E}(\mathcal {N}^+)}$, define $t_{\mathcal {N}^+}(\check{\varvec{y}})=t^{({\digamma })}_{\check{\varvec{n}}}(\check{\varvec{y}})$. This notation will help us deal with various subnetworks. Recalling (2.22), we have

$$\begin{aligned} t_{\mathcal {N}^+}(\check{\varvec{y}}) = \sum _{T\in \check{\varvec{T}}(\check{\varvec{n}},\check{\varvec{y}})} W(T). \end{aligned}$$

#### Definition 4.10

Given $\mathcal {N}^+$ and $\check{\varvec{y}}$ as above, we define $\Omega _{\mathcal {N}^+}(\check{\varvec{y}})$ to be the set of embeddings $\omega =(\omega (s))_{s\in \mathcal {N}^+}$ of $\mathcal {N}^+$ into $\mathbb {Z}^d$ such that

1. the root is mapped to 0,
2. adjacent vertices in $\mathcal {N}^+$ are mapped to points in $\mathbb {Z}^d$ at ($\ell _\infty$) distance at most *L* from each other.
3. the endpoint of the marked edge (*e*, *j*) that is farthest from the root (this endpoint is necessarily a special point) is mapped to $\sum _{f\prec e}\sum _{k\le j_f} \check{y}_{f,k} + \sum _{k\le j} \check{y}_{e,k}$ for all $e\in [2r-1]$ and $j\le j_e$.$\blacktriangleleft$

For a collection of lattice trees $(R_s)_{s\in \mathcal {N}^+}$ and for a bond (pair of distinct vertices) *st* of $\mathcal {N}^+$ define

4.4 $$\begin{aligned} U_{st}={\left\{ \begin{array}{ll} 0, &{} \text { if } R_s\cap R_t=\emptyset , \\ -1, &{} \text { otherwise.} \end{array}\right. } \end{aligned}$$

#### Definition 4.11

Given $x\in \mathbb {Z}^d$, we write $\sum _{R \ni x}$ to denote a sum over lattice trees *R* containing the point $x\in \mathbb {Z}^d$. $\blacktriangleleft$

As for [17, Eq. (4.17)] we can write

4.5 $$\begin{aligned} t_{\mathcal {N}^+}(\check{\varvec{y}})=\sum _{\omega \in \Omega _{\mathcal {N}^+}(\check{\varvec{y}})} W(\omega ) \sum _{\begin{array}{c} (R_s)_{s \in \mathcal {N}^+}:\\ R_s\ni \omega (s) \forall s \in \mathcal {N}^+ \end{array}} \prod _{t \in \mathcal {N}^+}W(R_t) \prod _{uu'\in \mathcal {N}^+} [1+U_{uu'}], \end{aligned}$$

as any combination $(\omega \in \Omega _{\mathcal {N}^+}(\check{\varvec{y}}), (R_s)_{s\in \mathcal {N}^+})$ such that the $R_s$ are mutually avoiding lattice trees, uniquely defines a lattice tree $T\in \check{\varvec{T}}({\digamma }, \check{\varvec{n}},\check{\varvec{y}})$ and vice versa. Here, $R_s$ is the tree hanging off the vertex $s\in \mathcal {N}^+$. Note that in the shorthand notation of [17] (4.5) would be written as

4.5' $$\begin{aligned} t_{\mathcal {N}^+}(\check{\varvec{y}})=\sum _{\omega \in \Omega _{\mathcal {N}^+}(\check{\varvec{y}})} W(\omega ) \prod _{s\in \mathcal {N^+}} \sum _{R_s\ni \omega (s) } W(R_s) \prod _{uu'\in \mathcal {N}^+} [1+U_{uu'}]. \end{aligned}$$

Recalling Definition 4.5 and (4.1), we set

4.6 $$\begin{aligned} \phi _{\mathcal {N}^+}^{\mathcal {R}}(\check{\varvec{y}})=\sum _{\omega \in \Omega _{\mathcal {N}^+}(\check{\varvec{y}})} W(\omega ) \sum _{\begin{array}{c} (R_s)_{s \in \mathcal {N}^+}:\\ R_s\ni \omega (s) \forall s \in \mathcal {N}^+ \end{array}} \prod _{t \in \mathcal {N}^+}W(R_t) \Bigl (\prod _{uu'\in \mathcal {R}^c} [1+U_{uu'}]\Bigr ) \Bigl (1-\prod _{vv'\in \mathcal {R}} [1+U_{vv'}]\Bigr ), \end{aligned}$$

which is 0 unless $U_{vv'}=-1$ for some $vv'\in \mathcal {R}$, and (recalling the last term in (4.3))

$$\begin{aligned} \phi _{\mathcal {N}^+}^{b}(\check{\varvec{y}})=\sum _{\omega \in \Omega _{\mathcal {N}^+}(\check{\varvec{y}})} W(\omega ) \sum _{\begin{array}{c} (R_s)_{s \in \mathcal {N}^+}:\\ R_s\ni \omega (s) \forall s \in \mathcal {N}^+ \end{array}} \prod _{t \in \mathcal {N}^+}W(R_t)\sum _{\Gamma \in \mathcal {E}_{\mathcal {N}^+}^{-\mathcal {R},b}} \prod _{vv'\in \Gamma } U_{vv'}. \end{aligned}$$

By (4.1) we have

$$\begin{aligned} t_{\mathcal {N}^+}(\check{\varvec{y}})=\sum _{\omega \in \Omega _{\mathcal {N}^+}(\check{\varvec{y}})} W(\omega ) \sum _{\begin{array}{c} (R_s)_{s \in \mathcal {N}^+}:\\ R_s\ni \omega (s) \forall s \in \mathcal {N}^+ \end{array}} \prod _{t \in \mathcal {N}^+}W(R_t) \mathcal {K}(\mathcal {N}^+)-\phi _{\mathcal {N}^+}^{\mathcal {R}}(\check{\varvec{y}}) \end{aligned}$$

and by (4.3)

4.7 $$\begin{aligned}&t_{\mathcal {N}^+}(\check{\varvec{y}})\nonumber \\&\quad =\sum _{\omega \in \Omega _{\mathcal {N}^+}(\check{\varvec{y}})} W(\omega ) \sum _{\begin{array}{c} (R_s)_{s \in \mathcal {N}^+}:\\ R_s\ni \omega (s) \forall s \in \mathcal {N}^+ \end{array}} \prod _{t \in \mathcal {N}^+}W(R_t) \sum _{\begin{array}{c} \mathcal {A}\subset \mathcal {S}_{\mathcal {N}^+}^-, \\ b\in \mathcal {A} \end{array}} \mathcal {J}(\mathcal {A}) \prod _{i=1}^3 \mathcal {K}((\mathcal {N}^+\setminus \mathcal {A})_i)\nonumber \\&\quad \qquad +\phi _{\mathcal {N}^+}^{b}(\check{\varvec{y}})-\phi _{\mathcal {N}^+}^{\mathcal {R}}(\check{\varvec{y}}). \end{aligned}$$

This decomposition is related to Fig. 11 where, loosely speaking, the term in $\mathcal {J}$ corresponds the interactions induced by bonds around the first branch point and the three terms in $\mathcal {K}$ correspond to three new smaller networks. Some notation associated to this decomposition is introduced in the next definition.

#### Definition 4.12

For a marked skeleton network $\mathcal {N}^+$, let $\check{e}_1,\check{e}_2,\check{e}_3$ be the three marked edges incident to the branch point *b*. Note that each for $k=1,2,3$, $\check{e}_k=(e_k,i_k)$ for some $e_k\in [2r-1]$ and some $i_k\in \{1,j_{e_k}\}$ (this marked edge is necessarily the last marked edge on the branch containing the origin and the first marked edge on the other two branches containing *b*).

Given ${m}=(m_k)_{k=1}^3$ such that $0\le m_k\le \check{n}_{\check{e}_k}-2$ where $\check{n}_{\check{e}_k}:= \check{n}_{e_k,i_k},k=1,2,3$, we define $(\mathcal {N}^+_{k,{m}})_{k=1,2,3}$ as the three components of $\mathcal {N}^+ \setminus \mathcal {S}_{{m}}$ as in Definition 4.9 (recall Definition 4.6, and note that each $\mathcal {N}^+_{k,{m}}$ is itself a marked skeleton network). Since each $m_k< \check{n}_{e_k,i_k}$, there is a bijection between marked edges of $\mathcal {N}^+$ and the marked edges of $(\mathcal {N}^+_{k,{m}})_{k=1,2,3}$. The marked edge $\check{e}_k$ is split between $\mathcal {S}_{{m}}$ and $\mathcal {N}^+_{k,{m}}$, but we will abuse notation by retaining this label to refer to the corresponding truncated edge in both components.

Set $\varvec{E}^*(\mathcal {N}^+_{k,{m}})=\varvec{E}(\mathcal {N}^+_{k,{m}})\setminus \{\check{e}_k\}$. For $k=1,2,3$, write $\check{\varvec{n}}^{{m}, k} \in \mathbb {N}^{\varvec{E}(\mathcal {N}^+_{k,{m}})}$ for the vector whose components encode the lengths of marked edges in $\mathcal {N}^+_{k,{m}}$, i.e.

$$\begin{aligned} \check{n}^{{m}, k}_{\check{e}}= {\left\{ \begin{array}{ll} \check{n}_{\check{e}} &{}\text { if } \check{e}\in \varvec{E}^*(\mathcal {N}^+_{k,{m}}),\\ \check{n}_{\check{e}}-(m_k+1) &{}\text { if } \check{e}=\check{e}_k. \end{array}\right. } \end{aligned}$$

Similarly for $\check{\varvec{y}}\in (\mathbb {Z}^d)^{\varvec{E}(\mathcal {N}^+)}$, $k \in \{1,2,3\}$ and $v_k\in \mathbb {Z}^d$ we write $\check{\varvec{y}}^{v_k, k}\in (\mathbb {Z}^d)^{\varvec{E}(\mathcal {N}^+_{k,{m}})}$ for the vector whose components are

$$\begin{aligned} \check{y}^{v_k, k}_{\check{e}}= {\left\{ \begin{array}{ll} \check{y}_{\check{e}} &{}\text { if } \check{e}\in \varvec{E}^*(\mathcal {N}^+_{k,{m}}),\\ \check{y}_{\check{e}}-v_k &{}\text { if } \check{e}=\check{e}_k. \end{array}\right. } \end{aligned}$$

$$\blacktriangleleft $$

Let ${\check{\varvec{n}}}^{ b}:=(\check{n}_{\check{e}_1},\check{n}_{\check{e}_2},\check{n}_{\check{e}_3})$ be the lengths of the marked edges adjacent to *b* in $\mathcal {N}^+$. Define

$$\begin{aligned} \mathcal {H}_{{\check{\varvec{n}}}^{ b}}= & {} \big \{{m} \ :\ 0\le m_k\le \frac{\check{n}_{\check{e}_k}}{3}\wedge (\check{n}_{\check{e}_k}-2), \ k=1,2,3 \big \}\\ \overline{\mathcal {H}}_{{\check{\varvec{n}}}^{ b}}= & {} \{{m} \ :\ 0\le m_k\le \check{n}_{\check{e}_k}-2, \ k=1,2,3 \} \setminus \mathcal {H}_{{\check{\varvec{n}}}^{ b}}. \end{aligned}$$

#### Remark 4.13

For ${m} \in \mathcal {H}_{{\check{\varvec{n}}}^{ b}}$, we know that for $k\in \{1,2,3\}$ and $\check{e}\in \varvec{E}(\mathcal {N}^+_{k,{m}})$ we have $\frac{2}{3} {\check{n}}_{\check{e}}\le {\check{n}}^{{m}, k}_{\check{e}}\le {\check{n}}_{\check{e}}$ (with ${\check{n}}^{{m}, k}_{\check{e}}= {\check{n}}$ whenever $\check{e}\in \varvec{E}^*(\mathcal {N}^+_{k,{m}})$). In particular, recalling that there is a bijection between marked edges of $\mathcal {N}^+$ and the marked edges of $(\mathcal {N}^+_{k,{m}})_{k=1,2,3}$, we can see that for any $a\in \mathbb {R}$ there exist $c(a), C(a)>0$ such that for ${m} \in \mathcal {H}_{{\check{\varvec{n}}}^{ b}}$

$$\begin{aligned} c(a)\sum _{k=1}^3 \sum _{\check{e}\in \varvec{E}(\mathcal {N}_{k,{m}}^+)} ({\check{n}}^{{m}, k}_{\check{e}})^{a} \le \sum _{\check{e}\in \varvec{E}(\mathcal {N}^+)} \check{n}^{a}_{\check{e}} \le C(a)\sum _{k=1}^3 \sum _{\check{e}\in \varvec{E}(\mathcal {N}_{k,{m}}^+)} ({\check{n}}^{{m}, k}_{\check{e}})^{a}. \end{aligned}$$

$$\bigstar $$

Finally, we set

$$\begin{aligned} \phi _{\mathcal {N}^+}^{\pi }(\check{\varvec{y}})=\sum _{\omega \in \Omega _{\mathcal {N}^+}(\check{\varvec{y}})} W(\omega ) \sum _{\begin{array}{c} (R_s)_{s \in \mathcal {N}^+}:\\ R_s\ni \omega (s) \forall s \in \mathcal {N}^+ \end{array}} \prod _{t \in \mathcal {N}^+}W(R_t) \sum _{{m} \in \overline{\mathcal {H}}_{{\check{\varvec{n}}}^{ b}}} \mathcal {J}(\mathcal {S}_{{m}})\prod _{i=1}^3 \mathcal {K}\bigl (\mathcal {N}^+_{i,{m}} \bigr ), \end{aligned}$$

and (noting the change to the sum over ${m}$)

4.8 $$\begin{aligned} Q_{\mathcal {N}^+}(\check{\varvec{y}})=\sum _{\omega \in \Omega _{\mathcal {N}^+}(\check{\varvec{y}})} W(\omega ) \sum _{\begin{array}{c} (R_s)_{s \in \mathcal {N}^+}:\\ R_s\ni \omega (s) \forall s \in \mathcal {N}^+ \end{array}} \prod _{t \in \mathcal {N}^+}W(R_t) \sum _{{m} \in \mathcal {H}_{{\check{\varvec{n}}}^{ b}}} \mathcal {J}(\mathcal {S}_{{m}})\prod _{i=1}^3 \mathcal {K}\bigl (\mathcal {N}^+_{i,{m}} \bigr ). \end{aligned}$$

From the argument above and (4.7), we can see that

4.9 $$\begin{aligned} t_{\mathcal {N}^+}(\check{\varvec{y}})=Q_{\mathcal {N}^+}(\check{\varvec{y}})+\phi _{\mathcal {N}^+}^{\pi }(\check{\varvec{y}})+\phi _{\mathcal {N}^+}^{b}(\check{\varvec{y}})-\phi _{\mathcal {N}^+}^{\mathcal {R}}(\check{\varvec{y}}). \end{aligned}$$

The last three terms are error terms. The relevant estimates (bounds) are given in the following lemma, whose proof (which is very similar to the corresponding error bounds in [17]) will be presented in Sect. 4.6.

#### Lemma 4.14

Fix $d>8$. There exists $L_0(d)$ such that for all $L\ge L_0$ and for a marked skeleton network $\mathcal {N}^+$, 
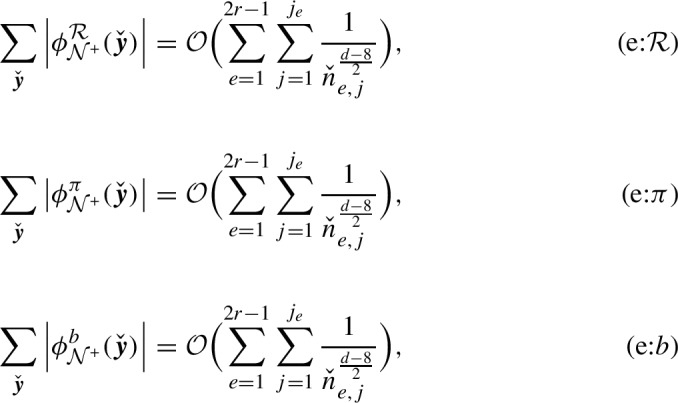
 where the constants in the $\mathcal {O}$ notation depend on *d* and the number of special points in $\mathcal {N}^+$.

We end this section by introducing an important quantity that will appear in the decomposition of $Q_{\mathcal {N}^+}$ and describes the interactions induced by the term $\mathcal {J}(\mathcal {S}_{{m}})$ in (4.8).

#### Definition 4.15

For ${m}\in \mathbb {Z}_+^3$ and ${u} \in (\mathbb {Z}^d)^{3}$ we define $\pi _{{0}}({u})=\rho \mathbb {1}_{\{{u}=0\}}$ and if some $m_i>0$,

$$\begin{aligned} \pi _{{m}}({u})= \sum _{\omega \in \Omega _{\mathcal {S}_{{m}}({u})} }W(\omega ) \sum _{\begin{array}{c} (R_s)_{s \in \mathcal {S}_{{m}}}:\\ R_s\ni \omega (s) \forall s \in \mathcal {S}_{{m}} \end{array}} \prod _{t \in \mathcal {S}_{{m}}}W(R_t)\mathcal {J}(\mathcal {S}_{{m}}), \end{aligned}$$

where the set of embeddings $\Omega _{\mathcal {S}_{{m}}({u})}$ is defined similarly to Definition 4.10: the root of $\mathcal {S}_{{m}}$ (which is the vertex along branch 1 at graph distance $m_1$ from the central vertex - if $m_1=0$ this is simply the central vertex itself) is mapped to 0; adjacent vertices in $\mathcal {S}_{{m}}$ are mapped to points in $\mathbb {Z}^d$ at distance at most *L*; and the central point is mapped to $u_1$ and the leaves on branches *i* for $i=2,3$ are mapped to $u_1+u_2$ and $u_1+u_3$ respectively. $\blacktriangleleft$

#### Remark 4.16

This definition of $\pi _{{m}}$ is exactly the same as the one in [17] (Definition 4.12) and as such the results on this quantity, that rely heavily on diagrammatic estimates, can be transferred directly to our context. $\bigstar$

Recall from (2.21) and the discussion thereafter that $C_V=\rho ^{2}V$. The constant *V* was defined in [17, (4.30)] as

4.10 $$\begin{aligned} V=z_D^3\sum _{{m}\in \mathbb {Z}_+^3}\sum _{{u}\in (\mathbb {Z}^d)^3}\pi _{{m}}({u})=z_D^3\sum _{{m}\in \mathbb {Z}_+^3}\hat{\pi }_{{m}}(0). \end{aligned}$$

For $N\in \mathbb {N}$ we define

4.11 $$\begin{aligned} \pi ^{\scriptscriptstyle (N)}_{{m}}({u})=\sum _{L\in \mathcal {L}^{\scriptscriptstyle (N)}(\mathcal {S}_{{m}})} \sum _{\omega \in \Omega _{\mathcal {S}_{{m}}({u})}} W(\omega )\sum _{\begin{array}{c} (R_s)_{s \in \mathcal {S}_{{m}}}:\\ R_s\ni \omega (s) \forall s \in \mathcal {S}_{{m}} \end{array}}\ \prod _{t \in \mathcal {S}_{{m}}}W(R_t) \prod _{st\in L}(-U_{st})\prod _{vv'\in \mathcal {C}(L)}(1+U_{vv'}), \end{aligned}$$

where $L\in \mathcal {L}^{\scriptscriptstyle (N)}(\mathcal {S}_{{m}})$ is the set of *laces on* $\mathcal {S}_{{m}}$ *with* *N* *bonds* and $\mathcal {C}(L)$ denotes the set of bonds which are *compatible with* *L*. We refer to [17, Section 2] for the precise definitions, and give only a rough description here: A lace *L* on $\mathcal {S}_{{m}}$ is either a minimal graph covering $\mathcal {S}_{{m}}$ (i.e. the removal of any bond in *L* results in a graph that no longer covers $\mathcal {S}_{{m}}$) or one that is almost minimal (in this case there is a bond covering the branch point whose removal results in a minimal graph covering $\mathcal {S}_{{m}}$). There is a rule for (uniquely) defining a lace $\varvec{L}(\Gamma )$ associated to a connected graph $\Gamma$ on $\mathcal {S}_{{m}}$. For a fixed lace *L* the bonds compatible with *L* are those for which adding them to *L* results in a connected graph $\Gamma '$ for which $\varvec{L}(\Gamma ')=L$.

In our work we only need a few facts about $\pi _{{m}}^{\scriptscriptstyle (N)}(\cdot )$, including the obvious fact that $\pi _{{m}}^{\scriptscriptstyle (N)}({u})\ge 0$ and that (see [17, (4.28)–(4.29)]) if some $m_i>0$ then

4.12 $$\begin{aligned} \pi _{{m}}({u})=\sum _{N=1}^\infty (-1)^N\pi _{{m}}^{\scriptscriptstyle (N)}({u}). \end{aligned}$$

Equations (4.12) and (4.11) are the lace expansion. A key result about this expansion is the following minor correction of [17, Proposition 4.13].

#### Proposition 4.17

Fix $d>8$. There exists $L_0(d)$ such that for all $L\ge L_0$ there exists a constant $C>0$ (independent of *L*) and $B_N({m})>0$ such that for all $N\ge 1$ and ${\ell }=(\ell _1,\ell _2,\ell _3)\in \mathbb {Z}_+^3\setminus \{(0,0,0)\}$ we have for $j \in \{1,2,3\}$,

4.13 $$\begin{aligned}&\sum _{{u} \in (\mathbb {Z}^d)^3} |u_j|^{2q}\pi _{{m}}^{\scriptscriptstyle (N)}({u}) \le C(L^2N^2 \Vert {m}\Vert _\infty )^q B_N({m}), \qquad \text { for }q\in \{0,1\},\quad \text { and} \end{aligned}$$

4.14 $$\begin{aligned}&\sum _{N=1}^{\infty } \sum _{{m}: m_j \ge \ell _j} B_N({m}) \le \frac{C }{(\ell _j \vee 1)^{\frac{d-8}{2}}}, \quad \text { and} \end{aligned}$$

4.15 $$\begin{aligned}&\sum _{N=1}^{\infty } N^2 \sum _{{m}\le \ell } \Vert {m}\Vert _{\infty } B_N({m}) \le C\times {\left\{ \begin{array}{ll} \Vert \ell \Vert _{\infty }^{\frac{10-d}{2}\vee 0} &{} \text { if } d\ne 10 \\ \log (\Vert {\ell }\Vert _{\infty }\vee 2) &{} \text { if } d=10. \end{array}\right. } \end{aligned}$$

The correction is that the $\vee 1$ and $\vee 2$ are missing in [17, Proposition 4.13], but what we have stated above is what is actually proved therein. Here we have also not included the extra decay in *L* appearing in these bounds in [17, Proposition 4.13] as we do not need it.

### Decomposition of $\mathcal {Q}_{\mathcal {N}^+}$

By (4.8) we can see that $Q_{\mathcal {N}^+({\digamma }, \check{\varvec{n}})}( \check{\varvec{y}})$ can be decomposed into 4 parts: the connected component $\mathcal {S}_{{m}}$ of bonds stemming from the branching point (term in $\mathcal {J}$) and the three subgraphs of $\mathcal {N}^+$ remaining after the removal of this connected component (terms in $\mathcal {K}$). These four subgraphs are not connected by any bonds by definition of $\mathcal {J}$ and $\mathcal {K}$ on the respective subgraphs. Furthermore the star-shaped subgraph $\mathcal {S}_{{m}}$ contains the special point *b*, while all other special points are contained in one of the other subgraphs. This means that our problem can be reduced to three independent similar problems for smaller lengths. This reasoning translates into the following lemma which can be proved exactly as for [17, Lemma 4.14] so we do not repeat the proof. Recall the definition of $\check{\varvec{y}}^{v_i, i}$ and the marked skeleton networks $\mathcal {N}_{i,{m}}^+$ in Definition 4.12.

#### Lemma 4.18

For a marked skeleton network $\mathcal {N}^+=\mathcal {N}^+({\digamma }, \check{\varvec{n}})$ and $\check{\varvec{y}} \in (\mathbb {Z}^d)^{\varvec{E}(\mathcal {N}^+)}$,

$$\begin{aligned} Q_{\mathcal {N}^+}(\check{\varvec{y}})=\sum _{{m} \in \mathcal {H}_{\check{\varvec{n}}_b}} \sum _{{u}\in (\mathbb {Z}^d)^3}\pi _{{m}}({u}) \prod _{i=1}^3 \Big (z_D \sum _{v_i} D(v_i- u_i)t_{\mathcal {N}_{i,{m}}^+}(\check{\varvec{y}}^{v_i, i})\Big ). \end{aligned}$$

For any marked skeleton network $\mathcal {N}^+({\digamma }, \check{\varvec{n}})$, we introduce the Fourier transform of $t_{\mathcal {N}^+}$ and $Q_{\mathcal {N}^+}$ for any $\check{\varvec{k}}=(\check{k}_{e,j})_{j \in [j_e],e\in [2r-1]}$ by

$$\begin{aligned} \hat{t}_{\mathcal {N}^+}(\check{\varvec{k}})=\sum _{\check{\varvec{y}} \in (\mathbb {Z}^d)^{\varvec{E}(\mathcal {N}^+)}} \prod _{e=1}^{2r-1}\prod _{j=1}^{j_e}{\textrm{e}}^{{\textrm{i}}{\check{k}}_{e,j}\check{y}_{e,j}} t_{\mathcal {N}^+}(\check{\varvec{y}}), \end{aligned}$$

$$\begin{aligned} \hat{Q}_{\mathcal {N}^+}(\check{\varvec{k}})=\sum _{\check{\varvec{y}} \in (\mathbb {Z}^d)^{\varvec{E}(\mathcal {N}^+)}} \prod _{e=1}^{2r-1}\prod _{j=1}^{j_e}{\textrm{e}}^{{\textrm{i}}{\check{k}}_{e,j}\check{y}_{e,j}} Q_{\mathcal {N}^+}(\check{\varvec{y}}). \end{aligned}$$

Lemma 4.18 implies that

4.16 $$\begin{aligned} \hat{Q}_{\mathcal {N}^+}(\check{\varvec{k}})=z_D^3\sum _{{m} \in \mathcal {H}_{\check{\varvec{n}}_b}} \hat{\pi }_{{m}}(\check{\varvec{k}}^b) \prod _{i=1}^3 \hat{D}({\check{k}}_{\check{e}_i})\hat{t}_{\mathcal {N}_{i,{m}}^+}(\check{\varvec{k}}^i), \end{aligned}$$

where $\check{\varvec{k}}^b=(\check{k}_{\check{e}_1},\check{k}_{\check{e}_2},\check{k}_{\check{e}_3})$ (meaning the part of $\check{\varvec{k}}$ corresponding to marked edges incident to the branch point *b*) and $\check{\varvec{k}}^i$ denotes the vector of ${\check{k}}_{e,j}$ corresponding to marked edges *e* in $\mathcal {N}^+_{i,{m}}$. Note that (4.16) is exactly the “marked” network analog of the unmarked relation [17, (4.39)].

### Proof of Proposition 2.11

The proof now closely follows that of [17, Theorem 4.8] with obvious (and straightforward) modifications. We will present the main ideas, but not the details. The goal is to prove that

$$\begin{aligned} \hat{t}_{\mathcal {N}^+({\digamma },\check{n})}\Bigl (\frac{\check{\varvec{k}}}{\sqrt{ n}}\Bigr )&=\rho C_V^{r-1}C_A^{2r-1} \prod _{e=1}^{2r-1} \prod _{i=1}^{j_e}{\textrm{e}}^{-\sigma _0^2\frac{\check{k}_{e,i}^2}{2}\frac{\check{n}_{e,i}}{n}}\\&\quad + \mathcal {O}\Bigl (\sum _{e=1}^{2r-1}\sum _{i=1}^{j_e} \frac{1}{\check{n}_{e,i}^{\frac{d-8}{2}}}\Bigr ) +\mathcal {O}\Bigl (\sum _{e=1}^{2r-1}\sum _{i=1}^{j_e}\frac{\left| \check{\varvec{k}}\right| ^2\check{n}_{e,i}^{1-\delta }}{n}\Bigr ). \end{aligned}$$

From (4.9), our bounds on the error terms therein (Lemma 4.14), and (4.16) we have that

4.17 $$\begin{aligned} \hat{t}_{\mathcal {N}^+({\digamma },\check{n})}\Bigl (\frac{\check{\varvec{k}}}{\sqrt{ n}}\Bigr )&=\hat{Q}_{\mathcal {N}^+}\Bigl (\frac{\check{\varvec{k}}}{\sqrt{ n}}\Bigr )+\mathcal {O}\Bigl (\sum _{e=1}^{2r-1}\sum _{i=1}^{j_e} \frac{1}{\check{n}_{e,i}^{\frac{d-8}{2}}}\Bigr )\nonumber \\&=z_D^3\sum _{{m} \in \mathcal {H}_{\check{\varvec{n}}_b}} \hat{\pi }_{{m}}\Big (\frac{\check{\varvec{k}}^b}{\sqrt{n}}\Big ) \prod _{j=1}^3 \hat{D}\Big (\frac{{\check{k}}_{\check{e}_j}}{\sqrt{n}}\Big )\hat{t}_{\mathcal {N}_{j,{m}}^+}\Big (\frac{\check{\varvec{k}}^j}{\sqrt{n}}\Big )+\mathcal {O}\Bigl (\sum _{e=1}^{2r-1}\sum _{i=1}^{j_e} \frac{1}{\check{n}_{e,i}^{\frac{d-8}{2}}}\Bigr ). \end{aligned}$$

We proceed by induction on *r* for networks with shape ${\digamma }\in \Sigma _r$, using Lemma 4.3 for the initializing case ($r=1$).

Let $\delta \in (0,1\wedge \frac{d-8}{2})$. By the induction hypothesis applied to each $\mathcal {N}^+_{j,{m}}$ (having $r_j+1$ leaves, where $r_1=1$ and $r_2+r_3=r$) we may write

4.18 $$\begin{aligned} \prod _{j=1}^3\hat{t}_{\mathcal {N}_{j,{m}}^+}\Big (\frac{\check{\varvec{k}}^j}{\sqrt{n}}\Big )\approx \rho ^3 C_V^{r-2}C_A^{2r-1}\prod _{j=1}^3\Bigg [\prod _{\check{e}\in \varvec{E}(\mathcal {N}_{j,{m}}^+)}{\textrm{e}}^{-\sigma _0^2 \frac{\check{k}^2_{\check{e}}}{2} \frac{\check{n}^{{m},j}_{\check{e}}}{n}}\Bigg ] ,\end{aligned}$$

where we recall that the notation $\check{n}_{\cdot }^{{m},j}$ was introduced in Definition 4.12. The error terms in the above approximation are obtained from the induction hypothesis and Remark 4.13 (using the fact that $\check{n}^{{m},j}_{\check{e}}$ is comparable to $\check{n}_{\check{e}}$—they are identical unless $\check{e} =\check{e}_j$ for some $j\le 3$ - since ${m} \in \mathcal {H}_{\check{\varvec{n}}_b}$). We then use the fact that

$$\begin{aligned}\prod _{j=1}^3 {\textrm{e}}^{-\sigma _0^2 \frac{\check{k}^2_{\check{e}_j}}{2} \frac{\check{n}^{{m},j}_{\check{e}_j}}{n}}-\prod _{j=1}^3 {\textrm{e}}^{-\sigma _0^2 \frac{\check{k}^2_{\check{e}_j}}{2} \frac{\check{n}_{\check{e}_j}}{n}}\le \frac{C}{n}\sum _{j=1}^3 \check{k}_{\check{e}_j}^2m_j ,\end{aligned}$$

(4.13) with $q=0$ and (4.15) of Proposition 4.17, and $|\hat{D}|\le 1$ to get an error term in (4.17) (when replacing the right-hand side of (4.18) with $\check{n}_{\check{e}}$ in the exponent instead of $\check{n}^{{m},j}_{\check{e}}$) of at most $\mathcal {O}(\sum _{j=1}^3\check{k}^2_{\check{e}_j}\check{n}_{\check{e}_j}^{1-\delta }n^{-1})$. For the relevant details of this part of the argument, and in particular for the bounds on the error terms, one can look at the derivation of [17, (4.56)].

Now $$\hat{D}(\check{k}_{\check{e}_j}/\sqrt{n})=1+\mathcal {O}(|\check{k}_{\check{e}_j}|^2/n)$$ and

$$\begin{aligned} |\hat{\pi }_{{m}}(\check{\varvec{k}}^b/\sqrt{n})- \hat{\pi }_{{m}}(0)|\le C\frac{|\check{\varvec{k}}^b|^2}{n}\sum _{j=1}^3\sum _{{u}}|u_j|^2|\pi _{{m}}({u})|,\end{aligned}$$

which, when summed over $m_j\le \check{n}_{\check{e}_j},\ j=1,2,3$, gives at most $CL^2n^{-1}|\check{\varvec{k}}^b|^2\sum _{j=1}^3 \check{n}_{\check{e}_j}^{1-\delta }$ (see (4.13) with $q=1$ and (4.15)). Next, $\sum _{{m}\in \mathcal {H}_{\check{\varvec{n}}_b}}\hat{\pi }_{{m}}(0)$ differs from the full sum $\sum _{{m}}\hat{\pi }_{{m}}(0)$ by at most $C\sum _{j=1}^3 \check{n}_{\check{e}_j}^{-(d-8)/2}$ by (4.14). Combining the above and recalling (4.10) and that $C_V=\rho ^2V$ reveals that

$$\begin{aligned} \hat{t}_{\mathcal {N}^+({\digamma },\check{n})}\Bigl (\frac{\check{\varvec{k}}}{\sqrt{ n}}\Bigr )&\approx z_D^3\sum _{{m}}\hat{\pi }_{{m}}(0)\rho ^3 C_V^{r-2}C_A^{2r-1}\prod _{e=1}^{2r-1}\prod _{i=1}^{j_e}{\textrm{e}}^{-\sigma _0^2 \frac{\check{k}^2_{e,i}}{2} \frac{\check{n}_{e,i}}{n}}\\&=\rho C_V^{r-1}C_A^{2r-1}\prod _{e=1}^{2r-1}\prod _{i=1}^{j_e}{\textrm{e}}^{-\sigma _0^2 \frac{\check{k}^2_{e,i}}{2} \frac{\check{n}_{e,i}}{n}}. \end{aligned}$$

An analysis of the error terms involved in the various $\approx$ approximations is handled rigorously in [17, Sections 4.3–4.5], making use of Proposition 4.17. $\square$

### Proof of Lemma 4.14

The proof of Lemma 4.14 relies on diagrammatic estimates. These estimates are built from a single lemma which gives the bounds on the simplest diagrams. For $u\in \mathbb {Z}^d$ let us denote

$$\begin{aligned} h_m(u)={\left\{ \begin{array}{ll} z_D^2 (D*t_{m-2} *D)(u) &{} \text { if}\, m\ge 2\\ z_D D(u) &{} \text { if}\, m=1\\ \mathbb {1}_{\{u=0\}} &{} \text { if}\, m=0, \end{array}\right. } \end{aligned}$$

where $t_m(u)=\rho \mathbb {P}(u \in \mathcal {T}_m)$ (so $t_0(u)=\rho \mathbb {1}_{\{u=0\}}$), and we recall that $*$ denotes the convolution of functions on $\mathbb {Z}^d$. Note that in [17] there is a $\zeta$ in the definition, but this $\zeta =1$ because of Lemma 3.9 of [17]. Note that for $m\ge 2$,

4.19 $$\begin{aligned} t_m(u)&=\sum _{\omega :0\overset{m}{\rightarrow }u}W(\omega )\sum _{\begin{array}{c} (R_s)_{0\le s\le m}:\\ R_s \ni \omega (s) \forall s \end{array}} \prod _{t=0}^m W(R_t)\prod _{uv\in [0,m]}[1+U_{uv}]\nonumber \\&\le \sum _{\omega :0\overset{m}{\rightarrow }u}W(\omega )  \sum _{R_m\ni \omega (m)}  W(R_m)\sum _{R_0\ni \omega (0)}W(R_0) \sum _{\begin{array}{c} (R_s)_{1\le s\le m-1}:\\ R_s \ni \omega (s) \forall s \end{array}}\prod _{t=1}^{m-1} W(R_t)\prod _{uv\in [1,m-1]}[1+U_{uv}]\nonumber \\&\le \sum _{\omega :0\overset{m}{\rightarrow }u}W(\omega ) \rho ^2\sum _{\begin{array}{c} (R_s)_{1\le s\le m-1}:\\ R_s \ni \omega (s) \forall s \end{array}}\prod _{t=1}^{m-1} W(R_t)\prod _{uv\in [1,m-1]}[1+U_{uv}]=\rho ^2 h_m(u). \end{aligned}$$

Let us recall partially from [17, Lemma 5.4],^4^ in which the function $\varrho :\mathbb {Z}^d \rightarrow \mathbb {R}_+$ is defined by $\varrho (x)=\rho \mathbb {P}(x \in \mathcal {T})$.

#### Lemma 4.19

Fix $d>8$. There exists $L_0(d)$ such that for all $L\ge L_0$: For any $l\ge 1$ there exists $C_l>0$ such that for all $k\in \{0,1,2,3,4\}$ and ${m}^{(l)}=(m_1,\dots , m_l)\in \mathbb {Z}_+^l$ and $m=\sum _{i=1}^l m_i$, then

$$\begin{aligned} \left\| *_{i=1}^l h_{m_i} *\varrho ^{(k)}\right\| _{\infty }\le \frac{ C_l}{m^{\frac{d-2k}{2}}}, \text { and } \left\| *_{i=1}^l h_{ m_i} \right\| _1 \le C_l. \end{aligned}$$

**Fig. 12:**
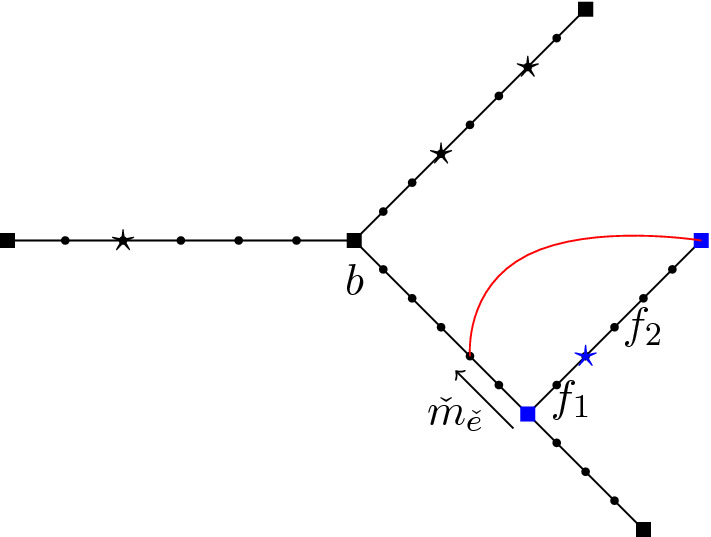
A skeleton network $\mathcal {N}^{+}$ with a bond in $\mathcal {R}$. This bond has endpoints in the marked edge $\check{e}\in \mathcal {N}^+$ and the “phantom” marked edge $\check{e}'\in \mathcal {N}^{++}$ of lengths $\check{n}_{\check{e}}=6$ and $\check{n}'_{\check{e}'}=0$ respectively. We write $st(\check{e},\check{e}',\check{m}_{\check{e}},\check{m}'_{\check{e}'})$ for this bond. Here, $\check{m}_{\check{e}}=2$ is indicated, while $\check{m}'_{\check{e}'}=0$. The set of marked edges $\varvec{E}^+_{\check{e}, \check{e}'}$ on the path from $\check{e}$ to $\check{e}'$ is $\{f_1,f_2\}$ from  to  and  to  as indicated

For a given skeleton network $\mathcal {N}^+$, let $r_+=\#\varvec{E}(\mathcal {N}^+)$. If there is a bond $uu'$ covering two special points then either we can find two non-neighbouring marked edges $\check{e}\ni u$ and $\check{e}'\ni u'$, or (at least) one of $u,u'$ is a leaf of $\mathcal {N}^+$. In order to accommodate the latter cases, for the proof of Lemma 4.14(e:$\mathcal {R}$) it is notationally convenient to adjoin to each leaf in $\mathcal {N}^+$ a “phantom” marked edge of length 0, and write $\varvec{E}(\mathcal {N}^{++})$ for this enlarged set of marked edges. For marked edges $\check{e},\check{e}'\in \varvec{E}(\mathcal {N}^{++})$ write $\check{e}\sim \check{e}'$ if they are adjacent, and $\check{e}\not \sim \check{e}'$ otherwise. Recall from (4.4) that in the notation $U_{st}$, *st* is a pair of vertices in $\mathcal {N}^+$. For non-adjacent marked edges $\check{e},\check{e}'\in \varvec{E}(\mathcal {N}^{++})$ and $m\le \check{n}_{\check{e}}$, and $m'\le \check{n}'_{\check{e}'}$, write $st(\check{e},\check{e}',m,m')$ to denote the pair of vertices in $\mathcal {N}^+$ corresponding to the *m*-th vertex along marked edge $\check{e}$ in the direction away from $\check{e}'$ and the $m'$-th vertex along marked edge $\check{e}'$ in the direction away from $\check{e}'$. If e.g. $\check{e}$ was one of the phantom marked edges then $\check{n}_{\check{e}}=0$ and the relevant vertex is actually the leaf that $\check{e}$ was adjoined to. See e.g. Fig. 12.

For $0\le a\le b\le \check{n}_{\check{e}}$, write $\check{e}[a,b]$ to denote that part of the marked edge $\check{e}$ consisting of the *a*-th to the *b*-th vertices (with ordering directed away from $\check{e}'$ as above) and similarly define $\check{e}'[a',b']$ for $0\le a'\le b'\le \check{n}'_{\check{e}'}$.

*Proof of Lemma* 4.14(e:$\mathcal {R}$). In the definition of $\phi _{\mathcal {N}^+}^{\mathcal {R}}$ (see (4.6)), we can see that

$$\begin{aligned} 1-\prod _{vv'\in \mathcal {R}} [1+U_{vv'}] \le \sum _{\begin{array}{c} \check{e},\check{e}'\in \varvec{E}(\mathcal {N}^{++}),\\ \check{e}\not \sim \check{e}' \end{array} }\sum _{\begin{array}{c} \check{m}_{\check{e}}\le \check{n}_{\check{e}}, \\ \check{m}_{\check{e}'}\le \check{n}_{\check{e}'} \end{array}} -U_{st(\check{e},\check{e}',\check{m}_{\check{e}},\check{m}'_{\check{e}'})}, \end{aligned}$$

since if there is a bond $uu'$ covering two special points then we can find two non-neighbouring marked edges in $\varvec{E}(\mathcal {N}^{++})$ containing *u* and $u'$ respectively (for more details see [17, Section 6.5.1]).

For a marked edge $\check{f}\in \varvec{E}(\mathcal {N}^+)$, write ${{\overline{\prod }}}_{uv\in \check{f}}$ for a product over pairs of distinct vertices *u*, *v* in the interior of $\check{f}$ (i.e. *u*, *v* are vertices in $\check{f}$ that are not the endvertices of $\check{f}$). For non-adjacent marked edges $\check{e},\check{e}'$ and $0\le a\le b\le \check{n}_{\check{e}}$ as above, write ${{\overline{\prod }}}_{uv\in \check{e}[a,b]}$ for a product over pairs of distinct *u*, *v* in $\check{e}[a,b]$ that are neither endvertex of this set, and similarly define ${{\overline{\prod }}}_{uv\in \check{e}'[a',b']}$ for $0\le a'\le b'\le \check{n}'_{\check{e}'}$.

Fix distinct $\check{e}\not \sim \check{e}'$ in $\varvec{E}(\mathcal {N}^{++})$ and $\check{m}_{\check{e}}\le \check{n}_{\check{e}}, \check{m}_{\check{e}'}\le \check{n}_{\check{e}'}$. By ignoring the constraints of non-intersection between various $R_i$ (bounding some $1+U_{uu'}$ by 1), we obtain

$$\begin{aligned} \prod _{uu'\in \mathcal {R}^c} [1+U_{uu'}]&\le \prod _{\check{f}\in \varvec{E}(\mathcal {N}^+) \setminus \{\check{e},\check{e}'\}} \Bigg [\mathop {{\overline{\prod }}}\limits _{uu'\in \check{f}} [1+U_{uu'}]\Bigg ]\\&\quad \times \Bigg [\mathop {{\overline{\prod }}}\limits _{u_0u'_0\in \check{e}[0,\check{m}_{\check{e}}]} [1+U_{u_0u'_0}]\Bigg ] \Bigg [\mathop {{\overline{\prod }}}\limits _{u_1u'_1\in \check{e}[\check{m}_{\check{e}},\check{n}_{\check{e}}]} [1+U_{u_1u'_1}]\Bigg ] \\&\quad \times \Bigg [\mathop {{\overline{\prod }}}\limits _{u_2u'_2\in \check{e}'[0,\check{m}'_{\check{e}'}]} [1+U_{u_2u'_2}]\Bigg ] \Bigg [\mathop {{\overline{\prod }}}\limits _{u_3u'_3\in \check{e}'[\check{m}'_{\check{e}'},\check{n}'_{\check{e}'}]} [1+U_{u_3u'_3}]\Bigg ] . \end{aligned}$$

where e.g. if $\check{m}_{\check{e}}\in \{0,\check{n}_{\check{e}}\}$ then the corresponding empty product is 1. (Note that this kind of approach is used to prove (2.25) as well as the more general statement appearing in Remark 2.12.) Using the above inequalities we can see that

4.20 $$\begin{aligned} 0&\le \Bigl (\prod _{uu'\in \mathcal {R}^c} [1+U_{uu'}]\Bigr ) \Bigl (1-\prod _{vv'\in \mathcal {R}} [1+U_{vv'}]\Bigr )\nonumber \\&\le \sum _{\begin{array}{c} \check{e},\check{e}'\in \varvec{E}(\mathcal {N}^{++}),\\ \check{e}\not \sim \check{e}' \end{array} }\sum _{\begin{array}{c} \check{m}_{\check{e}}\le \check{n}_{\check{e}}, \\ \check{m}_{\check{e}'}\le \check{n}_{\check{e}'} \end{array}} [ -U_{st(\check{e},\check{e}',\check{m}_{\check{e}},\check{m}'_{\check{e}'})}] \prod _{\check{f}\in \varvec{E}(\mathcal {N}^+) \setminus \{\check{e},\check{e}'\}} \Bigg [\mathop {{\overline{\prod }}}\limits _{uu'\in \check{f}} [1+U_{uu'}]\Bigg ]\nonumber \\&\quad \times \Bigg [\mathop {{\overline{\prod }}}\limits _{u_0u'_0\in \check{e}[0,\check{m}_{\check{e}}]} [1+U_{u_0u'_0}]\Bigg ] \Bigg [\mathop {{\overline{\prod }}}\limits _{u_1u'_1\in \check{e}[\check{m}_{\check{e}},\check{n}_{\check{e}}]} [1+U_{u_1u'_1}]\Bigg ] \nonumber \\&\quad \times \Bigg [\mathop {{\overline{\prod }}}\limits _{u_2u'_2\in \check{e}'[0,\check{m}'_{\check{e}'}]} [1+U_{u_2u'_2}]\Bigg ] \Bigg [\mathop {{\overline{\prod }}}\limits _{u_3u'_3\in \check{e}'[\check{m}'_{\check{e}'},\check{n}'_{\check{e}'}]} [1+U_{u_3u'_3}]\Bigg ], \end{aligned}$$

Note that if e.g. $\check{e}$ is a phantom marked edge then the corresponding sum over $\check{m}_{\check{e}}$ contains only the value $0=\check{n}_{\check{e}}$.

Now, the $\omega$ in (4.6) can be broken up at every special point and at the two vertices corresponding to $st(\check{e},\check{e}',m,m')$. The graph then becomes broken up into (at most) $r_++2$ segments. Let us now introduce the set $\varvec{E}^+_{\check{e}, \check{e}'}$ of marked edges which connect (but do not include) $\check{e}$ to $\check{e}'$ which is non-empty since $\check{e}$ and $\check{e}'$ are not neighbours, and $\bar{\varvec{E}}^+_{\check{e}, \check{e}'}=\varvec{E}^+_{\check{e}, \check{e}'}\cup \{\check{e},\check{e}'\}$. Letting $\check{\varvec{y}}_{\check{e},\check{e}'}=(\check{y}_{\check{f}})_{ \check{f} \in \varvec{E}^+_{\check{e}, \check{e}'}}$ with each $\check{y}_{\check{f}}\in \mathbb {Z}^d$ we have from (4.20) and Remark 1.1 that (cf. [17, (6.18)])

$$\begin{aligned}&\sum _{\check{\varvec{y}}}\left| \phi _{\mathcal {N}^+}^{\mathcal {R}}(\check{\varvec{y}})\right| \\&\quad \le \rho ^{2(r_++2)}\sum _{\begin{array}{c} \check{e},\check{e}'\in \varvec{E}(\mathcal {N}^{++}),\\ \check{e}\not \sim \check{e}' \end{array} }\sum _{\begin{array}{c} \check{m}_{\check{e}}\le \check{n}_{\check{e}}, \\ \check{m}_{\check{e}'}\le \check{n}_{\check{e}'} \end{array}} \Bigl (\prod _{\check{f}'\in \varvec{E}(\mathcal {N}^+) \setminus \bar{\varvec{E}}^+_{\check{e}, \check{e}'}} \sum _{\check{y}_{\check{f}'}} h_{\check{n}_{\check{f}'}}(\check{y}_{\check{f}'})\Bigr ) \sum _{\check{\varvec{y}}_{\check{e},\check{e}'}}\sum _{u_{\check{e}},u_{\check{e}'}} \Bigg [ \prod _{\check{f}\in \varvec{E}^+_{\check{e}, \check{e}'}} h_{\check{n}_{\check{f}}}(\check{y}_{\check{f}})\\&\quad \times h_{\check{m}_{\check{e}}}(u_{\check{e}})h_{\check{m}_{\check{e}'}}(u_{\check{e}'})\varrho ^{(2)}\Big (u_{\check{e}}+u_{\check{e}'}+\sum _{\check{f}\in \varvec{E}^+_{\check{e}, \check{e}'}} \check{y}_{\check{f}}\Big ) \sum _{\check{y}_{\check{e}}}h_{\check{n}_{\check{e}}-\check{m}_{\check{e}}}(\check{y}_{\check{e}}-u_{\check{e}}) \sum _{\check{y}_{\check{e}'}}h_{\check{n}_{\check{e}'}-\check{m}_{\check{e}'}}(\check{y}_{\check{e}'}-u_{\check{e}'})\Bigg ]. \end{aligned}$$

This arises because e.g. if $\check{f}$ and $\check{f}'$ are two distinct marked edges for which there is no $U_{st}$ term appearing anywhere in (4.20) with *s* and *t* vertices of $\check{f}$ and $\check{f}'$ respectively, then the corresponding segments of $\omega$ (and the sets of lattice trees $R_\cdot$ hanging off them) have been decoupled. Segments of $\omega$ and corresponding elements of $\check{\varvec{y}}$ can then be summed over “independently”, with factors of $\rho$ arising at endvertices, similarly to (4.19). Similarly, the presence of the term $[-U_{st(\cdot ,\cdot ,\cdot ,\cdot )}]$ in (4.20) forces two corresponding trees $R_\cdot$ to intersect, which yields the $\varrho ^{(2)}$ term above. Recalling that $\sum _{\check{y}} h_n(\check{y}) \le C_1$ for any *n* by Lemma 4.19 we see that

$$\begin{aligned} \sum _{\check{\varvec{y}}}\left| \phi _{\mathcal {N}^+}^{\mathcal {R}}(\check{\varvec{y}})\right| \le \rho ^{2(r_++2)}C_1^{r_+}\sum _{\begin{array}{c} \check{e},\check{e}'\in \varvec{E}(\mathcal {N}^{++}),\\ \check{e}\not \sim \check{e}' \end{array} }\sum _{\begin{array}{c} \check{m}_{\check{e}}\le \check{n}_{\check{e}}, \\ \check{m}_{\check{e}'}\le \check{n}_{\check{e}'} \end{array}} (h_{\check{m}_{\check{e}}}*h_{\check{m}_{\check{e}'}} *\varrho ^{(2)} \underset{\check{f}\in \varvec{E}^+_{\check{e}, \check{e}'}}{*} h_{\check{n}_{\check{f}}})(0). \end{aligned}$$

(The power of $C_1$ is $r_+-\#\bar{\varvec{E}}^+_{\check{e}, \check{e}'}+2\le r_+$, and so assuming $C_1\ge 1$ without loss of generality, the above follows.) The notation in the last convolution above means that there is one term in the convolution for each $\check{f}\in \varvec{E}^+_{\check{e}, \check{e}'}$. By Lemma 4.19 with $k=2$ and $l=l_+:=2+\#\varvec{E}^+_{\check{e}, \check{e}'}$ we have that for $n_{\check{e},\check{e}'}=\sum _{\check{f}\in \varvec{E}^+_{\check{e}, \check{e}'}}\check{n}_{\check{f}}$,

$$\begin{aligned} \sum _{\check{\varvec{y}}}\left| \phi _{\mathcal {N}^{+}}^{\mathcal {R}}(\check{\varvec{y}})\right|&\le \rho ^{2(r_++2)}C_1^{r_+}\sum _{\begin{array}{c} \check{e},\check{e}'\in \varvec{E}(\mathcal {N}^{++}),\\ \check{e}\not \sim \check{e}' \end{array} }\sum _{\begin{array}{c} \check{m}_{\check{e}}\le \check{n}_{\check{e}}, \\ \check{m}_{\check{e}'}\le \check{n}_{\check{e}'} \end{array}} \frac{ C_{l_+ }}{(\check{m}_{\check{e}}+\check{m}_{\check{e}'}+n_{\check{e},\check{e}'})^{\frac{d-4}{2}}} \\&\le C(r_+) \sum _{\begin{array}{c} \check{e},\check{e}'\in \varvec{E}(\mathcal {N}^{++}),\\ \check{e}\not \sim \check{e}' \end{array} } \frac{1}{n_{\check{e},\check{e}'}^{\frac{d-8}{2}}} \\&\le C'(r_+) \sum _{e=1}^{2r-1}\sum _{j=1}^{j_e} \frac{1}{\check{n}_{e,j}^{\frac{d-8}{2}}}. \end{aligned}$$

$$\square $$

*Proof of Lemma* 4.14(e:$\pi$). Similarly to Lemma 4.18 (but note the change in the first summation) we have that

$$\begin{aligned} \phi _{\mathcal {N}^+}^{\pi }(\check{\varvec{y}})=\sum _{{m} \in \overline{\mathcal {H}}_{\check{\varvec{n}}_b}} \sum _{{u}\in (\mathbb {Z}^d)^3}\pi _{{m}}({u}) \prod _{j=1}^3 \Big (z_D \sum _{v_j} D(v_j- u_j)t_{\mathcal {N}_{j,{m}}^+}(\check{\varvec{y}}^{v_j, j})\Big ). \end{aligned}$$

Therefore, for any $\check{\varvec{y}}\in (\mathbb {Z}^d)^{r_+}$,

$$\begin{aligned} \left| \phi _{\mathcal {N}^+}^{\pi }(\check{\varvec{y}})\right|&\le C\left| \sum _{{m} \in \overline{\mathcal {H}}_{\check{\varvec{n}}_b}} \sum _{{u} } \pi _{{m}}({u}) \sum _{\check{\varvec{y}}} \prod _{j=1}^3 \sum _{v_j} D(v_j-u_j) t_{\mathcal {N}^+_{j,{m}}}(\check{\varvec{y}}^{v_j,j})\right| \\&\le C\sum _{N=1}^{\infty }\sum _{{m} \in \overline{\mathcal {H}}_{\check{\varvec{n}}_b}} \sum _{{u} } \pi _{{m}}^{\scriptscriptstyle (N)}({u}) \sum _{\check{\varvec{y}}} \prod _{j=1}^3 \sum _{v_j} D(v_j-u_j) t_{\mathcal {N}^+_{j,{m}}}(\check{\varvec{y}}^{v_j,j}). \end{aligned}$$

Using a generalisation of  (2.25) as in Remark 2.12, and then (4.13) and (4.14), we have

$$\begin{aligned} \sum _{\check{\varvec{y}}}\left| \phi _{\mathcal {N}^+}^{\pi }(\check{\varvec{y}})\right| \le C\sum _{N=1}^{\infty }\sum _{{m} \in \overline{\mathcal {H}}_{{\check{\varvec{n}}}^{ b}}} \sum _{{u} } \pi _{{m}}^{\scriptscriptstyle (N)}({u})K_0^{r_+}\le C\sum _{N=1}^{\infty }\sum _{k=1}^3 \sum _{{m}:m_k\ge \check{n}_{\check{e}_k} /3} B_N({m}) \le \sum _{k=1}^3 \frac{C'}{\check{n}_{\check{e}_k}^{\frac{d-8}{2}}}. \end{aligned}$$

The result follows. $\square$

The proof of Lemma 4.14(e:*b*) is again an adaptation of the proof in [17] (specifically in [17, Section 6.5.3]). Here we will indicate the changes to the argument required for the present setting of a marked skeleton network. We start by adapting [17, Definition 2.2]. Given a graph $\Gamma \in \mathcal {E}^b_{\mathcal {N}^+}$ on $\mathcal {N}^+$, a special point *v* of $\mathcal {N}^+$ and a marked edge *e* of which *v* is an endpoint, we define the *bond associated to* *e* *at* *v* as follows: If there is no bond in $\Gamma$ covering *v* that has an endpoint strictly on *e* then there is no bond associated to *e* at *v*. Otherwise from the set of such bonds we choose the one whose endpoint in *e* is farthest from *v*. If this is not unique then we choose from this set one according to a fixed but arbitrary rule (e.g. choose from those whose other endpoint is strictly on some edge $e'$ of smallest label the one whose endpoint on $e'$ is farthest from *v* in this direction).

*Proof of Lemma* 4.14(e:*b*). Recall that

4.21 $$\begin{aligned} \phi _{\mathcal {N}^+}^{b}(\check{\varvec{y}})=\sum _{\omega \in \Omega _{\mathcal {N}^+}(\check{\varvec{y}})} W(\omega ) \sum _{\begin{array}{c} (R_s)_{s \in \mathcal {N}^+}:\\ R_s\ni \omega (s) \forall s \in \mathcal {N}^+ \end{array}} \prod _{t \in \mathcal {N}^+}W(R_t) \sum _{\Gamma \in \mathcal {E}_{\mathcal {N}^+}^{-\mathcal {R},b}} \prod _{vv'\in \Gamma } U_{vv'}. \end{aligned}$$

Recall also that $(\check{e}_i)_{i=1}^3$ are the marked edges adjacent to *b* and denote their end vertices (other than *b*) as $(\check{v}_i)_{i=1}^3$, which are special points.

For $$F\subset \{1,2,3\}$$ let

$$\begin{aligned} \mathcal {E}^b_{F,\mathcal {N}^+}=\big \{\Gamma \in \mathcal {E}_{\mathcal {N}^+}^{-\mathcal {R},b}: \forall i \in F, \mathcal {A}_b(\Gamma ) \text { contains a nearest neighbour of }\check{v}_i\big \}. \end{aligned}$$

Note that if $F \ne \{1,2,3\}$ this set may include $\Gamma$ for which some $\mathcal {A}_b(\Gamma )$ also contains a nearest neighbour of $\check{v}_i$ for some $i \in \{1,2,3\}\setminus F$. Inclusion–exclusion over the sets *F* gives

4.22 $$\begin{aligned} \Big |\sum _{\Gamma \in \mathcal {E}_{\mathcal {N}^+}^{-\mathcal {R},b}} \prod _{vv'\in \Gamma } U_{vv'}\Big |\le \sum _{F\ne \varnothing }\Big |\sum _{\Gamma \in \mathcal {E}^b_{F,\mathcal {N}^+}}\prod _{vv'\in \Gamma } U_{vv'}\Big |. \end{aligned}$$

Given $\Gamma \in \mathcal {E}^b_{F,\mathcal {N}^+}$ we define a subgraph $\Gamma _F\subset \Gamma$ to be the set of bonds $st\in \Gamma$ such that

- *st* is the bond associated to one of the marked edges $\check{e}_i$ at *b*, for some $i \in F$, or
- *st* is the bond associated to one of the marked edges $\check{e}_i$, at $\check{v}_i$ where $i \in F$, or
- *st* are both vertices in the marked edge $\check{e}_i$ for some $i\in F$.

Let $\mathcal {S}_F$ denote the largest connected subnetwork of $\mathcal {N}^+$ containing *b* that is covered by $\Gamma _F$. Then $\mathcal {S}_F$ is a star-shaped network of degree 3 or less (with branch point *b*) and $\Gamma _F$ is a connected graph on $\mathcal {S}_F$. Moreover $\mathcal {S}_F$ contains at most $\#F+1$ special points of $\mathcal {N}^+$ (one of which is *b*) since $\Gamma$ contains no bonds in $\mathcal {R}$. Note that the length of branch *i* of $\mathcal {S}_F$ is at least $\check{n}_i-1$. Let $\mathbb {S}_F(\mathcal {N}^+)$ denote (for fixed *F*) the set of possible $\mathcal {S}_F$ that can arise as above from graphs $\Gamma \in \mathcal {E}^b_{F,\mathcal {N}^+}$. It follows that

4.23 $$\begin{aligned} \sum _{\Gamma \in \mathcal {E}^b_{F,\mathcal {N}^+}}\prod _{vv'\in \Gamma } U_{vv'}=\sum _{\mathcal {S}\in \mathbb {S}_F(\mathcal {N}^+)}\sum _{\begin{array}{c} \Gamma \in \mathcal {E}^b_{F,\mathcal {N}^+}:\\ \mathcal {S}_F(\Gamma )=\mathcal {S} \end{array}}\prod _{vv'\in \Gamma } U_{vv'}. \end{aligned}$$

Now we may proceed as in [17, (6.23)–(6.28)], which we briefly discuss in the following paragraph but direct the reader to [17] for details. For fixed *F* and $\mathcal {S}\in \mathbb {S}_F(\mathcal {N}^+)$ we have the notion of a lace on $\mathcal {S}$ containing *N* bonds and the set of bonds, $\mathcal {C}(L)$, which are compatible with the lace *L*, as described after (4.11). Similarly, given *F*, and $\Gamma \in \mathcal {E}^b_{F,\mathcal {N}^+}$ such that $\mathcal {S}_F(\Gamma )=\mathcal {S}$ we have the lace associated to the subgraph $\Gamma _F$, which is a connected graph on $\mathcal {S}$. Thus, as in [17, (6.23)–(6.24)], we can write (4.23) as

4.24 $$\begin{aligned} \sum _{N=1}^\infty (-1)^N \sum _{\mathcal {S}\in \mathbb {S}_F(\mathcal {N}^+)}\sum _{L \in \mathcal {L}_{\mathcal {S}}^{(N),F}}\Big [\prod _{st\in L}(-U_{st})\Big ]\Bigg [\sum _{\begin{array}{c} \Gamma \in \mathcal {E}^b_{F,\mathcal {N}^+}:\\ \mathcal {S}_F(\Gamma )=\mathcal {S},\\ \varvec{L}(\Gamma _F)=L \end{array}}\prod _{vv'\in \Gamma } U_{vv'}\Bigg ], \end{aligned}$$

where the sum over *L* is a sum over (a certain subclass of all) laces on $\mathcal {S}$ containing exactly *N* bonds (for the definition of this subclass see [17, definition prior to (6.23)]). The last two pages of [17] show how to deal with the “messy” final sum in (4.24), by breaking the sum over $\Gamma$ into three sets: (i) sets of bonds on $\mathcal {S}$ that are compatible with *L*; (ii) sets of bonds that live on $\mathcal {N}^+\setminus \mathcal {S}$; and (iii) sets of bonds *st* with one endpoint in $\mathcal {S}$ and one in $\mathcal {N}^+\setminus \mathcal {S}$ for which $\mathcal {S}_F(L \cup \{st\})=\mathcal {S}$ (in each case bonds in $\mathcal {R}$ are excluded). Using this decomposition we see that (4.24) is equal to

4.25 $$\begin{aligned}&\sum _{N=1}^\infty (-1)^N \sum _{\mathcal {S}\in \mathbb {S}_F(\mathcal {N}^+)}\sum _{L \in \mathcal {L}_{\mathcal {S}}^{(N),F}}\Big [\prod _{st\in L}(-U_{st})\Big ]\Bigg [\sum _{\begin{array}{c} \Gamma \in \mathcal {G}_{\mathcal {\mathcal {S}}}^{-\mathcal {R},\text {con}}:\\ \varvec{L}(\Gamma )=L \end{array}}\prod _{st\in \Gamma }U_{st}\Bigg ] \Bigg [\sum _{\Gamma ' \in \mathcal {G}_{\mathcal {N}^+\setminus \mathcal {S}}^{-\mathcal {R}}}\prod _{st\in \Gamma '}U_{st}\Bigg ]\nonumber \\&\quad \quad \times \Bigg [\sum _{\begin{array}{c} \Gamma ^* \in \mathcal {G}^{-\mathcal {R}}_{\mathcal {S},\mathcal {N}^+\setminus \mathcal {S}}:\\ \mathcal {S}_F(L\cup \Gamma ^*)=\mathcal {S} \end{array}}\prod _{st\in \Gamma } U_{st}\Bigg ]\nonumber \\&=\sum _{N=1}^\infty (-1)^N \sum _{\mathcal {S}\in \mathbb {S}_F(\mathcal {N}^+)}\sum _{L \in \mathcal {L}_{\mathcal {S}}^{(N),F}}\Big [\prod _{st\in L}(-U_{st})\Big ]\Big [\prod _{st \in \mathcal {C}(L)}[1+U_{st}]\Big ]\Big [\prod _{st \in \mathcal {N}^+\setminus \mathcal {S}}[1+U_{st}]\Big ] \end{aligned}$$

4.26 $$\begin{aligned}&\quad \quad \times \prod _{\begin{array}{c} s\in \mathcal {S},t \in \mathcal {N}^+\setminus \mathcal {S}:\\ \mathcal {S}_F(L \cup \{st\})=\mathcal {S} \end{array}}[1+U_{st}]. \end{aligned}$$

We bound the absolute value of the above by simply removing the factors $(-1)^N$ (everything else is non-negative). Then we can ignore the last product (4.26) (bound it by 1) and get an upper bound. Similarly we can discard any part of the last product in (4.25) to get an upper bound. For the latter we throw away all *st* such that *s* and *t* are on different connected components of $\mathcal {N}^+\setminus \mathcal {S}$. We deduce from (4.21), (4.22), and the above that

4.27 $$\begin{aligned}&\sum _{\check{\varvec{y}}}|\phi _{\mathcal {N}^+}^{b}(\check{\varvec{y}})|\nonumber \\&\quad \le \sum _{F\ne \varnothing }\sum _{N=1}^\infty \sum _{\check{\varvec{y}}}\sum _{\omega \in \Omega _{\mathcal {N}^+}(\check{\varvec{y}})} W(\omega ) \sum _{\begin{array}{c} (R_s)_{s \in \mathcal {N}^+}:\\ R_s\ni \omega (s) \forall s \in \mathcal {N}^+ \end{array}} \prod _{t \in \mathcal {N}^+}W(R_t)\nonumber \\&\quad \qquad \times \sum _{\mathcal {S}\in \mathbb {S}_F(\mathcal {N}^+)}\Bigg \{\sum _{L \in \mathcal {L}_{\mathcal {S}}^{(N),F}}\Big [\prod _{st\in L}(-U_{s,t})\Big ]\Big [\prod _{st \in \mathcal {C}(L)}[1+U_{st}]\Big ]\Bigg \}\prod _{j=1}^{\Delta _{\mathcal {N}^+\setminus \mathcal {S}}}\Big [\prod _{st \in (\mathcal {N}^+\setminus \mathcal {S})_j}[1+U_{st}]\Big ], \end{aligned}$$

where $\Delta _{\mathcal {N}^+\setminus \mathcal {S}}$ denotes the number of disjoint components of $\mathcal {N}^+\setminus \mathcal {S}$ and the components are denoted by $(\mathcal {N}^+\setminus \mathcal {S})_j$. Here, the components $\mathcal {S}$, and $(\mathcal {N}^+\setminus \mathcal {S})_j$ for all *j* have now been decoupled, because there are no $U_{st}$ terms where *s* and *t* are on different components. Recalling (4.11), the term in curly brackets in (4.27) (in combination with the part of $\omega$ and the trees $R_\cdot$ corresponding to $\mathcal {S}$) is the quantity that gives rise to $\pi _{{m}}^{\scriptscriptstyle (N)}$ (where the $m_i$ are the lengths of the branches of $\mathcal {S}$) except that we are summing over a restricted set of laces containing *N* bonds. But we can also sum over all $L\in \mathcal {L}^{(N)}(\mathcal {S})$, the set of laces on $\mathcal {S}$ with exactly *N* bonds, to get an upper bound. This gives rise to a bound

4.28 $$\begin{aligned} \sum _{\check{\varvec{y}}}\left| \phi _{\mathcal {N}^+}^{b}(\check{\varvec{y}})\right| \le \sum _{F \ne \varnothing }\sum _{N=1}^{\infty }\sum _{\begin{array}{c} {m}:\\ m_i\ge \check{n}_i-1 \forall i \in F \end{array}}\sum _{{u}}\pi _{{m}}^{\scriptscriptstyle (N)}({u}) C(r_+), \end{aligned}$$

where we note that the sum over ${m}$ arises from the sum over $\mathcal {S}$ seen in previous expressions, and the constant $C(r_+)$ arises from the generalisation of (2.25) noted in Remark 2.12. Finally use Proposition 4.17 to see that (4.28) is at most

$$\begin{aligned} C\sum _{F \ne \varnothing }\sum _{N=1}^{\infty }\sum _{\begin{array}{c} {m}:\\ m_i\ge \check{n}_i-1 \\ \forall i \in F \end{array}}B_N({m})\le \sum _{i=1}^3\frac{C}{\check{n}_i^{\frac{d-8}{2}}}. \end{aligned}$$

This proves the result. $\square$

## Data Availability

Data sharing is not applicable to this article as no datasets were generated or analysed during the current study.

## Footnotes

1 For oriented percolation, one should choose a notion of historical paths e.g. each site in the cluster of the origin chooses its parent uniformly at random from all possible parents in the cluster.

2 Our constant $C_A$ is equal to $A'=A/\rho$ from [17] and our constant $C_V$ is $V\rho ^2$ from [17].

3 Super-Brownian motion is not expected to arise as the scaling limit for $d<8$.

4 [17, Lemma 5.4] is stated under some additional hypotheses (display (3.24) in that paper). Nevertheless [17, Theorem 3.7] shows that the assumptions (3.24) are met.
